# The Endophytic Mycobiome of European Ash and Sycamore Maple Leaves – Geographic Patterns, Host Specificity and Influence of Ash Dieback

**DOI:** 10.3389/fmicb.2018.02345

**Published:** 2018-10-24

**Authors:** Markus Schlegel, Valentin Queloz, Thomas N. Sieber

**Affiliations:** ^1^Department of Environmental Systems Science, Institute of Integrative Biology, Forest Pathology and Dendrology, ETH Zurich, Zurich, Switzerland; ^2^Swiss Federal Institute for Forest, Snow and Landscape Research WSL, Swiss Forest Protection, Birmensdorf, Switzerland

**Keywords:** endophytic fungi, ash dieback, invasive pathogen, cryptic extinction, emerging disease, fungal-specific primers, mock community

## Abstract

The European ash (*Fraxinus excelsior*) is threatened by the introduced ascomycete *Hymenoscyphus fraxineus*, the causal agent of ash dieback. Endophytic fungi are known to modulate their host’s resistance against pathogens. To understand possible consequences of ash dieback on the endophytic mycobiome, *F. excelsior* leaves were collected in naturally regenerated forests and the fungal communities analyzed by classic culture and Illumina amplicon sequencing using a newly developed and validated fungal-specific primer. Collections were done in the area infested by ash dieback north of the Alps, and in the disease free area on the south side. Sycamore maple (*Acer pseudoplatanus*) was additionally collected, as well as the flowering ash (*F. ornus*), which occurs naturally in the south and shows tolerance to ash dieback. Both cultivation and amplicon sequencing revealed characteristic endophytic fungal communities dominated by several strictly host specific *Venturia* species. On *A. pseudoplatanus*, a hitherto undescribed *Venturia* species was identified. Due to its dominance on *F. excelsior*, *V. fraxini* is unlikely to go extinct in case of reduced host densities. A majority of species was not strictly host specific and is therefore likely less affected by ash dieback in the future. Still, shifts in community structure and loss of genetic diversity cannot be excluded. The potentially endangered endophyte *Hymenoscyphus albidus* was rarely found. In addition to host specificity, species with preferences for leaf laminae or petioles were found. We also detected considerable geographical variation between sampling sites and clear differences between the two sides of the Alps for endophytes of *F. excelsior*, but not *A. pseudoplatanus*. Since sycamore maple is not affected by an epidemic, this could point toward an influence of ash dieback on ash communities, although firm conclusions are not possible because of host preferences and climatic differences. Furthermore, the mycobiota of *F. excelsior* trees with or without dieback symptoms were compared, but no clear differences were detected. Besides methodical refinement, our study provides comprehensive data on the ash mycobiome that we expect to be subject to changes caused by an emerging disease of the host tree.

## Introduction

The European ash (*Fraxinus excelsior* L.) is an important hardwood in Europe (Dobrowolska et al., 2011) and the third most common broadleaved tree species in Switzerland after beech and sycamore maple (Abegg et al., 2014). It is currently affected by the invasive ash dieback pathogen, *H. fraxineus* (T. Kowalski) Baral, Queloz & Hosoya (Queloz et al., 2011; Baral et al., 2014), which causes massive tree mortality and represents a serious threat for ash trees of all age classes (Gross et al., 2014a). The pathogen has been introduced from East Asia to Poland in 1992 (Zhao et al., 2012; Gross et al., 2014b). Since then, it spread in all geographic directions and meanwhile occurs in most of the distribution area of *F. excelsior* (Vasaitis and Enderle, 2017). Infections are initiated on leaves by wind-borne ascospores, proceeding into the shoot where they cause serious necroses leading to wilting and dieback. Apothecia form on rachises of fallen leaves during the following summer, thus enabling new infections (Gross et al., 2014a).

While the consequences of the disease on ash trees are well understood, less is known about possible interactions of the disease with microorganisms associated with ash trees. Endophytic fungi, normally invisible to the naked eye, form distinct communities in healthy tissues of virtually all plant species. Their ecological roles may comprise mutualism, commensalism, latent pathogenicity, and parasitism, and interactions with their hosts are often poorly understood (Schulz and Boyle, 2006; Sieber, 2007; Porras-Alfaro and Bayman, 2011). Tree endophytes are horizontally transmitted by spores with colonization of the leaves increasing throughout the growing season (Helander et al., 1993; Wilson and Carroll, 1994; Scholtysik et al., 2012). *F. excelsior* leaves and shoots are known to be inhabited by diverse communities of fungal endophytes (Unterseher et al., 2007; Chen, 2012; Scholtysik et al., 2012; Davydenko et al., 2013; Schlegel et al., 2016; Cross et al., 2017; Haňáčková et al., 2017a; Kosawang et al., 2017). The reproduction and dispersal of these endophytes are likely to be influenced by direct interaction of fungal thalli, or by reduced host densities, leading to shifts in community structure and possibly species extinctions following disturbances (Mack et al., 2000; Koh et al., 2004; Dobson et al., 2008; Keesing et al., 2010). Since *H. fraxineus* is able to complete its entire life cycle on ash leaves, endophytes present in the leaves may interact with the pathogen in various ways. Endophytic fungi are known to protect their hosts against abiotic stress (Rodriguez and Redman, 2008) and to influence host resistance against pathogens, both positively and negatively (Shoresh et al., 2010; Porras-Alfaro and Bayman, 2011; Busby et al., 2016). Given the growing knowledge about protective effects, endophytes are discussed as possible biocontrol agents (Newcombe, 2011; Witzell et al., 2014; Witzell and Martín, 2018). In addition, the ash dieback epidemic may deprive the resident endophytes of their niche and lead to extinctions of host specialized organisms. One example is the native sister species of the ash dieback pathogen, *H. albidus*, which has become rare or possibly extinct in some severely diseased areas (McKinney et al., 2012; Dvorak et al., 2015; Koukol et al., 2016).

Another major threat for all European ash species and consequently also their associated organisms is the emerald ash borer (*Agrilus planipennis*) (Musolin et al., 2017; Valenta et al., 2017). Originating in East Asia like *H. fraxineus*, the pest was introduced to North America, where it causes significant damage to ash trees. In 2002/2003, it has been has been detected in Russia, from where it is since spreading and will possibly arrive to Central Europe within two decades (Valenta et al., 2017).

The advent of NGS sequencing technologies and their utilization for microbial diversity analyzes enables studying the endophytic mycobiome at unprecedented precision. While there is a lot of research in the field of agroecology (e.g., Toju et al., 2018), the structure and functions of tree associated microbiota remain comparably understudied, despite of growing evidence for the importance of tree-fungus interactions (Busby et al., 2016; Witzell and Martín, 2018). Large-scale variation of ash mycobiota and interactions with ash dieback has not been thoroughly studied yet. Regarding the high-throughput analysis methods themselves, there is active research about potential biases and how precision can be improved (Hugerth and Andersson, 2017). The choice of primers is thereby very important as it has a large impact on the taxa found in the analysis (Kohout et al., 2014; Tedersoo et al., 2015; Tedersoo and Lindahl, 2016). For the study of host-associated fungi, there is only a limited set of high-coverage primers available, all of which have both strengths and limitations (Ihrmark et al., 2012; Toju et al., 2012; Bokulich and Mills, 2013; Tedersoo et al., 2015; Taylor et al., 2016).

The aim of this study was (i) to examine the geographic variability of the ash and sycamore maple leaf mycobiome, (ii) to provide a basis for evaluation of the ecological consequences of ash dieback on endophytes, and (iii) to find endophytes potentially involved in the protection of the host against ash dieback.

Fungal leaf communities of the European ash and sycamore maple (*Acer pseudoplatanus* L.) were examined on eight study sites in Switzerland and Northern Italy by using both cultivation and next-generation amplicon sequencing. At time of sampling (2013), the disease had been present in Switzerland north of the Alps for at least 5 years, while the southern region was still considered disease-free. Sycamore maple was included in the study because this species often occurs together with *F. excelsior* due to similar ecological preferences (Okali, 1966). A comparison of endophytic leaf mycobiota between the tree species should reveal insights into host specificity and consequently also the potential of these fungi to survive on *A. pseudoplatanus*. Since the tree is not affected by an epidemic on either side of the Alps, it was also regarded as suitable for estimating the influence of the geographic origin. Another potential refuge for *F. excelsior* endophytes is the flowering ash (*Fraxinus ornus*), which occurs natively in the southernmost part of Switzerland and is tolerant to ash dieback (Kirisits et al., 2009; Kirisits and Schwanda, 2015; Nielsen et al., 2017). Fungal leaf endophytes of *F. ornus* were studied by Ibrahim et al. (2017). Part of the samples from this study were additionally characterized by NGS amplicon sequencing. To address the third objective of this study, leaf endophyte communities from trees with visible symptoms of ash dieback were compared with those from healthy-looking trees on the North side of the Alps. Analyzes were done using a newly designed primer with high taxonomic coverage, which was previously validated both *in silico* and by sequencing of a test sample. Performance and possible uses of this primer and a related variant are discussed.

## Materials and Methods

### Sites and Sample Collection

**FIGURE 1:**
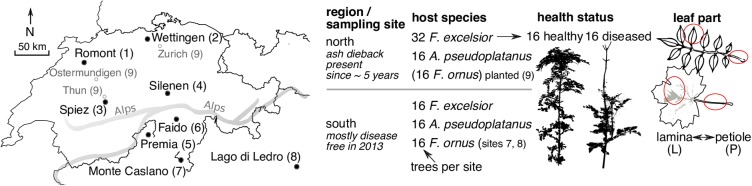
Map of sampling sites (left) and overview of variables examined in this study (right). The names and site numbers in the map are shown according to Supplementary Table S1 in Data Sheet 1 (filled circles). The empty circles with a gray font denote the locations of planted *F. ornus* trees north of the Alps sampled by Ibrahim et al. (2017). They were collectively assigned to number 9. The thick gray line illustrates the rough course of the main chain of the Alps.

Samples were collected at eight different mixed broadleaf forest sites, four being located north of the Alps and four in the south (Figure 1 and Supplementary Table S1 in Data Sheet 1). At all sites, *F. excelsior* and *A. pseudoplatanus* trees of 2–4 m height were sampled. The plots were grouped into four subplots of 10–25 m diameter, which contained four trees of each sampled species. The distances between subplots varied from 0–100 m depending on the sampling site, with the exception of the two southernmost sites in the native range of *F. ornus*. For site 7 “Monte Caslano” (Supplementary Table S1), the distances between subplots were up to 250 m, and for site 8 “Lago di Ledro,” they were up to 1.9 km since suitable sites containing all three species were difficult to find. The endophytic flora of *F. ornus* at these sites has been examined by isolation on agar plates from leaves collected at the same dates (Ibrahim et al., 2017). The sites 1, 2, 7, and 8 are located on relatively dry slopes in calcareous areas, whereas the sites 3–6 are located near riversides. Leaf collections were done between 2013-08-26 and 2013-09-04 (Supplementary Table S1). At each site, four healthy-looking leaves without symptoms were collected randomly from 16 trees of each host species from between 1.5 and 2.5 m above ground. On the north side of the Alps, 32 *F. excelsior* trees were sampled at each site. Sixteen trees with and 16 trees without symptoms of ash dieback were selected and apparently healthy leaves collected from all 32 trees. It was not always easy to find healthy looking trees; therefore on site 3, only 12 symptomless trees could be sampled. The collected leaves were stored separately (ziplock bags) for 6–12 h in a cool box containing ice packs, except for the Lago di Ledro site (8), where transport time was 48 h. Upon arrival in the laboratory, leaves were stored at 4°C and surface sterilization was done within 1 day.

### Processing of the Leaf Samples and Fungal Identification

From each of the four leaves collected per *Fraxinus* tree, one symptomless leaflet was selected randomly and surface sterilized along with the petiole. Petioles were cut at the lowest leaflet pair node (no rachis included). For *A. pseudoplatanus*, a leaf lobe was randomly selected if the leaf was too big to be sterilized as a whole. Petioles were cut at the base of the leaf blade. Surface sterilization was done using bleach (NaOCl) as follows: 1 min immersion in 70% ethanol, 3 min in NaOCl (4% active chlorine), 1 min in sterile water and 30 s in 70% ethanol. The effectiveness had been previously tested by a semiquantitative PCR analysis of fungal amounts wiped from the leaflet surface using cotton swabs. After immersion for 1-3 min. in NaOCl, almost no DNA was detected in the swabs anymore, while fungal DNA was still amplified from leaf tissue DNA extractions (M. Schlegel, unpublished). Five leaf disks of 7 mm diameter were cut from each of the four leaflets/the *A. pseudoplatanus* leaf lamina with a flame-sterilized puncher and collected into an Eppendorf tube, totaling to 20 leaf disks per tree. Similarly, a 5-mm long segment was excised from the central part of each petiole and collected into a tube, totaling to 4 petiole segments per tree. It was again taken care to only excise pieces from symptomless tissue. The samples were immediately frozen and stored at -80°C until DNA extraction. Additionally, two leaf disks and two petiole segments per tree were placed on a polycarbonate Petri dish (9 cm diameter) containing terramycine-malt-extract agar (TMA, 20 gl^-1^ malt extract, 15 gl^-1^ agar, 50 mgl^-1^ terramycine/oxytetracycline). The 312 Petri dishes for *F. excelsior* and *A. pseudoplatanus* were incubated at 20°C with regular inspection. Emerging mycelia were regularly transferred to slants containing malt-extract agar (MA, 20 gl^-1^ malt extract, 15 gl^-1^ agar) and fungal cultures were assigned to morphotypes based on growth rate, colony color and texture of the aerial mycelium. Sporulating cultures were identified based on literature as described by Ibrahim et al. (2017). Representatives of most morphotypes were further identified by sequencing of the ITS region according to the protocol used by Ibrahim et al. (2017). The sequences were assembled and aligned in Geneious (Biomatters, Auckland, New Zealand). All unique sequences were identified by BLAST against the NCBI *nt* database^1^. Sequences from reliable sources (culture collections, phylogenetic publications) were searched among the best hits, and taxonomic names were determined after inspection of alignments and phylogenetic trees produced with FastTree (Price et al., 2010). Species assignments were done if possible (for details see Supplementary Data Sheet 2). The sequences were uploaded to GenBank (accessions in Supplementary Data Sheet 2). The morphotype counts were visualized in R (R Core Team, 2017) for each host species, and community differences between hosts and geographical locations were analyzed using non-metric multidimensional scaling (NMDS) using vegan (Oksanen et al., 2017).

### Primer Design and Testing

The ITS4f/ITS4f2 primers (Supplementary Figure S1 in Data Sheet 1) were designed to amplify the fungal ITS2 region based on visual inspection of alignments from GenBank and UNITE (Kõljalg et al., 2013). ITS2 was chosen over ITS1 because it has less length variability and does not suffer from the presence of introns in the flanking rDNA, as found for ITS1 in some species (Tedersoo et al., 2015). Fungal specificity is conferred by the last two bases at the 3′ end of the primers. In order to prevent degradation by the 3′ to 5′ exonuclease activity of proofreading polymerases, phosphorothioate internucleotide linkages were used (Supplementary Figure S1). The taxonomic coverage of different primers was calculated by using 5.8S and 28S (LSU) sequences downloaded from GenBank (Supplementary Methods 1.1 in Data Sheet 1). The nucleotide frequencies at the four critical 3′ residues of the ITS4f primer were assessed by using the UNITE + INSD dataset, which contains less misclassified and chimeric sequences (Supplementary Methods 1.2).

For primer validation, a sample from a pond sediment containing high microbial diversity was collected near Zurich (47°22′2.5″ N, 8°28′31.9″ E). The pond is surrounded by forests and crossed by a stream. The sample was collected 5 cm deep within the sediment at a water depth of 50 cm and stored at -20°C. Total DNA was extracted using the PowerSoil^®^ Kit (MoBio/Qiagen) and further purified using the OneStep^TM^ PCR Inhibitor Removal Kit (Zymo Research). For an additional validation of the primers, three mock communities composed of 24 species distributed across the fungal kingdom were assembled. One mixture with equal amounts of genomic DNA from all species, and two uneven mixtures with geometric abundance distributions were assembled. The uneven communities differed in species composition and the dilution factor (Supplementary Methods 1.3).

The pond sediment sample and the mock community mixtures were amplified using the ITS3_KYO2 forward primer (Toju et al., 2012) and the reverse primers ITS4f/ITS4f2 and ITS4 (White et al., 1990; only sediment sample). The primers were ordered at Sigma-Aldrich (Germany) with linker sequences and Nextera XT overhang adapters at 5′ according to Table 1. The samples were amplified in triplicate in a total volume of 25 μl with 2 μl DNA, 0.75 μl of forward and reverse primers (10 μM) added to a final concentration of 0.3 μM, 0.75 μl (3%) of DMSO, 12.5 μl of 2× KAPA HotStart Ready Mix (Kapa Biosystems) and 8.25 μl PCR grade water. Reaction conditions were as follows: Initial denaturation at 95°C for 3 min, followed by 22 cycles of 98°C for 20 s, 50°C for 15 s, and 72°C for 25 s, and a final extension at 72°C for 5 min. The ITS4f2 primer was found to have a lower PCR efficiency, requiring more cycles (23 instead of 22). The triplicate reactions were pooled and purified with Ampure XP Beads (Beckman Coulter) by mixing 52.5 μl beads with 70 μl PCR product (0.75:1). Indexing and sequencing was done together with the leaf endophyte samples by using the methods described in the following chapter. The mock community amplicon was sequenced as part of a different library. The purified products of the sediment sample were sequenced in triplicate, while three replicates of the mock communities were independently mixed and amplified.

**Table 1:** Fusion primers used in the primer test and for amplification of the leaf samples.

Direction	Primer name	Nextera XT adapter	Shift^1^	Linker^2^	Primer sequence
Forward	ITS3-KOY2	TCGTCGGCAGCGTC	N{0–3}	GG	GATGAAGAACGYAGYRAA
Reverse	ITS4f	GTCTCGTGGGCTCGG	N{0–3}	GA	CGCTTATTRATATGCTTAA^∗^G^∗^T
	ITS4f2	GTCTCGTGGGCTCGG	N{0–3}	GA	CGCTTATTRATATGCTTAAR^∗^T
	ITS4	GTCTCGTGGGCTCGG		AA	TCCTCCGCTTATTGATATGC


*^*1*^Four different primers containing 0–3 frameshift nucleotides were mixed at equal molar concentrations. ^*2*^Chosen to have a low prevalence among fungi (Supplementary Methods 1.4). ^∗^Phosphorothioate linkage between nucleotides.*

**FIGURE 2:**
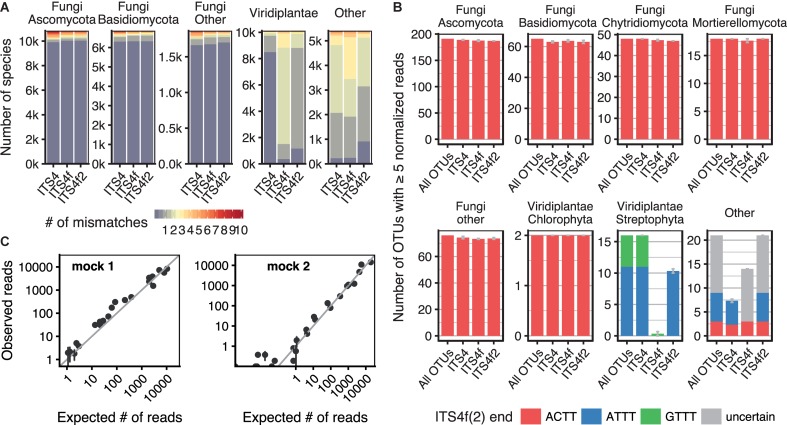
Validation of the new fungal-specific primers ITS4f/ITS4f2. **(A)** Overview of the taxonomic coverage of the two primers in comparison with ITS4 (White et al., 1990), determined using LSU sequences from GenBank. The bars indicate the number of species (GenBank classification) in thousands (k) and are colored according to the median number of primer mismatches per species. **(B)** OTU diversity in the pond sediment test sample, determined by NGS amplicon sequencing. The number of OTUs amplified by different reverse primers is shown for different taxonomic groups. The leftmost bars of each panel indicate the total OTU diversity retrieved by any of the primers (selected to be present in ≥3 samples with ≥10 normalized reads). For individual primer combinations, OTUs from this pool were considered ‘present’ if represented by ≥5 reads (±SEM from three replicates). The bars are colored according to the sequence variant found at the 3′ end of the ITS4f/ITS4f2 annealing site. Some OTUs did not have enough reads to unambiguously determine the sequence variant (‘uncertain’). **(C)** Expected vs. observed read numbers for each species in the uneven mock communities, amplified with the primers ITS3-KYO2 and ITS4f. The expected frequencies are corrected by qPCR quantification of rDNA content. The vertical error bars denote the SEM of three replicates, which were mixed and amplified independently.

Read processing, OTU clustering and taxonomic annotation of the sequences was done as described below for the leaf samples. The read numbers were scaled to the size of the sample with the lowest number of fungal reads (33,943 for the mock communities, 34,420 for the sediment sample). However, only reads without known mismatches to any of the tested primers were taken into account for calculation of the scaling factors. For the sediment sample, mismatches were determined based on the amplicon of the ITS4 primer. This was only possible for the last 6 bp before the 3′ end of ITS4f/ITS4f2, which does not overlap with ITS4 (see also Supplementary Figure S1). However, mismatches at the 3′ end of primers are also the most selective ones (Zhang and Li, 2003). A comparison of the OTU diversity captured by the different primers (Figure 2B) was done by defining a subset of ‘core’ OTUs present in ≥3 samples with ≥10 normalized reads. Since there were three replicates per primer combination, OTUs captured by one combination only would not be filtered out. For individual samples, OTUs were considered to be present if they had at least ≥5 normalized reads. Choosing a ‘core’ OTU set was done since direct filtering using a single threshold is sensitive to small frequency variations. A statistical comparison of fungal OTU numbers captured by each primer was done using a one-way ANOVA. The OTU sequences matching the four terminal ITS4f/ITS4f2 residues (Figure 2B) were validated by examination of the reads mapping to the OTU sequence. The most frequent sequence variant was considered to be the true OTU sequence if its frequency differed significantly from the frequency expected to occur by random (4^-4^, Fisher’s exact test). Otherwise, it was considered to be uncertain. OTUs with mismatches were manually reviewed using BLAST against the NCBI *nt* database in order to eliminate incorrect classification of non-fungal sequences as fungal (Supplementary Table File 2).

### DNA Extraction, Library Preparation, and Illumina Sequencing

Lamina and petiole DNA was extracted from half (eight) of the sixteen trees per site/host/health status combination. Extraction was done using the NucleoSpin^®^ Plant II Kit (Macherey-Nagel, Duüren, Germany). Two sterile metal beads (4 mm diameter) and 0.1 g of sterile sea sand were added into the 2 ml Eppendorf tubes containing the frozen leaf tissue. The samples were ground in a bead-beating mill at a frequency of 30 s^-1^ for 2 min (leaf lamina) or 4 min (petioles) while still frozen. Grinding was repeated until a homogenous powder was obtained. 500 μl of lysis buffer (PL1) containing 10 μl RNase A was added immediately. The buffer volume for petioles was 300 μl. Furthermore, 10% (v/v) of Polyvinylpyrrolidone (PVP, 0.1 g/ml) and 10% (v/v) of 2-Mercaptoethanol (≥99%) were added to the buffer. 2-Mercaptoethanol had been found to improve the extraction quality in preliminary tests. The samples were incubated for 45 min at 65°C. After clarification of the lysate an equal volume of chloroform/isoamyl alcohol (24:1) was added. The mixture was vortexed and centrifuged at 13,000 g for 3 min. The aqueous phase was transferred into a new tube and mixed with 450 μl of binding buffer (PC). Further steps were carried out according to the manufacturer’s instructions. DNA concentrations were measured using the Qubit dsDNA BR Assay (Thermo Fisher Scientific) and DNA was stored at -20°C.

Amplification was done using the primers ITS3-KYO2 and the newly designed ITS4f (Table 1). Samples were amplified in triplicate in a total volume of 15 μl with 1.5 μl DNA, 0.6 μl of forward and reverse primers (10 μM) added to a final concentration of 0.4 μM, 0.45 μl (3%) of DMSO, 7.5 μl of 2× KAPA HotStart Ready Mix (Kapa Biosystems) and 4.35 μl PCR grade water. Reaction conditions were as follows: Initial denaturation at 95°C for 3 min, followed by cycles of 98°C for 20 s, 52°C for 15 s, and 72°C for 25 s, and a final extension at 72°C for 5 min. The number of cycles needed for obtaining sufficient PCR product had previously been determined by a qPCR assay using the same primers and 20× EvaGreen^®^ Dye (Biotinum), performed on a LightCycler^®^ 480 Instrument II (Roche, Basel, Switzerland) in a total volume of 5 μl. Most samples were amplified using 19, 24, or 29 cycles while 16 petiole samples were amplified using 33 cycles. Additionally, the even and the first uneven (uneven_1) mock communities (previous chapter) were amplified along with each cycle group for validation of the bioinformatic analysis. They were diluted based on the qPCR results to have fungal gDNA concentrations comparable to those of the samples within each group (Supplementary Table S7 in Data Sheet 1). Only one mixing replicate per community was used. The triplicate reactions were pooled after amplification and 42 μl of the pool were purified using custom-made SPRI beads (OpenWetWare, 2017). 35.7 μl of beads were added (0.85:1) and washed twice with 80% EtOH according to the protocol used for Ampure XP bead purifications (Beckman Coulter). DNA was eluted in 15 μl of 5 mM Tris/HCl buffer. Indexing was performed using 6 μl of purified PCR product in 25 μl reactions containing 2.5 μl of both Nextera XT Index Primer (N7xx, S5xx), 0.75 μl (3%) DMSO, 12.5 μl of 2× KAPA HiFi HotStart Ready Mix and 0.75 μl PCR grade water. Thermocycling conditions were 95°C for 3 min, followed by 10 cycles of 95°C for 30 s, 55°C for 30 s, and 72°C for 30 s, and a final extension at 72°C for 5 min. 24 μl of indexed product was purified using 21.6 μl SPRI beads (0.9:1). The libraries were quantified, pooled and sequenced on an Illumina MiSeq using the reagent kit v3 (600 cycles, 2 × 300 bp) (Illumina, Inc., Carlsbad, CA, United States). Two sequencing runs with reduced cluster densities were done in order to obtain enough high-quality reads. For the second run, samples with low read numbers were spiked with more DNA in order to obtain more reads. However, for the analysis, samples with a high difference in number of reads between the runs were rarefied to a lower level (randomly) before analysis in order to avoid introducing a bias resulting from the two runs not generating completely comparable results.

### Bioinformatic Analysis

Paired end reads were merged using USEARCH (Edgar and Flyvbjerg, 2015) with at most 30 or 15% mismatches between the reads allowed. Subsequent error filtering was done with VSEARCH 2.7 (Rognes et al., 2016) with a strict maximum expected error rate of 0.002 (allowing 0.6 errors for a 300 bp amplicon on average). Primer sequences were searched and removed with seqtool^2^, a program written by the author (M. Schlegel). The sequences were clustered using the UNOISE algorithm of USEARCH (Edgar, 2016c). Since clustering combined *H. fraxineus* and *H. albidus* into one OTU, a reference sequence for *H. albidus* was manually added. The OTU table was constructed by mapping the unfiltered reads against the OTUs with VSEARCH. Taxonomic identification was done with SINTAX implemented in USEARCH (Edgar, 2016a) with a reference database composed of the fungal USEARCH/UTAX reference dataset from UNITE (2017-10-10) (Kõljalg et al., 2013), ITS2 sequences of the host tree species and a custom dataset of non-fungal sequences downloaded from GenBank and clustered at a 70% threshold (Supplementary Methods 1.5 in Data Sheet 1). The ITS2 region was identified and extracted by using ITSx (Bengtsson-Palme et al., 2013). Additionally, all OTUs were compared with the taxonomic reference database using nucleotide BLAST (Camacho et al., 2009). Sequences with unclear taxonomic annotation (classified as fungal, but without more precise taxonomic annotation; SINTAX cut-off: 0.8) were treated as unspecific if the *E*-value was above 0.01 or the query coverage lower than 0.2. The classification of abundant OTUs was additionally verified by manual examination of the BLAST hits and comparison with published phylogenies, as done for the morphotypes.

### Statistical Analyzes

All analyzes were done in R (R Core Team, 2017) using the *phyloseq* package, v1.20.0 and *ggplot2* for the figures. The Shannon alpha diversity measure was calculated from all fungal OTUs (including singletons) with the exception of *H. fraxineus*. Differences in alpha diversity between the north/south side of the Alps were analyzed using a linear mixed model implemented in the *lme4* package (Bates et al., 2014). Sampling site and the number of PCR cycles were both included as random intercept. The comparison was done separately for each host species and leaf part. For all other analyzes, only OTUs present in at least four samples with at least 10 reads each were used. In order to deal with the compositional nature of the data (Gloor et al., 2017), a centered log ratio transformation (CLR) was applied before further analyzes. For analyzes comparing different taxon abundances and most figures showing absolute OTU abundances, read numbers were scaled to a total of 20,000 reads per sample (hereinafter referred to as “scaled reads”). Samples with less than 16,000 reads were removed.

The rate of reads assigned to incorrect samples (crosstalk; see Edgar, 2016b) was quantified using the control samples. The approximate maximum number of misassigned sequences possible at a certain OTU size was modeled using quantile regression (*rq* function from the quantreg R package, tau = 0.995). Since crosstalk can also be visible on graphs showing log-transformed read abundances, counts likely to be derived from crosstalk were set to zero in order to improve the readability for some graphs. Statistical analyzes were always performed with uncorrected counts.

Overall differences in community structure were analyzed by unconstrained ordination. A principal component analysis (PCA) of the CLR transformed data was done, which is based on Euclidean distances. To test for community differences between host species, sites/regions and healthy vs. diseased trees, permutational analysis of variance (PERMANOVA) was done as implemented in the adonis2 function of the *vegan* package v2.4.5 (Oksanen et al., 2017). All tests were run separately for lamina and petiole communities using 99,999 permutations, and the amplification group (number of PCR cycles) was always included as factor. The comparisons of regions (north and south of the Alps) and tree health were run separately for the different host species and both leaf parts. For the exact models see Supplementary Methods 1.8. The resulting *p*-values were corrected for multiple testing using the Benjamini and Hochberg method (*p.adjust* function).

The *ALDEx2* R package (Fernandes et al., 2014) was used for finding individual taxa associated with the different host species, regions and healthy or diseased trees. This involved the generation of 256 Monte Carlo (MC) instances from CLR transformed read counts. Only taxa with a high prevalence (>15 reads in >6 samples) were included to increase statistical power (Bourgon et al., 2010). A linear mixed model implemented in the *lme4* package (Bates et al., 2014) was fitted for each OTU with one of the mentioned factors specified as fixed effect. The sampling site (if not the main factor) and the number of PCR cycles were included as random effects. For details see Supplementary Methods 1.9. *P*-values were obtained by a type II Wald chi-square test and summarized by their mean value over all MC instances. The *p*-values were adjusted using the Benjamini and Hochberg method. OTUs below a false discovery rate (FDR) of 0.1 were reported as significant. An exception are the comparisons of the regions north/south of the alps and symptomatic vs. asymptomatic trees. There, OTUs with a significant *p*-value (<0.05) before FDR adjustment are presented. This seemed justified since the statistical procedure used for differential abundance testing was found to be quite conservative compared to other analyzes that had been evaluated (DESeq2, edgeR; data not shown).

A possible effect of tree health on the abundance of *H. fraxineus* was examined using a linear mixed model (lme4) including leaf part and tree health and their interaction as fixed effects and the sampling site as random intercept, and the scaled and log10(× + 1) transformed read abundances as response variable. The *p*-values were calculated using a Wald chi-square test. The association of *H. fraxineus* levels in petioles vs. laminae was tested using a linear regression analysis with scaled and log-transformed read abundances.

Negative or positive abundance relationships with *H. fraxineus* in *F. excelsior* were analyzed using SPARCC (Friedman and Alm, 2012). The correlations were calculated separately for lamina and petiole communities, but sampling sites were not distinguished. Pseudo *p*-values were obtained after 1000 bootstrap simulations. For significant (pseudo *p* < 0.05) candidates, the association was additionally confirmed using a linear mixed model (lme4) testing the log-transformed read abundance of each fungus in response to the log-transformed abundance of *H. fraxineus*, including the sampling sites and the number of PCR cycles as random intercepts.

## Results

### Validation of New Primers

Two newly designed fungal specific primers named ITS4f and ITS4f2 were validated *in silico* using LSU sequences available on GenBank. The number of fungal species with mismatching positions to these primers was low, even slightly lower than for the ITS4 primer (Figure 2A). The selectivity of the ITS4f/ITS4f2 primers relies on the last two 3′ bases, which differs from most land plant sequences (Supplementary Figure S1 in Data Sheet 1). Due to the possibly detrimental effect of terminal mismatches on the amplification (Kwok et al., 1990; Huang et al., 1992; Zhang and Li, 2003; Wu et al., 2009), prevalence of the four terminal ITS4f residues in UNITE database was examined separately. A few fungal taxa were found to differ from the ITS4f primer in their 3′ sequence whereas they were matched by the ITS4f2 primer due to its additional degeneration (see Supplementary Table S5 in Data Sheet 1). This includes some basal lineages (Kickxellomycota and Zoopagomycota, all Entomophthorales, GS19; Tedersoo et al., 2017) and Peltigeraceae (lichen-forming fungi), a rare *Mortierella* sp. and eventually other species with uncertain classification and sequence quality. Other taxa are not matched by both primers: Microsporidia (all), representatives of *Tulasnella* spp. and *Xylaria cubensis*. Other Xylariales, which are frequent tree endophytes, are amplified by the primers. The two selective positions at the ITS4f 3′ end were very conserved in the fungal kingdom, but less conserved within plants (Supplementary Figure S2). The sequence seems invariant within whole plant orders in some cases, but in other cases variation between different species of the same genus was found. Detailed information about the taxonomic sequence distribution at these positions is given in Supplementary Table File 3.

The primers were additionally validated using a highly diverse pond sediment sample with low plant content and mock communities assembled from 24 fungal species. The universal primer ITS3-KYO2 (Toju et al., 2012) was chosen as forward primer as it amplifies more fungal taxa than the often used primers fITS7 and fITS9 (Ihrmark et al., 2012; Supplementary Figure S3). The number of fungal OTUs amplified from the pond sediment sample by ITS4f/ITS4f2 and ITS4 was very similar (Figure 2B; ANOVA *p* = 0.137). Only few possibly fungal OTUs were affected by mismatches to ITS4f, but not ITS4f2; however BLAST did not allow a clear assignment to a specific kingdom (Supplementary Table File 2). The ITS4f primer was very effective in reducing plant amplicon from this sample, whereas the ITS4f2 primer was slightly less selective toward plants and allowed the amplification of more organisms as expected due to the additional degeneration (Figure 2B and Supplementary Table S6). Amplicon sequencing of a *F. excelsior* leaf sample confirmed the strong selectivity of ITS4f (0.7% host amplicon), while ITS4f2 amplified 34.5% host DNA (data not shown), suggesting that this primer was not discriminatory enough for our analyzes of leaf mycobiota. In comparison, amplification of the leaf samples of this study with ITS4f revealed a slightly higher, but still acceptably low amount of plant reads. Only very few samples had a higher plant content of 20–30% or up to 75% in rare and extreme cases (Supplementary Figure S8).

Species abundances in the mock communities corresponded well with the expected read counts for the ITS3-KYO2 – ITS4f amplicon (Figure 2C). The estimation of ribosomal DNA (rDNA) content for each of the 24 species by qPCR further improved the prediction, as indicated by the correlation with their read abundances in the even mixture (Supplementary Figure S4).

### Endophytic Mycobiota of Ash and Sycamore Leaves

Leaves from trees of the European ash (*F. excelsior*) and sycamore maple (*A. pseudoplatanus*) were sampled at four sites north of the Alps and four sites on the south side (Figure 1). In the area infested by ash dieback north of the Alps, an equal number of *F. excelsior* trees with and without dieback symptoms were sampled per site. The endophytic fungal communities were analyzed by traditional isolation of surface sterilized lamina and petiole pieces. For half of the trees, community structure was further analyzed using NGS amplicon sequencing. Leaf samples of the flowering ash (*F. ornus*) collected by Ibrahim et al. (2017) were also included in this analysis. They had been collected at two sites located in the native range of the species (7 and 8 in Figure 1) and from planted trees north of the Alps (9). The latter were treated as a single “site” for the analyzes even if the trees were collected from four locations.

All samples were sequenced as part of one Illumina library in two consecutive runs. For 96% of the samples, total read numbers were within the range of 27,690–69,599 sequences (median: 48,770; Supplementary Results 2.1 in Data Sheet 1). Due to the large differences in initial amounts of fungal DNA inherent to real-world environmental samples, samples had to be grouped and amplified using different cycle numbers (19, 24, 29, and 33) in the first amplification step. The even and the first uneven mock community mixture (chapter 4.1) were used for quality control. All mixed species were present as expected and the number of singleton OTUs and chimeric reads was low (Supplementary Results 2.2). However, more PCR cycles in the first amplification step lead to less rare species being found. Since a majority of lamina samples were amplified with 19 or 24 cycles and most petiole samples with 29 cycles (Supplementary Figure S7C), most analyzes were done separately for both leaf parts, and the number of PCR cycles was included in all statistical models if possible. There was also a low amount of reads assigned to incorrect samples, commonly referred to as crosstalk (Edgar, 2016b; Supplementary Results 2.3). We also identified contaminating DNA from a few fungal species, which had been accidentally introduced during DNA extraction of some samples. The taxa were removed from the dataset (Supplementary Results 2.2).

**FIGURE 3:**
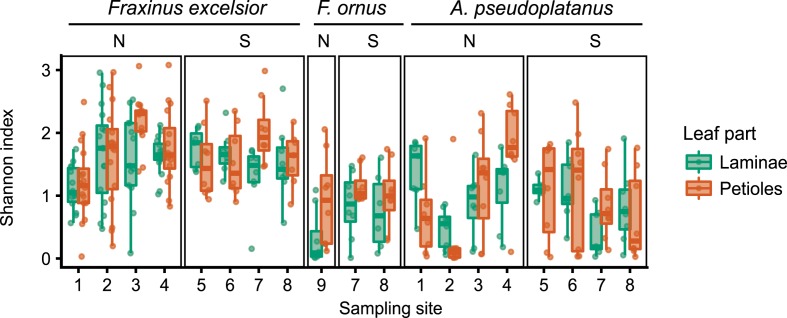
Variability of the Shannon alpha diversity measure, summarized per sampling site for all host species and both leaf parts (green, laminae; orange, petioles). The overlaid points represent individual tree samples. The sites are grouped into the north (N) and south (S) side of the Alps. “Site” 9 collectively refers to all planted trees sampled on the north side of the Alps.

Clustering of the amplicon sequences yielded 2251 OTUs, from which 1562 had at least 20 reads. A majority of the OTUs (1090) was only found in one single lamina or petiole sample, and only 55 OTUs were present in more than 10% of all samples (Supplementary Figure S11). The Shannon alpha diversity index varied between different sites and leaf organs, although with no clear pattern. There is no indication that diversity was higher in the almost disease free region south of the Alps (Figure 3 and Supplementary Table S8). Only for *F. ornus* laminae, there was a trend toward a lower diversity on the north side of the Alps (PERMANOVA *p* = 0.027 without adjustment for multiple testing) even if planted trees from four different locations were combined in the analysis (“site” 9 in Figure 1). The Shannon index also showed a dependency on the number of PCR cycles (Supplementary Figure S12).

**FIGURE 4:**
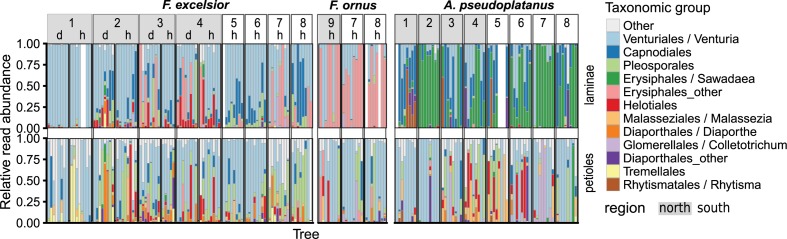
Relative abundances of the most frequently encountered fungal taxonomic groups, summarized at the order level. If >75% of all reads in an order belonged to a specific genus, then this genus was used as group instead and the remaining OTUs were classified as “Other.” The frequencies are shown for each host species and leaf part (vertical panels) for different sampling sites (1–9) and health status (h, healthy; d, diseased).

In terms of read abundance, the dominant groups were often *Venturia* spp. or powdery mildew species (Erysiphales), but their occurrence varied between sampling sites, host species and leaf parts (Figure 4).

### Comparison of Classical Cultivation and Amplicon Sequencing

**FIGURE 5:**
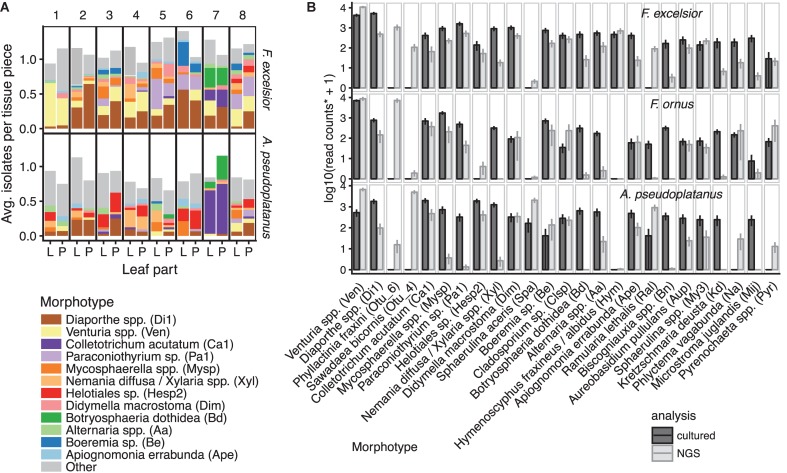
Overview of morphotype abundances. **(A)** Mean isolation frequencies of the most abundant morphotypes on *F. excelsior* and *A. pseudoplatanus* from lamina (L) and petioles (P) per sampling site (number 1–8). Frequencies above one indicate that more than one fungus emerged from one lamina/petiole piece on average. **(B)** Mean abundances of the most frequent taxa obtained by the culturing method or NGS sequencing. OTUs were considered belonging to a cultured morphotype if they had at least 97% similarity to a sequenced isolate from that morphotype. Additionally, the two most abundant OTUs not found by culture are shown. The morphotype codes are written in brackets. Potentially misassigned sequences (crosstalk) were removed from the NGS dataset. ^∗^In order to be comparable with the NGS read counts, the mean relative isolation frequencies from all samples (laminae + petioles) scaled to the average read depth of the scaled NGS samples (20,000). Error bars represent the standard error of the mean (SEM). For isolates from *F. ornus*, data from Ibrahim et al. (2017) are shown.

Classical isolation of endophytic fungi from surface-sterilized leaf tissues of *F. excelsior* and *A. pseudoplatanus* yielded 56 abundant morphotypes and around 29 additional rare morphotypes that occurred only once. A total of 1,252 fungi that emerged from 1,280 leaf segments were examined. A majority of 38 morphotypes were shared between the two hosts, while 35 of all morphotypes were shared with the ones defined by Ibrahim et al. (2017) for *F. ornus*. Isolates from most morphotypes were further characterized by sequencing of the ITS region. The sequence data from Ibrahim et al. (2017) were re-analyzed in combination with the sequences from this study. From a total of 224 sequences belonging to 84 morphotypes, 141 unique sequences (‘clones’) were found, belonging to 96 putative species (Supplementary Data Sheet 2). The frequency of the morphotypes showed a hyperbolic distribution with a few very abundant and many rarely occurring morphotypes, a distribution typical for endophyte communities. On *F. excelsior*, *Venturia fraxini* was the most isolated species, similarly to *Venturia orni* on *F. ornus* (Ibrahim et al., 2016). Others like *Colletotrichum acutatum*, *Botryosphaeria dothidea*, and *Diaporthe* spp. were dominant on some sampling sites only (Figure 5A). An ordination analysis confirmed differences between sites, whereas lamina and petiole communities often clustered together (Supplementary Figure S10).

Almost all cultivated morphotypes with ITS sequences available were also found by NGS sequencing, although the relative frequencies differed and were not always consistent between hosts (Figure 5B, see Supplementary Data Sheet 3 for a comprehensive comparison). Some species were only found by NGS, including the powdery mildew species *Phyllactinia fraxini* and *Sawadea bicornis* and the tar spot-causing fungus *Rhytisma acerinum* on sycamore, which are known obligate biotrophs. In petiole samples, basidiomycetous yeasts of the Tremellales and Malasseziales and others were only found by NGS (Supplementary Data Sheet 3). On the other hand, fast-growing members of the Xylariales, *Botryosphaeria dothidea* and others were often isolated, but rarely found in the NGS analysis. Only few rather rare fungi were found by culture, but not by NGS. This includes *Annulohypoxylon* spp. (Xylariales), which was isolated 10 times in total, but never amplified, even if BLAST suggests no mismatches to the amplicon primers. *H. fraxineus* was isolated 16 times from symptomless *F. excelsior* leaves.

### High Geographic Variability

**FIGURE 6:**
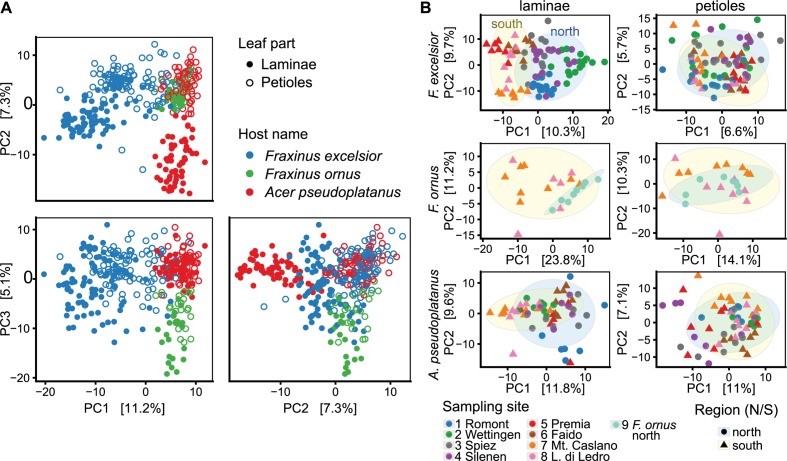
Unconstrained ordination using principle component analysis (PCA) of CLR-transformed OTU counts. The percentages explained by each axis are shown in square brackets. *H. fraxineus* was excluded from this analysis. **(A)** Ordination including all samples, colored according to the host species. Filled circles indicate lamina (L) and open circles petiole samples (P). Combinations of the first three axes (PC1-3) are shown. **(B)** Ordination done separately for each host species and leaf part (PC1 and 2 shown). Tree communities north of the Alps are outlined with a blue elliptic background and samples from the south have a yellow background (confidence level: 0.9).

NGS amplicon sequencing revealed clear differences in endophytic community composition of the three hosts and different leaf tissues, as examined using unconstrained ordination (Figure 6A). The amount of variation explained by the three axes was rather low (23.7% in total), suggesting a high complexity of the dataset. Separate analyzes for each host and leaf part additionally revealed a strong geographical variation. Especially the lamina communities of one sampling site often clustered together (Figure 6B). The largest amount of variance of lamina communities was explained by host species (22%), followed by sampling site (14%; PERMANOVA *p* < 0.001 for both; Supplementary Table S9). In petiole communities, the explanatory power of both factors was lower (9%; *p* < 0.001).

**FIGURE 7:**
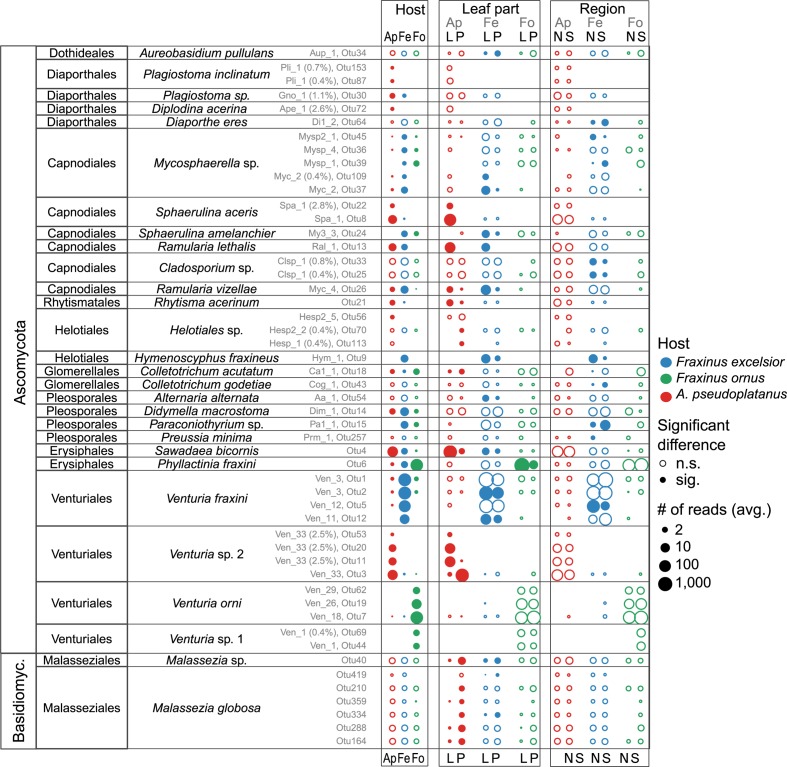
Overview of species for which significant differences between the host species (Ap, *A. pseudoplatanus*; Fe, *F. excelsior*; Fo, *F. ornus*), leaf parts (L, laminae; P, petioles) and/or between the north (N) and south (S) side of the Alps exist. The dots are colored according to the host species, and their size varies depending on the mean read abundances (average of sampling site averages). Potential artifacts (crosstalk) were removed before computing the averages. Filled circles indicate a significant difference for the given factor while empty circles indicate non-significant differences. Note that for the region (north/south), the significance was determined from un-adjusted *p*-values due to reasons described in the text. The OTU numbers are shown in gray together with the code of the cultured isolate (Supplementary Data Sheet 2) if present (and % deviation of OTU from isolate sequence). For more details see also Supplementary Figures S13–S18.

Principal component analysis also indicated a distinct, yet overlapping grouping of sites from the same side of the Alps for *F. excelsior* laminae (Figure 6B). This is interesting, since the south side was mostly disease-free. PERMANOVA confirmed a difference between the two regions for both lamina and petiole communities of *F. excelsior*, but not for communities of sycamore maple and the flowering ash (Supplementary Table S10). OTUs more abundant in the diseased area included a *Mycosphaerella* sp., two *Cladosporium* spp., *Preussia minima* and one *Venturia fraxini* genotype (Figure 7 and Supplementary Figures S17, S18). In contrast, *Paraconiothyrium* sp., *Colletotrichum godetiae* and another *Mycosphaerella* sp. were more abundant south of the Alps. *Venturia* sp. 1 discovered by Ibrahim et al. (2016) on *F. ornus* in its native distribution range was not found in planted trees north of the Alps at all.

### Host and Tissue Specificity

**FIGURE 8:**
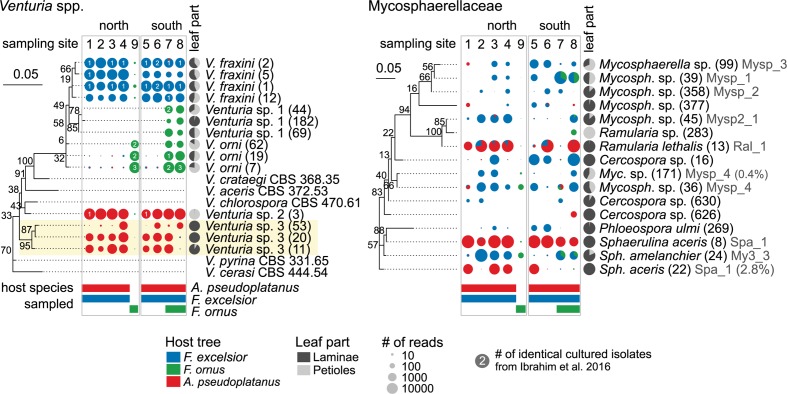
Host specificity of dominant leaf endophyte groups. Phylogenetic trees of *Venturia* spp. and Mycosphaerellaceae OTUs (numbers in brackets) and published sequences are shown along pie charts illustrating the abundance distribution of NGS reads between different host species. The OTU occurrence is shown for each sampling site on both sides of the Alps, scaled according to the average number of normalized reads. The host species that were actually sampled at these locations are indicated below with colored bars. The gray pie charts indicate the abundance distribution between laminae and petioles, averaged over all samples. The numbers on the pie charts indicate the number of *Venturia* isolates from Ibrahim et al. (2016). The isolate codes (Supplementary Data Sheet 2) are shown in gray (with % deviation of OTU from isolate sequence). OTUs from two new *Venturia* species on *A. pseudoplatanus* are highlighted by a yellow background. *V. fraxini* OTUs 1 and 2 matched the same isolate sequences because their sequences differed only near the 3′ end, which did not overlap with the isolate sequences. The trees include ML bootstrap percentages next to branches. The scale bars represent the number of substitutions per site.

Endophytes with a preference for a certain host species are expected to be most affected by reduced host densities. Therefore, OTUs with a differential distribution across hosts were identified. Among the 79 more abundant OTUs selected for the analysis 33 (42%) showed a more or less strong preference for one or two hosts (*p* < 0.05). In petioles, only 11 of 87 (13%) fungi were differentially distributed. The abundant representatives of the *Venturia* genus exhibited the strongest host preference (Figures 7, 8 and Supplementary Figures S13, S14), confirming the findings of Ibrahim et al. (2016). Several *V. fraxini* and *V. orni* OTUs were specific for their respective hosts, *F. excelsior* and *F. ornus* (Figure 8). The rare *Venturia* sp. 1, which had been found in *F. ornus* leaves from site 7 (Caslano) by isolation (Ibrahim et al., 2016) was detected by NGS at both southern sites with natural regeneration of the flowering ash, but not in planted trees north of the Alps. On *A. pseudoplatanus*, NGS confirmed the presence of *Venturia* sp. 2 (Ibrahim et al., 2016). It was found to be specifically located in petioles, while another group of OTUs specific for laminae (named *Venturia* sp. 3) was detected only by NGS. For both putative species, no close relatives were found in public databases. They are also distinct from the only known sequence of *V. aceris* (Crous et al., 2007), a known fungus in maple leaves (Sivanesan, 1977; Figure 8).

The Mycosphaerellaceae constitute another abundant and diverse group with host preferences. Most species were detected to varying extents in both *Fraxinus* hosts, but not or only at very low levels in *A. pseudoplatanus* samples (Figures 7, 8). While the amplification from *F. ornus* samples was sometimes low, most OTUs were detected by cultivation (Ibrahim et al., 2017; see Supplementary Data Sheet 2). *Sphaerulina aceris* was specific for sycamore maple, while *Ramularia lethalis* (another known *Acer* associate; Videira et al., 2016) was also amplified from *F. excelsior*.

As expected, the obligate biotrophic powdery mildew species (*Phyllactinia fraxini*, *Sawadea bicornis*) and *Rhytisma acerinum* were more abundantly, although not exclusively amplified in samples from their respective hosts. Additional sycamore maple associates, which had also been reported as specific for this tree by Unterseher et al. (2007) were *Plagiostoma inclinatum*, *Helotiales* sp., and *Diplodina acerina*.

Ordination analysis revealed a pronounced discrimination of *F. excelsior* lamina and petiole communities (Figure 6). The preference of the two possibly novel *Venturia* species on sycamore maple for petioles has been mentioned above. Several additional species especially from the Capnodiales with a significant preference for a host tree were found, which also showed a more or less strong preference for laminae or petioles (Figure 7 and Supplementary Figures S15, S16).

### The Ash Dieback Pathogen, Endophytes and Tree Health

**FIGURE 9:**
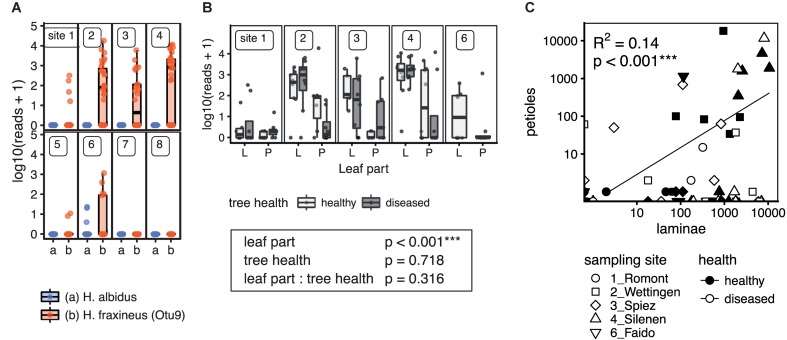
Detection of the ash dieback pathogen *H. fraxineus* and the closely related native *H. albidus* in European-ash leaves by NGS sequencing. **(A)** Distribution of *H. fraxineus* and *H. albidus* across the different sampling sites. Scaled and log transformed read counts are shown as boxplots overlaid with individual sample points. Potentially misassigned rare *H. fraxineus* sequences (crosstalk) were removed. **(B)** Amount of *H. fraxineus* reads in lamina and petiole samples depending on the tree health. Only sampling sites where the pathogen was present are included. In addition, the *p*-values (Wald Chi-square test) of a linear mixed model for the effect of leaf part and tree health are shown. Three stars (^∗∗∗^) indicate a significant effect. **(C)** *H. fraxineus* content in laminae vs. petioles. The regression line is shown along with the adjusted R-squared and *p*-value of the linear model. ^∗∗∗^Significant effect.

The ash dieback pathogen *H. fraxineus* was detected at high numbers north of the Alps as expected, but also on the Faido site (6) south of the Alps (Figure 9A). Necrotic lesions on twigs and apothecia of the pathogen could already be observed while collecting for this study, which lead to the first report of the species occurring south of the Alps in Switzerland (Sieber, 2014). Interestingly, infection levels on site 1 (Romont) located in the Jura Mountains were low. Petiole samples had generally lower relative levels of *H. fraxineus* DNA and the levels were correlated (Figure 9C). In contrast, the native endophyte *H. albidus* was only found at a very low frequency (max. 28 reads) in four lamina samples from the Faido site (6), the same site on which *H. fraxineus* was first observed south of the Alps.

*Hymenoscyphus fraxineus* is known to colonize the leaves of the tree before it proceeds through the petioles into the stem. Direct or indirect interactions with other leaf colonizers are thus possible. Therefore, OTUs with a positive or negative correlation of their frequencies with *H. fraxineus* were determined using SPARCC (Friedman and Alm, 2012). Only one species with a negative relationship was identified (*Setophoma* sp.), while a few species showed positive associations, including *Boeremia* sp. and *Phlyctema vagabunda* (Supplementary Figure S20).

A comparison of communities on *F. excelsior* trees with and without visible symptoms of ash dieback yielded no significant difference although there was a trend for lamina communities (PERMANOVA *p* = 0.084; Supplementary Table S11). Similarly, OTUs with a weak preference for (a)symptomatic trees were only found if the *p*-values were not FDR corrected (Supplementary Figure S19). From the OTUs with the strongest patterns, *Diaporthe rudis* and *Boeremia* sp. were more frequent in (healthy) laminae of symptomatic trees, while a *V. fraxini* OTU showed a trend toward a higher abundance in laminae of healthy trees. For *Boeremia* sp., a significant difference could be obtained if restricting the analysis to the two sites where it occurred (sites 2 and 3; not shown). Interestingly, the colonization frequencies of the ash dieback pathogen itself were not different in leaves of healthy or diseased trees (Figure 9B).

## Discussion

Distinctive endophytic mycobiomes associated with European ash (*F. excelsior*), manna ash (*F. ornus*), and sycamore maple (*Acer pseudoplatanus*) were found by both NGS (using newly developed primer combinations) and culturing. The communities were characterized by few abundant and host specific species, which is in line with previous findings on fungal tree endophytes (Sieber, 2007). A high geographic variability was found, as well as differences between the north and south side of the Alps for *F. excelsior*. Only minor community differences between asymptomatic trees and trees affected by ash dieback were detected.

### Methodological Considerations

The ribosomal DNA (rDNA) sequences flanking the ITS region are highly conserved across Eukaryotes, enabling the design of universal primer sets. However, sites conserved within the fungal kingdom also tend to be highly conserved across Eukaryotic kingdoms (e.g., Gutell et al., 1985; Schnare et al., 1996; Cheng et al., 2016), which poses challenges for the design of fungal-specific primers. A frequently used primer for sequencing of the ITS2 region is fITS7 (Ihrmark et al., 2012). Despite its high coverage, it also suffers from mismatches in some groups that might be found as leaf endophytes (Tedersoo et al., 2015). The primers used in the present study (ITS4f and ITS4f2) are located at a highly conserved site of the LSU rDNA overlapping with the ITS4 primer, which is used for many NGS amplicon studies due to its ability to amplify most fungi and other Eukaryota (Tedersoo and Lindahl, 2016). The specificity of ITS4f depends on the last two 3′ bases, which were protected from degradation using phosphorothioate linkages. ITS4f proved to be suitable for the analysis of fungal endophytic communities, exhibiting a strong specificity (see also Supplementary Discussion 3.1 in Data Sheet 1). ITS4f2 relies only on a single selective position at the 3′ end, leading to increased taxonomic coverage, but also reduced specificity. For this reason, the primer could not be used in this study, but it might be preferred if the specificity is sufficient. Especially for rhizosphere analyzes where more species from basal lineages may be encountered, there is a small risk for mismatches to ITS4f. A primer similar to ITS4f2 named ITS4-Fun has been developed independently by Taylor et al. (2016) (see Supplementary Table S5 in Data Sheet 1) and validated using a *Taq* polymerase. The primers ITS4f and ITS4f2 should be suitable for use with a wide range of hosts, but not all. Some guidance for primer selection is provided by the sequence summary in Supplementary Table File 3.

Interestingly, isolates of the ascomycete *Xylaria cubensis* originating from a wide geographical range were found to have an exceptional sequence within the LSU region around ITS4, resulting in mismatches to all primers binding there. It is possible that this species is more common than currently known and may have been missed by some amplicon studies (Supplementary Discussion 3.1). Both the culturing and NGS methods resulted in similar community patterns, confirming earlier findings on tree endophytes (Siddique et al., 2017). Species of the genus *Venturia* and the Mycosphaerellaceae were abundantly recovered by both methods. For other groups, the quantities of single taxon groups differed; especially the Xylariales were underrepresented in the NGS dataset. Since no mismatches to the primers were present, it is possible that they were in fact more often found by isolation due to their generally fast growth on culture media. On the other hand, obligate biotrophic fungi from the Erysiphales, *Rhytisma acerinum*, and several basidiomycetous yeasts specific to the petiole were not found by culture. The occurrence of *Malassezia* spp. reads was rather unexpected since these species are primarily known as skin colonizers, but in fact they appear to be cosmopolitan and to also occur in *F. excelsior* (Supplementary Discussion 3.2). NGS amplicon pipelines also suffer from many methodological biases, which currently make it impossible to compare abundances between different species (Hugerth and Andersson, 2017). In addition, the distinction between living and dead organisms is not possible (Emerson et al., 2017). Still, NGS sequencing provides a much more precise picture of diversity. Ideally, both methods are applied in combination.

The mock communities allowed for investigating the biases specifically introduced during amplification and sequencing. Rare species were not well recovered in samples amplified with many PCR cycles, which were the samples with low amounts of fungal DNA. It is likely that not enough template molecules from these rare species were present in the reaction (see Supplementary Discussion 3.3). We conclude that the use of mock community controls with staggered abundances including highly diluted species is very important for studies with low amounts of microbial DNA. The problem can be partly reduced by using large reaction volumes and many PCR replicates (Ficetola et al., 2015).

### Could Ash Endophytes Go Extinct?

A major question to be answered by this project was, whether ash dieback caused by *H. fraxineus* may lead to changes and in the native endophytic mycobiota, maybe even extinctions (aim ii). Co-extinction of affiliated organisms might be one of the most important reasons for the loss of species, and mutualists or parasites are possibly most affected (Dunn et al., 2009). There are two possible scenarios that could lead to a biodiversity loss: (i) The presence of *H. fraxineus* in leaves could directly influence the presence and life cycle of other leaf inhabiting fungi, either through competition in the leaf or through necrotic lesions, wilting and/or early leaf shedding; (ii) The reduction of host densities or the complete extinction of ash could indirectly lead to a loss of linked biodiversity. This concerns mostly host specific fungi lacking the possibility to survive on other hosts like the flowering ash (in southern areas in the case of Switzerland) or sycamore maple.

A possible example of direct influence is the replacement of the harmless native endophyte *H. albidus* by *H. fraxineus*, as reported by McKinney et al. (2012). The authors examined four sites in Denmark, where the fungus had been abundant prior to 2005. In 2010, only *H. fraxineus* was found. On the other hand, *H. albidus* was still detected in spore traps in the Czech Republic at low levels, seemingly unaffected by *H. fraxineus* abundance (Dvorak et al., 2015; Koukol et al., 2016). Coexistence of the harmless native species with an invasive sister species has also been observed in the case of *Cryphonectria radicalis* (Hoegger et al., 2002). In this study, *H. albidus* was only found at very low levels in a southern alpine valley where *H. fraxineus* had arrived recently. At time of the arrival of the disease in Switzerland (2007–2009), the fungus could be found north and also south of the Alps (Queloz et al., 2011; Senn-Irlet et al., 2016). It is possible that the species became very rare or went extinct and was therefore not found north of the Alps. However, we did not find *H. albidus* in the disease-free sampling sites either. The species might be generally rare and may therefore not have been found by coincidence, or its abundance might have been below the detection limit. Alternatively, the southern distribution limit of the fungus could have been reached (Baral and Bemmann, 2014). At least *H. fraxineus* seems to be affected by high summer temperatures (Grosdidier et al., 2018).

Negative effects or even extinctions due to reduced host densities are expected for endophytes with a host preference. Jönsson and Thor (2012) predicted reduced species richness and local extinctions of lichens associated with *F. excelsior* due to ash dieback. The outcome of the simulation highly depended on the mortality scenario. Predicting the survival of the European ash is difficult given the still limited knowledge and variable impacts on different countries (Vasaitis and Enderle, 2017). In Lithuania (one of the first infested countries), *F. excelsior* stand area was reduced to half within 19 years, and currently an annual mortality rate of 9% is estimated (Pliura, 2017). Once arrived to Central Europe, the emerald ash borer (*Agrilus planipennis*) is expected to significantly increase mortality, but the exact impact is yet unknown (Musolin et al., 2017; Valenta et al., 2017). In addition, the appearance of even more virulent *H. fraxineus* genotypes cannot be excluded, for instance in the case of the introduction of more genotypes from its native range (Gross et al., 2014a; Landolt et al., 2016). On the other hand, climate change could lead to reduced disease incidence in warmer areas (Goberville et al., 2016; Grosdidier et al., 2018). Complete extinctions of tree species after introduction of an alien pathogen have not been observed to date and is not expected for *F. excelsior* (Landolt et al., 2016).

In our study, most species were found on all three hosts and can therefore be viewed as generalists not severely affected by ash decline. However, we found a strong host specificity of the dominant endophyte *Venturia fraxini*, which is in line with existing evidence (Ibrahim et al., 2016). The low level of ‘non-host’ amplicon is likely derived from methodological artifacts (crosstalk; Edgar, 2016b). The specificity of *V. fraxini* and *V. orni* for their hosts has been confirmed by cross-inoculation experiments using conidia (Ibrahim et al., 2016; Schlegel et al., 2016). The detection of two additional leaf tissue specific *Venturia* species on *A. pseudoplatanus*, which are possibly different from *V. aceris* suggests a high diversification due to host specialization.

Other abundant endophytes with host preferences belonged to the Mycosphaerellaceae. *Mycosphaerella* spp. are assumed to be host specific, although there are exceptions (Crous and Groenewald, 2005). Most species were amplified from samples of both *Fraxinus* species, although less abundantly from *F. ornus*. However, the isolation frequencies by Ibrahim et al. (2017) were similar as for *F. excelsior* in this study. We therefore assume that most, if not all Mycosphaerellaceae found on *F. excelsior* may be able to occur in *F. ornus* leaves as well. Interestingly, *Ramularia lethalis*, a known *Acer* associate (Videira et al., 2016), was also amplified from *F. excelsior* leaves in this study. Other *Mycosphaerella* spp. potentially specific for *Fraxinus* spp. were in turn amplified from some *A. pseudoplatanus* samples at low levels. We cannot exclude that surface sterilization was less effective for this group compared to *Venturia* spp., possibly due to massive spore deposition. In addition, it is possible that the ITS2 region did not provide enough resolution to separate *R. lethalis* from other species. On the other hand, occasional ‘host-jumps’ of pathogenic *Mycosphaerella* spp. have been observed (Crous and Groenewald, 2005; Crous et al., 2008).

Nothing is known about the minimum viable population of *V. fraxini* to avoid extinction, but survival seems likely given the abundance and wide geographic distribution of the species, unless *F. excelsior* would disappear completely. The fungus was also able to establish on planted *F. excelsior* in New Zealand, where the tree had been introduced in the 19th century along with at least one third of the fungal endophytes (Power et al., 2017). The sister species *Venturia orni* was also very abundantly detected in isolated planted *F. ornus* trees north of the Alps. Members of the Mycosphaerellaceae were generally found on both sides of the Alps, and survival on *F. ornus* should thus be possible. On the other hand, their distribution was also highly variable and none of the sampling sites harbored all species. The same was true for rare *Venturia* spp. on *F. ornus* and *A. pseudoplatanus* (Figure 8). Shifts in community structure, reduced genetic diversity and local extinctions due to low tree densities are thus possible. Even abundant endophytes can be significantly affected by reduced tree densities, as found by a study on birch endophytes on islands near the shoreline. The frequency of the dominant endophytes (including a *Fusicladium* species with a *Venturia* teleomorph) increased with island size and stand density, but decreased with distance from the mainland, presumably due to a reduced inoculum (Helander et al., 2007).

To obtain an overview of the *Fraxinus* mycobiota over a large geographic distances, we also did a comparison of leaf and twig fungi of *Fraxinus*, for which ITS2 sequences have been published, including two NGS studies (Supplementary Methods 1.11). The summary table (Supplementary Table File 5) confirms a characteristic mycobiome consistent across a large geographic area and different *Fraxinus* species. There was even a substantial agreement between the abundant leaf fungi of *F. excelsior* in Europe and the native *H. fraxineus* host *F. mandshurica* in Eastern Russia (Cleary et al., 2016). *Venturia fraxini* was mainly detected in leaflets and petioles, either asymptomatic (Scholtysik et al., 2012; Cross et al., 2017), necrotic petioles (Bakys et al., 2009b) or in leaf litter (Davydenko et al., 2013). The species was absent from shoots (Bakys et al., 2009a; Kowalski et al., 2016; Kosawang et al., 2017) with only one exception (Bakys et al., 2009b). Interestingly it was only rarely amplified from leaves in Norway (Cross et al., 2017) and not reported from *F. mandshurica* (Cleary et al., 2016).

By comparing the *F. excelsior* leaf mycobiota in the diseased and disease-free area of Switzerland, we also aimed at finding hints for a past influence of ash dieback on community structure. Since *A. pseudoplatanus* is not affected by an epidemic, it could be viewed as “control” organism for estimating the influence of climatic and edaphic factors on endophytic fungi. Indeed, significant differences were found for *F. excelsior*, but not for sycamore maple (*A. pseudoplatanus*). On the other hand, alpha diversity was not lower on the north side of the Alps, suggesting no biodiversity loss due to ash dieback. In addition, most OTUs with a differential north-south distribution on *F. excelsior* tended to be specific for *Fraxinus* hosts, and therefore a comparison with sycamore maple was not possible on an individual OTU level. The only generalist, *Cladosporium* sp., followed a similar geographic distribution pattern on all host species. It is therefore not possible to conclude whether differences on *F. excelsior* were caused only by the presence/absence of ash dieback. More evidence could be obtained by repeated sampling over a longer time period during establishment of the disease.

### Interactions With Tree Health

Beneficial and protective effects of bacterial and fungal microbiota on their plant hosts have been found by numerous studies and the importance of microbiome-based biocontrol strategies is expected to increase (Berg et al., 2017). Similarly, there is a growing body of evidence about effects of tree endophytes (Busby et al., 2016; Witzell and Martín, 2018).

Most shoot infections by the ash dieback pathogen are caused by genotypes that originated from leaves and crossed the petiole-shoot junction (Haňáčková et al., 2017b). Although direct entrance to the petiole is possible (Cleary et al., 2013), more infections are expected to happen in leaflets due to the much larger surface area. Penetration of host cells is followed by a biotrophic phase before necrotic lesions occur (Mansfield et al., 2018). Consistently, *H. fraxineus* was abundantly found in asymptomatic leaves by this study. During this phase, endophytic fungi in both the leaf blade and the petiole might be able to inhibit the progression of the pathogen.

Up to one third of the common *F. excelsior* leaf and shoot endophytes were found to produce antibiotic compounds that inhibit germination of *H. fraxineus* ascospores (Schlegel et al., 2016) and mycelial growth (Schulz et al., 2015; Haňáčková et al., 2017a; Kosawang et al., 2017). The isolates tested by Schlegel et al. (2016) and their inhibition rates can be found in Supplementary Data Sheet 2. *Paraconiothyrium* sp., which was frequent on the south side of the Alps, showed a very strong inhibition of *H. fraxineus* spore germination. The species seems rare or absent in the north (Supplementary Table File 5), but interestingly it was also found in leaves of *Fraxinus mandshurica* in East Russia (Cleary et al., 2016; OTU_15).

*In vitro* screening for antagonisms may not necessarily reveal the most effective candidates for biocontrol as protection is also possible through niche competition or indirect activation of the plant immune system (Eyles et al., 2010; Shoresh et al., 2010; Busby et al., 2016; Berg et al., 2017). Therefore, *in planta* analyzes and experiments are important. In a previous study, we found no effect of pre-inoculated *V. fraxini* and other leaf endophytes on *H. fraxineus* abundance after 40 days of exposure to the pathogen (Schlegel et al., 2016). Still, a more targeted inoculation of endophytes with a longer period of exposure to the pathogen might be successful in finding *in vivo* antagonisms. Indications of an antagonistic effect could be derived from a negative frequency relationship between *H. fraxineus* and other leaf inhabitants. However, only one species (*Setophoma* sp.) with a weak negative association was found. The closest BLAST hits are the leaf spot causing *Setophoma vernoniae* (96.6%) and *S. chromolaena* (96.9%). A distantly related *Neosetophoma* isolate did not show an inhibition of *H. fraxineus* ascospore germination (Schlegel et al., 2016).

We also examined whether leaf endophytic communities were different in healthy leaves of *F. excelsior* trees with or without symptoms of ash dieback. Tree health has been linked to an altered microbiome, with variable success (Gennaro et al., 2003; Martín et al., 2013; Koskella et al., 2017; Bullington et al., 2018). No OTUs with clear preferences for symptomless or symptomatic *F. excelsior* trees were found. However, two species (*Diaporthe rudis, Boeremia sp.*) were slightly more frequent on trees with visible symptoms of ash dieback. Interestingly, Haňáčková et al. (2017a) also found two *Diaporthe* spp. being more frequent on shoots of diseased trees, while *D. eres* was more abundant on trees without symptoms. However, *D. eres* appears to be a weak pathogen of the European ash (Bakys et al., 2009b; Kowalski et al., 2016). *D. eres* was also more abundant on symptomatic trees in this study, although the effect was not significant (not shown).

Host genetic background was found to be a strong determinant of tolerance to ash dieback (McKinney et al., 2011; Harper et al., 2016). In a comparison of metabolic profiles of extracts from (apparently asymptomatic?) leaflets, susceptible trees were found to produce more iridoid glycosides, which are known as anti-herbivore defense compounds in the Oleaceae, but also to influence fungal growth (Sollars et al., 2017). It is possible, that the detected frequency changes of *Diaporthe rudis/eres* were caused by changes in secondary metabolite production by the host. It has to be noted that selecting trees without symptoms of ash dieback proved to be difficult on some sampling sites. This is not surprising, since only a small percentage of *F. excelsior* is tolerant toward the disease (McKinney et al., 2014). The trees sampled in this study were not monitored for disease symptoms in the following years and the most tolerant/resistant and susceptible trees might therefore not have been chosen.

## Conclusion

While a complete extinction of the European ash is very unlikely, highly reduced densities due to ash dieback and the emerald ash borer are expected. Not only fungi, but also many other associated species including bacteria, insects, molluscs, mosses (Pautasso et al., 2013) and lichens (Jönsson and Thor, 2012) may experience shifts in community structure, loss of genetic diversity and local species extinctions. Part of the *F. excelsior* endophytic mycobiota showed more or less strong host preferences and may therefore be affected in the future. We also found a high geographic variability and differences between the north and south side of the Alps for *F. excelsior*. Most species were detected by isolation and NGS sequencing, confirming the suitability of both methods. Evidence about possible roles of the mycobiome in enhancing host tolerance against ash dieback remains inconclusive. This study provides a precise picture of the ash and maple mycobiota, which enables more profound future research about possible interactions with the host holobiont and the pathogen.

## Data Availability Statement

The raw sequence data are available on GenBank (study accession: SRP148813). Putative contaminant sequences were not included. The ITS sequences of the cultured isolates listed in Supplementary Data Sheet 2 are available under the accessions MH934980-MH935083. The shell scripts and R scripts used for the primer analyses are available in Supplementary Data Sheet 4. Additional python scripts were uploaded to GitHub (https://github.com/markschl/bio_scripts). A fast tool for handling large amounts of sequences was written by the author in the process of this study (https://github.com/markschl/seqtool).

## Author Contributions

All authors planned and performed the leaf sampling and processing of the samples. MS planned and conducted the primer test, mixed the mock communities, did the processing and amplification of the leaf samples, bioinformatic and statistical analyses, and wrote the manuscript. TS supervised the project and developed it together with MS.

## Conflict of Interest Statement

The authors declare that the research was conducted in the absence of any commercial or financial relationships that could be construed as a potential conflict of interest.

## Footnotes

1 http://blast.ncbi.nlm.nih.gov

2 https://github.com/markschl/seqtool
