# Supplementary material for: The Endophytic Mycobiome of European Ash and Sycamore Maple Leaves – Geographic Patterns, Host Specificity and Influence of Ash Dieback
Source: Front Microbiol. 2018 Oct 24;9:2345. doi: 10.3389/fmicb.2018.02345 (PMC6207852; doi:10.3389/fmicb.2018.02345)
Supplement: Supplementary file 6 [file Data_Sheet_1.pdf]

Supplementary Material

The endophytic mycobiome of European ash and sycamore maple leaves – geographic patterns, host specificity and influence of ash dieback

Markus Schlegel[1], Valentin Queloz[2], Thomas N. Sieber[1*]

*** Correspondence:** Thomas N. Sieber: thomas.sieber@usys.ethz.ch

1 Supplementary Methods

1.1 Coverage analyzes of 5.8S and LSU primers

Ribosomal sequences were retrieved using the following GenBank query (2017-11-08):

```
(28S[Title] OR lsu[Title] OR "large subunit"[Title])
 AND (ribosomal[Title] OR ribosome[Title] OR rRNA[Title] OR rDNA[Title])
 AND Eukaryota[Organism]
 AND 20:10000[SLEN]
NOT (genome[Title] OR scaffold[Title] OR chromosome[Title] OR supercontig[Title] OR
mitochondrial[Title] OR mitochondrion[Title] OR protease[Title] OR proteasome[Title] OR
protein[Title]
```

The resulting 1,491,269 sequences were clustered at 70% identity using VSEARCH (Rognes et al., 2016), pre-sorted by decreasing length. Then, ITSx (Bengtsson-Palme et al., 2013) was used to find rDNA domains within the cluster centroids. The resulting domain coordinates for 5.8S and LSU were used to extract the subsequences of these domains from the SAM alignments produced by VSEARCH. The intermediate clustering step largely reduced the time necessary for the domain search. The 5.8S domain was found in 67.8% of the sequences, while the LSU domain was found in 48.4%. Primer matches were found using the USEARCH *search_oligodb* command (v10.0.240, https://www.drive5.com/usearch) with a maximum of 10 substitutions allowed. 22 bp were removed from the sequence ends because many sequences on GenBank tend to contain the sequencing primers. This also removed useful information, but should be safer, especially because ITS3 and ITS4 are popular sequencing primers, and not removing them may lead to wrong results. Some sequences were shorter and did not contain all primer binding sites. In order to reduce the number of unspecific matches, the positions of the rDNA domains were determined for each sequence using *hmmsearch* (http://hmmer.org, version 3.1b2). The HMM profiles were constructed from the 5_8S_rRNA and LSU_rRNA_eukarya (columns 1-63) seed alignments from the Rfam database (http://rfam.xfam.org). Only USEARCH primer hits positioned at the expected location within the domains were considered for the mismatch analysis.

Taxonomic information about each sequence was retrieved with the help of Taxadb (https://github.com/HadrienG/taxadb) and the Entrez web service (*get_taxonomy.py* script from https://github.com/markschl/bio_scripts). Primer mismatch summaries were done by counting the number of unique species. No additional filtering was done (e.g. removal of uncultured records or sequences not classified to the species level but assigned a higher rank only). All graphs and analyzes were done in R with the help of ggplot2. For plants, all search results are shown and summarized in **Table file 3**.

See also script *primer_validation/01_primer_mismatches_Genbank.sh,* **Data Sheet 4**.

1.2 Analysis of terminal mismatches to fungi

The UNITE database (Kõljalg et al., 2013) was used for finding mismatches within the six terminal 3' bases at the annealing site of the primers ITS4f/ITS4f2. The UNITE+INSD dataset (2017-10-10 release, 777,046 sequences) was downloaded from https://unite.ut.ee/repository.php, and clustered at 85% identity using VSEARCH. The LSU domain was found in 491,030 of the sequences using the same procedure as above with the GenBank dataset. For finding the correct positions, sequences were aligned against a HMM profile constructed from the LSU_rRNA_eukarya seed alignment from the Rfam database (http://rfam.xfam.org/family/RF02543), positions 1-60 (5' end of ITS4f primer). Both the sequences and the HMM profile were reverse complemented to minimize the number of misaligned residues. Alignment was done with *hmmalign* (http://hmmer.org, version 3.1b2). Frequencies of the six residues of interest were determined in R. Sequences were not trimmed at the end even though many of them may still contain the primer sequence. However, it was assumed that most if not all of the sequences containing the ITS4f/ITS4f2 primer site were sequenced with ITS4 or another primer more downstream. Thus, the last 6 bp of the primer site should be correct since they do not overlap with ITS4. For species with deviating sequences with a certain abundance (> 3 sequences and > 1.5% of all sequences), a manual validation was done by inspecting full-length alignments produced from the UNITE sequences (see **Table File 1**).

See also script *primer_validation/02_ITS4f_end_UNITE.sh*, **Data Sheet 4**.

1.3 Mock community construction

24 fungi from different taxonomic groups were selected (listed in **Table S4** and **Table File 4**). 12 of the species were previously isolated from *F. excelsior* leaves. The fungi were cultured on malt agar (20 gl^{-1} malt extract, 15 gl^{-1} agar). In order to obtain pure cultures, single (if possible) or few hyphae were cut from the culture margin and transferred to a new plate. For yeasts (*S. cerevisiae, E. hasegawianum, P. flavescens*), a single colony was obtained by dilution plating. From those new cultures, a standardized inoculum was excised using a cork borer (4 mm diameter) and transferred to Erlenmeyer flasks containing 60 ml of 2% malt extract. The flasks were incubated at 20 °C in the dark with constant shaking at 80 rpm. After incubation for different time periods (see **Table File 4**), mycelium was transferred to a sterile filter membrane (Whatman / GE Healthcare) and the liquid was removed by aspiration. Yeasts (including *A. pullulans* which grew as yeast in liquid culture) were cultured in a total of 150 ml malt extract for 3.9 days. In order to aggregate cells, the medium was centrifuged at 2500 rcf for 5min and the supernatant discarded. 70-100 mg tissue was then transferred to 2 ml Eppendorf tubes and stored frozen at -80 °C. For homogenization, two metal beads (7 mm and 4 mm diameter) and a few silica beads (1 mm diameter) were added. Samples were homogenized in a frozen state using a cooled BeadRuptor 24 mill (Omni international, Kennesaw, USA) for 2 x 30 s at 2.25 m/s. DNA was extracted using the NucleoSpin® Plant II kit (Macherey-Nagel) according to the manufacturer's instructions, using the PL1 lysis buffer. Extraction quality was ensured by checking for fragmentation on an agarose gel. Only extractions without visible fragmentation (smear) were used. DNA concentration was measured using the Qubit dsDNA BR assay (Thermo Fisher Scientific). All gDNA extractions were then diluted to a final concentration of 0.75 ng μl^{-1} in 5 mM Tris/HCl buffer. Dilution was done in three steps with intermediate Qubit measurements. The final measurement was done with Qubit dsDNA HS in three replicates. Subsequently, an even mixture was created by adding an equal amount (ng) of each gDNA to the pool. Additionally, two uneven mock communities with geometric abundance distributions with different values for the *k* parameter were mixed (**Table File 4**, workbooks 'dilution_uneven_1/2'). In order to achieve precise mixtures at a high dilution range, four blocks of six consecutive dilutions were mixed separately. The sub-pools were then serially combined to create the final pool. Pipetting of

the three mixtures was done independently three times. The frequencies of the individual species were randomly assigned using R. The mixes were stored in aliquots at -80 °C until used.

The ITS region of the fungi was amplified using two self-designed primers located on the SSU (5'-CTTTGTACACACCGCCCGTC-3') and LSU (5'-CCGSTTCACTCGCMGTTACT-3'). The PCR product was sequenced from both ends using Sanger sequencing. The primers ITS1F, ITS3 and ITS4 were used for re-sequencing in case of failure. All sequences were aligned and edited in Geneious 6.1 (Biomatters, New Zealand) to obtain a full-length sequence of the complete ITS region including parts of the SSU and LSU.

Since the genome size and number of rDNA repeats was unknown for most species, a qPCR quantification was done to determine relative rDNA content per species and get a more precise estimation of the expected amount of ITS amplicon for each species. A conserved 108 bp fragment of the SSU region was amplified using newly designed primers (forward: 5'-YAATTATTGCTCTTCAACGAGGA-3', reverse: 5'-ATTCAATCGGTASTAGCGACG-3'). The degeneration at the 5' end of the forward primer was applied because of the deviating sequence of *S. cerevisiae*. However, the results might still not be as precise for this species as for the others. Amplification was done in triplicates in a reaction volume of 10 µl containing 0.2 µl of each primer (10 µM), 2 µl of 5 x FIREPol® EvaGreen® qPCR Mix Plus (ROX) (Solis BioDyne, Tartu, Estonia), 1 µl gDNA and 6.6 µl PCR-grade water. Cycling was done on a 7500 Fast RT-PCR (ABI / Thermo Fisher Scientific) with the following conditions: initial denaturation at 95 °C for 15 min, followed by 40 cycles of 95 °C for 15 s and 55 °C for 1 min. The PCR efficiencies were inferred from the slopes of the amplification curves using LinRegPCR (2014.2, Ruijter et al. 2009). The window of linearity was calculated separately for each species, and the starting concentrations (N0, normalized fluorescence units) were calculated based on the mean efficiency of the three replicates.

1.4 Determination of linker sequences for primers

The purpose of the 2-bp linker added at the 5' end of each primer was to prevent binding of the frameshift nucleotides or the adapter to fungal sequences. It was therefore chosen to be as rare as possible in the sequence databases. The dinucleotide frequencies were determined from alignments of parts of the 5.8S and LSU domains of the UNITE OTUs (2016-08-22, including singletons, clustered at dynamic threshold). The alignment was done through a *hmmsearch* (http://hmmer.org) against profiles constructed from fungal 5.8S and LSU alignments by James et al. (2006). For each primer, a dinucleotide linker not occurring upstream in the alignment was selected.

See also bash script *primer_validation/03_linker_analysis.sh* in **Data Sheet 4**.

1.5 Construction of the taxonomic reference database

For fungal taxa, the USEARCH/UTAX reference dataset from UNITE (2017-10-10, https://unite.ut.ee/repository.php) was used. Other eukaryotic sequences were downloaded from Genbank using the following query:

```
"internal transcribed spacer 2" OR "internal transcribed sequence 2" OR ITS2 OR "ITS 2"
OR "ITS-2" OR ITS)
  AND ("50"[SLEN]: "5000"[SLEN])
NOT ("Fungi"[Organism] OR chromosome OR scaffold OR chloroplast OR intergenic OR
cytochrome OR histone OR exon OR 16S OR 23S OR matK OR tubulin OR elongation OR protein)
```

The downloaded sequences were additionally cleaned by excluding mitochondrial, chloroplast rDNA and non-ribosomal sequences by keywords. The taxonomy was retrieved using Taxadb (https://github.com/HadrienG/taxadb) and the Entrez web service (*get_taxonomy.py* script). The sequences were then clustered at 70 % identity using VSEARCH, and assigned a consensus taxonomy (*tax_consensus.py*). ITSx was then used to extract the ITS2 region. Unrecognized cluster sequences were further searched for keywords in their headers that suggested the presence of the ITS2 region. Those sequences were included as well in the taxonomic database.

See also script *taxonomy_db.sh*, **Data Sheet 4**.

1.6 Chimera detection in mock community reads (Figure S6B)

In order to precisely determine the content of chimeric reads, we used the known sequences of the mock communities as reference database for the *uchime2_ref* command from USEARCH v10.0.240 (Edgar, 2016a). The command was run with the "balanced" setting.

1.7 Pylogenetic tree of *Venturia* spp. and Mycosphaerellaceae (Figure 8 in main text)

Putative *Venturia* OTUs were found using a global search (usearch_global command of VSEARCH) against all *Venturia* spp. ITS sequences published by Ibrahim et al. (2016) at an identity threshold of 93%. From matching OTUs, the most abundant (> 1000 reads in total) were selected. In addition, a selection of sequences from related species were included (Crous et al., 2007). For the Mycosphaerellaceae, OTUs were selected based on the automatic annotation by USEARCH if present with > 200 reads.

Both sequence sets were aligned using MAFFT (v7.130b; Katoh and Standley 2013) using the G-INS-i algorithm with 1000 cycles, and positions with end gaps or > 75% internal gaps were removed in Geneious. The phylogenetic trees were constructed using the RAxML BlackBox server (Stamatakis et al. 2008; https://embnet.vital-it.ch/raxml-bb), which generated 100 trees using the GTR+CAT model and selected the best-scoring ML tree (maximum likelihood search option). The calculations were done without specific outgroups. The figure was created in R using *ggtree* (Yu et al., 2017) and *ggplot2*.

1.8 Statistical models used for finding differences in community structure (PERMANOVA)

Permutational analysis of variance (PERMANOVA) was done using the *vegan* package (Oksanen et al., 2017) for different subsets and different factor combinations (**Table S2**). Apart from a general test for geographic and between-host variability, the sampling site was included as a stratum ("random effect") constraining the permutations to these groups. In these models, the site effect was not of primary interest. In order to make the sample sizes more balanced (8 trees per group), a separate stratum for each site/health status combination was specified for *F. excelsior*. The R code was:

```
perm <- permute::how(nperm=99999, plots=permute::Plots(stratum))
vegan::adonis2(abundance_clr ~ factors, permutations=perm, data=data_subset)
```

1.9 Statistical models used for finding differentially distributed taxa

The models were calculated for each OTU and each Monte Carlo (MC) sample provided by *ALDEx2::aldex.clr*. A linear mixed model with one variable of interest (fixed effect) was used, and the sampling site and the number of PCR cycles were included as random effects (**Table S3**). The calculations were done for each group described in the "Subsets" column of this table. R code for the mixed-effects model:

```
m <- lme4::lmer(abundance_clr ~ factor + (1|plot) + (1|cycles), data=data_subset)
```

1.10 Clustering of raw sequence data from Cross et al. (2017)

All Ion Torrent PGM sequence data from BioProject PRJNA305543 was downloaded and quality filtered at a maximum error rate of 0.005 (1.5 err. per 300 bp) using USEARCH. Clustering was done using the UNOISE3 algorithm in USEARCH using a low minimum OTU size (-minsize) of 2. The OTU table was constructed by mapping the unfiltered reads against the OTUs using VSEARCH. Taxonomic annotation, searching for the ITS2 region and removal of unspecific sequences was done as described for this paper.

See also script *literature_comparison/1_cluster_cross.sh*, **Data Sheet 4**.

1.11 Survey of published ITS sequences from *Fraxinus* fungi

In order to compare the results of this study with existing studies on the mycobiome of *Fraxinus excelsior* and other *Fraxinus* species, their isolate sequences from several studies were downloaded from GenBank (Bakys et al., 2009a, 2009b; Davydenko et al., 2013; Kosawang et al., 2017; Kowalski et al., 2016; Scholtysik et al., 2012). In addition, OTUs from two NGS studies were included (Cleary et al., 2016; Cross et al., 2017). The first study provided only the top 20 OTU sequences. Cross et al. (2017) provided raw sequence data, which was clustered as described in **Supplementary Methods 1.10**. Only samples collected within a timeframe comparable to this study (mid to late August) were selected. For other studies, all data was included, even if derived from samples collected earlier in the year. The isolate and OTU sequences were clustered at a 98% threshold using USEARCH *cluster_fast*. The ITS2 OTUs from the NGS studies were positioned before the longer Sanger sequences, thus forming most of the cluster centroids. This was done to ensure consistent clustering despite of the variable sequence lengths.

The isolate frequencies and read counts were extracted from the mentioned publications and summed up for each of the 98% clusters. The same was done for the OTUs from this study and from Cross et al. (2017). The results are summarized in **Table File 5**.

See also script *literature_comparison/2_cluster_all.sh*, **Data Sheet 4**.

2 Supplementary Results

2.1 Read processing

The two sequencing runs yielded in 14.4 and 15.2 million (M) reads. A total of 21.4 M reads were assigned to samples from this experiment. 2.9 M reads from the second run were discarded by random selection for samples where too much indexed product had been spiked. Strict quality filtering removed another 4.7 % of the reads. The sample sizes (without negative controls) ranged from 460 – 94,631 with 96% of the samples being in the range 27,690 - 69,599 (median: 48,770). Rarefaction curves suggest that most of the OTU diversity was captured (**Figure S5**). Only few sequences (0.6%) were classified as unspecific.

2.2 Quality control

A single OTU/species pair with 100% identity could be found for all mixed species (**Figure S6A**). There was one exception, where two OTUs were found for *Backusella tuberculispora.* However, this was expected since Sanger sequencing of the gDNA extract from this species already showed an ambiguous position within the ITS2 region, suggesting two sequence variants. Apparently, no pure culture had been obtained for this species.

The number of chimeric reads was low, although increasing with more PCR cycles in the tailing step (**Figure S6B**). The number of singleton OTUs did not substantially increase with cycle numbers (**Figure S7B**). However, the recovery of rare species of PCR cycles was clearly lower with higher cycle numbers: The more cycles were done, the more rare species in the uneven mock community failed to amplify (**Figure S6C**). Very rare species (< 5 reads at 19 cycles) were not present any more at 24-33 cycles. Similarly, OTUs with roughly < 50 reads (frequency of 0.2%) at lower cycle numbers were not amplified at 29/33 cycles, and OTUs with < 200 reads (0.8%) were missed at 33 cycles. In accordance to this finding, the relative frequency of rare OTUs in the leaf samples decreased with higher cycle numbers (**Figure S7A**).

For each cycle group, a negative control was included. One of them contained 1643 reads of an OTU, which was almost undetectable in "real" samples. Therefore, this OTU was removed from the analyzes. In addition, other "unexpected" reads could be detected in both the negative controls and the mock community samples, which were most likely derived from environmental samples and incorrectly assigned to the control barcodes. This phenomenon that has been referred to as *crosstalk* (Edgar, 2016b). The quantification of the effect is described in the next chapter.

The presence of some species in the dataset raised suspicion because they were not expected to occur endophytically in leaves. Therefore, possible contaminations during DNA extraction were examined using indicator species analysis using the *multipatt* function from the indicspecies R package (De Cáceres et al., 2010). The leaf samples had been extracted in batches of 30, and about half of the samples of each batch were analyzed by NGS amplicon sequencing. Therefore, the extraction series was used as grouping variable and the analysis was run allowing single sites and combinations of two sites. While there was no indication of contamination for most extraction series (1-2 randomly significant species), several OTUs associated with a combination of two series were found, many of them being identical to the unexpected OTUs. Further examination revealed that the contamination had been present undetected for a time frame of several consecutive extractions. Since all species showed the same pattern of occurrence independent of biological variables, they were excluded from further analyzes, as well as all OTUs with > 95% sequence similarity to them.

2.3 Quantification of crosstalk

The abundance of "unexpected" OTUs in the negative and mock controls increased with increasing total OTU sizes (**Figure S9**), leading to the conclusion most of these reads were probably derived from crosstalk. Quantile regression with tau=0.995 was used to predict the maximum rate of misassignment. Log10-transforming the OTU sizes and allowing a negative intercept seemed to improve the prediction. This non-linear relationship may be explained by a sampling effect. According to this analysis, a maximum of 6 misassigned sequences per sample were predicted for the most abundant OTU (OTU 2; *Venturia fraxini*). Crosstalk may also explain, why this OTU did not disappear from the uneven mock community at higher cycle numbers like other rare species did (**Figure S6A**).

For three figures in the main text (**Figure 5B**, **Figure 7**, **Figure 9A**), potential crosstalk was removed to improve readability. This was done by setting read counts below the predicted maximum number of misassigned reads to zero. No correction was done for the other figures and for statistical analyzes since the approach at determining misassignments is entirely empirical and the properties of crosstalk are poorly understood (Edgar, 2016b).

3 Supplementary Discussion

3.1 Advantages and limitations of the fungal-specific primers ITS4f and ITS4f2

ITS4f and ITS4f2 showed a strong specificity for fungi despite of the low number of differences (two and one, respectively) to most plant sequences. This can be explained by the fact that mismatches within the last 3-4 bases of a primer have a much stronger effect on amplification efficiency of *Taq* polymerases than internal mismatches (Huang et al., 1992; Kwok et al., 1990; Wu et al., 2009). A single difference can be enough to strongly reduce amplification. Achieving such an effect with proofreading polymerases with a 3’ to 5’ exonuclease capability is only possible if the nucleotides at the 3' end are protected from being 'corrected', e.g. by using phosphorothioate internucleotide linkages (Zhang and Li, 2003). The use of high fidelity polymerases in amplicon sequencing studies is highly advisable since they exhibit lower PCR error rates and produce less chimeric molecules compared to the *Taq* polymerase (Gohl et al., 2016).

During the course of our study, a similar primer named ITS4-Fun has been developed independently by Taylor et al. (2016). Its 3' end is identical to the ITS4f2 primer, but it is longer (25 instead of 21 bp) and has no internal degeneration (see **Table S5**). The selectivity of ITS4-Fun has been confirmed by Illumina sequencing after amplification with a *Taq* polymerase. Taylor et al. (2016) reported a high coverage of ITS4-Fun among sequences of the SILVA LSU database. We confirmed this finding for our primers using all sequences available on GenBank. In addition, we focused on the 3' end of ITS4f/ITS4f2 since a downside of relying on highly selective 3' mismatches is that even single deviations to fungal sequences can have a detrimental effect on their amplification, eventually leading to total exclusion of these species from the analysis. Much care was taken to identify mismatching taxa in the fungal kingdom as listed in **Table S5** and **Table File 1**. The two major groups with mismatches to both ITS4f and ITS4f2, *Tulasnella* and Microsporidia are generally not well amplified by many ITS primers (Tedersoo and Lindahl, 2016). Additionally, part of the *Xylaria cubensis* sequences were found to have 4-5 mismatches to the ITS4f/f2 primers, as well as ITS4. The isolates originate mainly from above-ground parts of tropical (Higginbotham et al., 2013; Higgins et al., 2011; Olmo-Ruiz and Arnold, 2014, 2017; Rukachaisirikul et al., 2013), subtropical (Osamu Tateno et al., 2015) and temperate (Brooks et al., 2017; U’Ren et al., 2016) plants. *X. cubensis* was also abundantly found in lichens (Soca-Chafre et al., 2011; U’Ren et al., 2012, 2016) and orchid roots (Herrera et al., 2010; Jiang et al., 2011), and was even detected in marine sponges (Bolaños et al., 2015). *X. cubensis* has a high genetic diversity and might actually be a complex of sev-

eral species (U'Ren et al., 2016). It may be even more widespread, but was not captured in amplicon studies due to the mismatches to ITS4. Interestingly, there seems to be a lack of reports about difficulties in amplifying this species.

Other species from basal lineages and the Peltigerales have one mismatch towards the ITS4f primer and none to ITS4f2. In the pond sediment sample, no mismatching OTUs of clearly fungal origin could be identified, although the taxonomic assignments were not always possible with certainty.

Selecting between the two primers tested in this study (ITS4f and ITS4f2) results in a trade-off: The additional degeneration of ITS4f2 allows amplification of most fungal taxa, but also reduces the selectivity of the primer. NGS sequencing of an *F. excelsior* leaf sample with ITS4f2 yielded a high fraction (> 50%) of plant amplicon, therefore, the ITS4f primer was used, at the risk of missing some taxa. However, this risk was small; based on sequences from cultured isolates and the literature about the *Fraxinus* mycobiome, no species was expected to have mismatches to the primer. *X. cubensis* was never isolated in our study even though it is culturable (Rogers, 1984; U'Ren et al., 2016).

3.2 Petiole specific *Malassezia* spp.

Interestingly, species more abundant in petioles also included *Malassezia* spp., which are well known as colonizers of the skin surfaces of animals, including humans. However, we believe that it is unlikely that the reads were derived from laboratory contamination. Species of this genus are cosmopolitan and also found in plants (Amend, 2014; Tondello et al., 2012). Moreover, *M. restricta* and *M. globosa* have been amplified from *F. excelsior* wood samples in New Zealand (Power et al., 2017). A re-analysis of the raw sequence data from a study on endophytic and epiphytic *F. excelsior* fungi in the course of the growing season (Cross et al., 2017; **Supplementary Methods 1.11**) revealed a low level of *Malassezia* spp. In accordance to our results, the species was almost only present in different parts of the petiole (**Figure S21**).

3.3 Recovery of rare species in the mock communities

The species from the uneven mock community were generally recovered at a good precision. However, it was obvious that the amplification of diluted communities (and also environmental samples) with many PCR cycles lead to a bad recovery of rare species (**Figure S6A/C**; **Figure S7A**). This effect was especially strong at 33 cycles, but also visible at 29 and less cycles. The reason is likely dilution leading to "extinction" of rare template molecules. For instance, most petiole samples had a lower amount of total genomic DNA (and also less fungal DNA) than lamina samples and were therefore mostly amplified with 29 PCR cycles. It was observed that rare species with a frequency of approximately < 0.2% were missed in the staggered community (**Figure S6A**), which had been diluted to 0.31 pg μl^{-1} in order to match the approximate fungal gDNA concentration in the leaf samples. A frequency of 0.2% roughly corresponds to a total of 5 template ITS molecules in the three replicate reactions (**Table S7**). At such low numbers, the influence of stochastic events is high.

4 Supplementary Figures

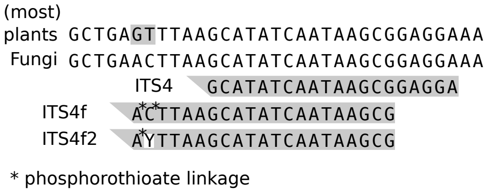

Figure S1: Sequence comparison of the new primers ITS4f/ITS4f2 and ITS4 (reverse complement) with the most prevalent plant and fungal LSU sequences. The two nucleotides differing in most plants are highlighted in grey in the plant sequence. ITS4f2 differs from ITS4f by an additional degeneration and only one phosphorothioate linkage (*). The primer sequences in their forward orientation are found in Table 1.

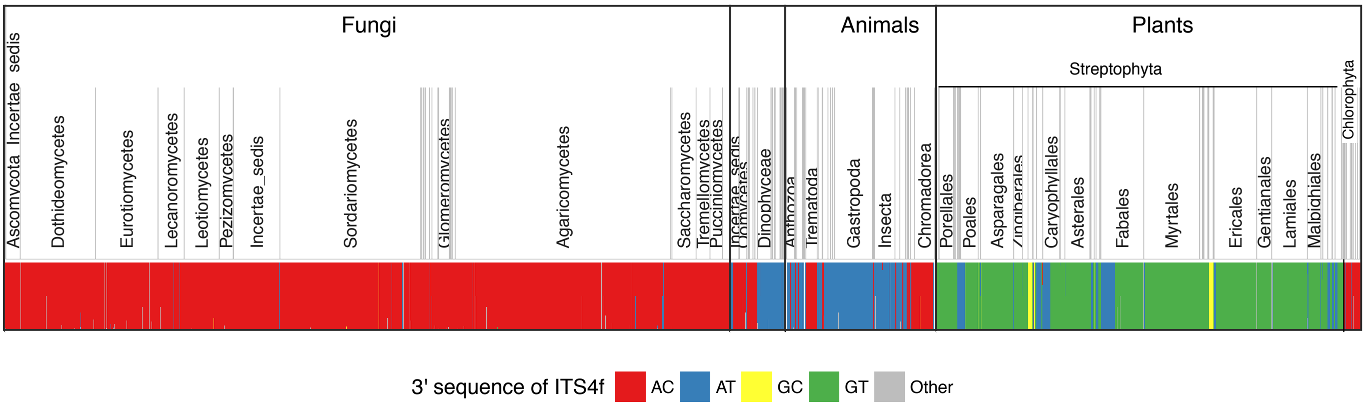

Figure S2: Occurrence of the 2 bp sequence matching the 3' end of the ITS4f/ITS4f2 primers across kingdoms based on LSU sequences from GenBank. The relative dinucleotide frequencies were calculated for each of the 37,268 species and are represented as stacked vertical lines. Classes (resp. plant orders) are delimited by vertical grey lines.

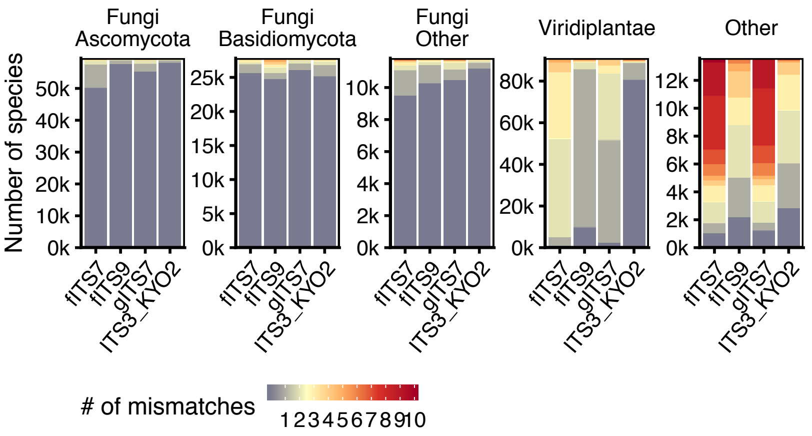

Figure S3: Comparison of primer mismatches to 5.8S sequences found on GenBank for the primers fITS7, fITS9, gITS7 (Ihrmark et al., 2012) and ITS3-KYO2 (Toju et al., 2012).

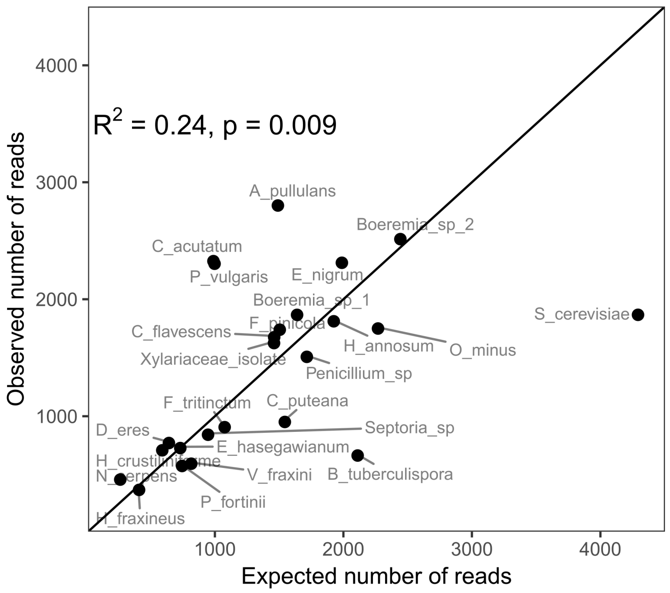

Figure S4: Expected vs. observed read numbers for each species in the even mock community, amplified with the ITS4f reverse primer. The read numbers were scaled to the smallest sample size (33,943). The expected frequencies are based on the results of the rDNA quantification by qPCR. The adjusted r-squared and the p-value of the linear model are shown in the graph. The line has a slope of 1, illustrating a perfect correlation.

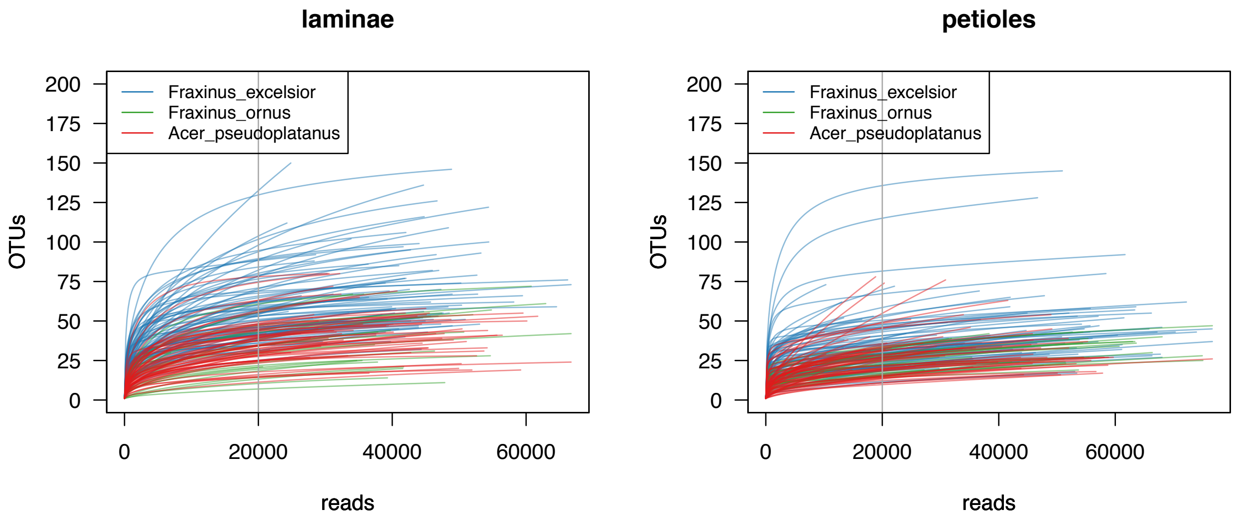

Figure S5: Rarefaction curves for all lamina and petiole samples. The vertical line illustrates the total number of sequences in the scaled samples (20,000) used in many figures of the paper.

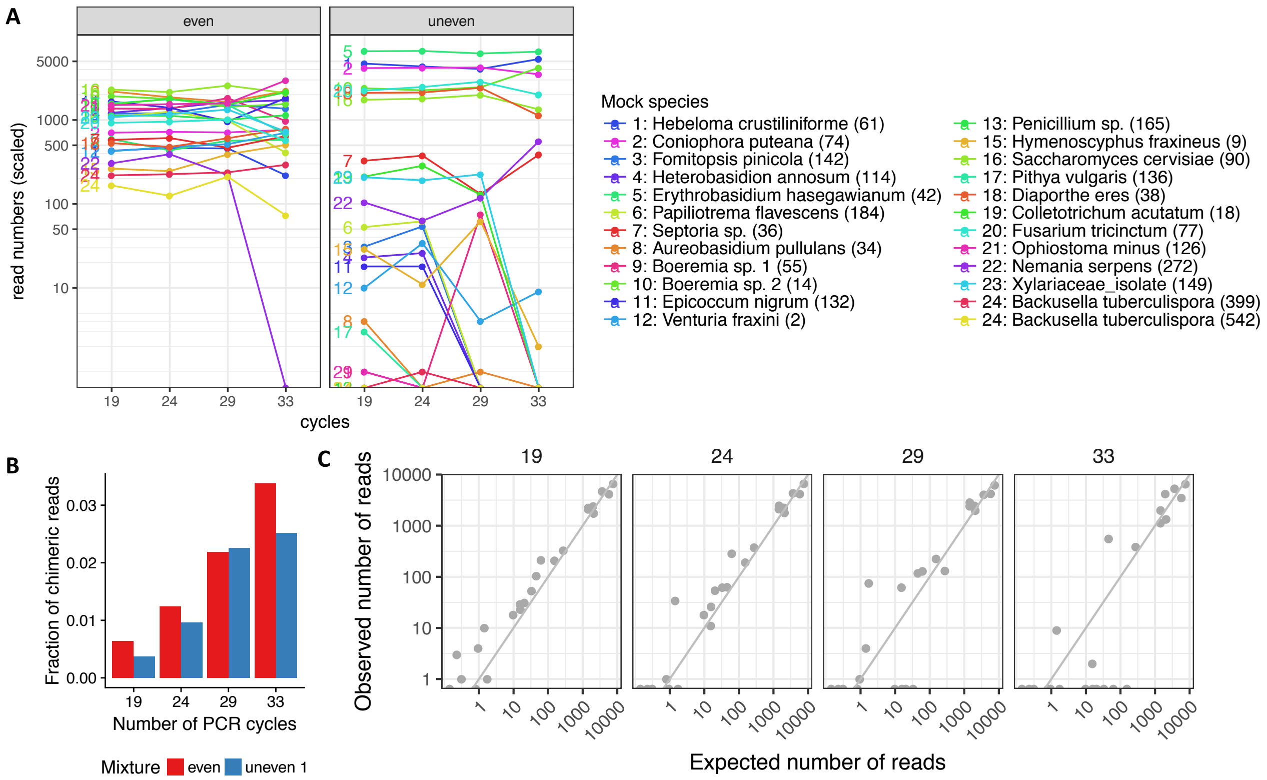

Figure S6: Influence of different PCR cycle numbers on the amplification of the mock communities. **(A)** Number of scaled reads per OTU in the even (left) and uneven (right) mock communities. For each species, exactly one OTU was found (OTU identifiers in parentheses) except for *B. tuberculispora* (24), which was matched by two OTUs. **(B)** Proportion of chimeric reads depending on the number of PCR cycles in the even and uneven mixtures. **(C)** Comparison of expected vs. observed read numbers for each species in the mock community, amplified with different cycle numbers (on top of the diagrams). The expected frequencies were corrected by the qPCR measurements. The read numbers per sample were scaled to a total of 20,000 reads.

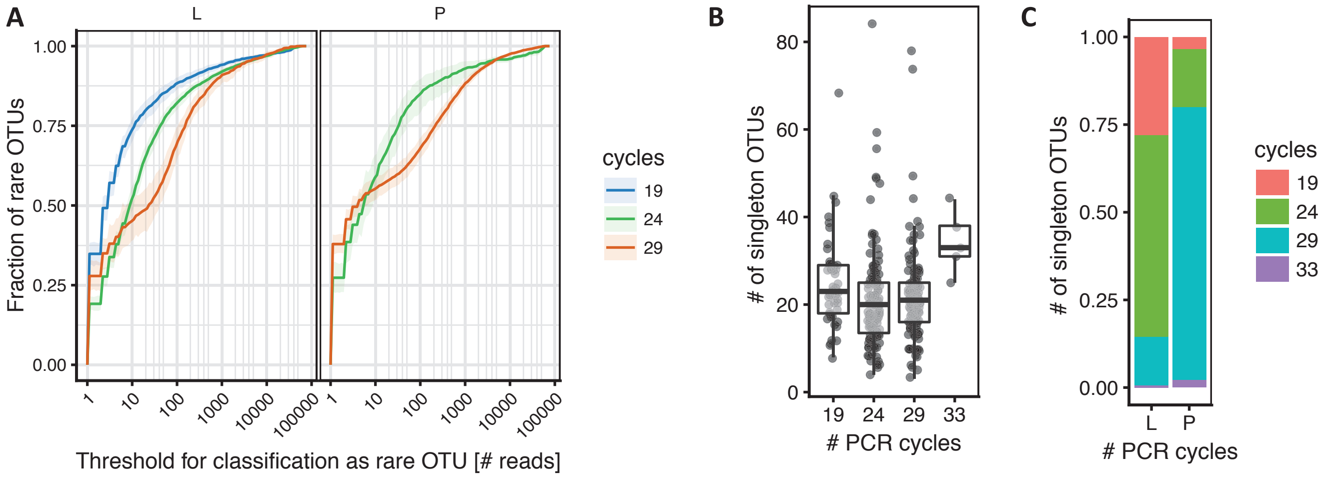

Figure S7: Biases due to the number of PCR cycles. **(A)** Effect of amplification cycle number on the amount of rare OTUs. The fraction of rare OTUs was calculated for all rarefied lamina (L) and petiole (P) samples at different thresholds (x-axis), below which an OTU is classified as rare. The lines denote the mean fraction of rare OTUs and the 99% confidence intervals are shown as colored area, as obtained from a nonparametric bootstrap (*smean.cl.boot* function from the *Hmisc* package in R). Petiole samples amplified with 19 or 33 cycles were not included due to their low numbers (< 5). **(B)** Number of OTUs with only one sequence (singletons) in the dataset of fungal reads scaled to an equal library size of 20,000 reads. **(C)** Number of lamina and petiole samples amplified with 19, 24, 29 and 33 PCR cycles.

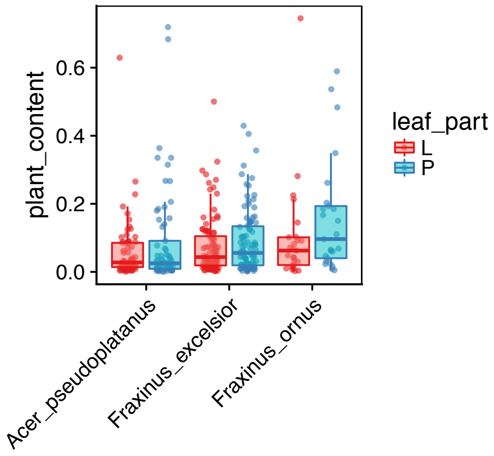

Figure S8: Relative frequencies of plant amplicon within samples amplified with the primers ITS3-KYO2 and ITS4f.

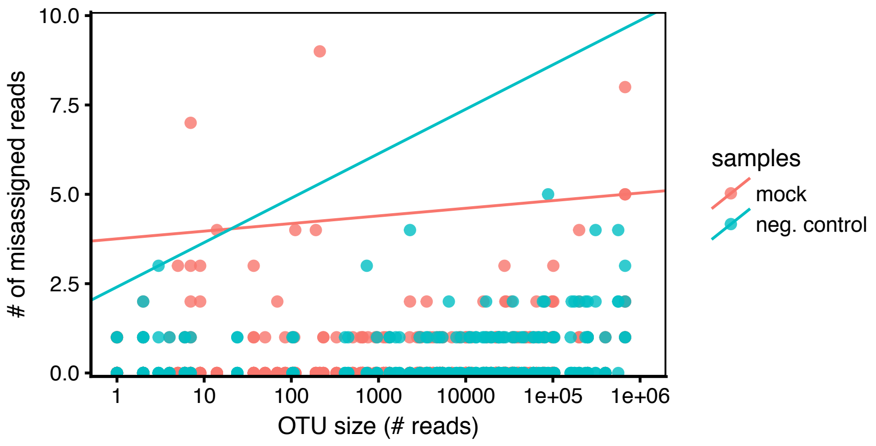

Figure S9: Number of putatively misassigned sequences in the mock communities and negative controls in comparison to total OTU size. The actual mock species were removed from the mock samples before this analysis. The misassigned read numbers were calculated in relation to a sample size of 20,000 (the size of the scaled samples). In addition, the lines from a quantile regression (99.5%) with log10-transformed OTU sizes as independent variable are shown for each group of samples. Since the negative controls have no actual sample size per definition, the mean of all sample sizes was used to calculate the rate of misassigned sequences.

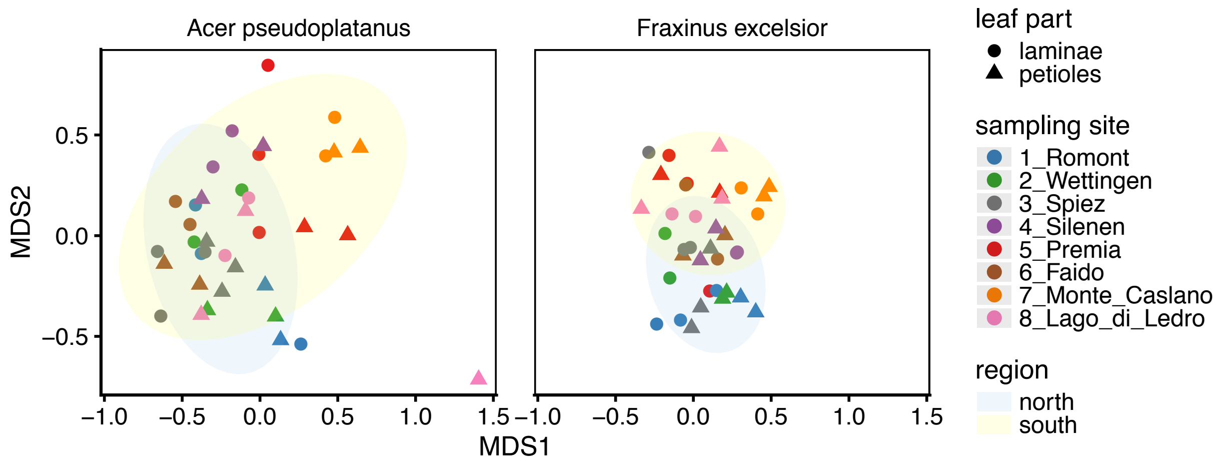

Figure S10: Non-metric multidimensional scaling (NMDS) plot based on isolated fungal morphotypes. Due to the low amount of two leaf pieces per tree and leaf part, data from eight trees (two groups) located in proximity to each other was combined (except for sites 7 and 8, where trees 1-4 and 5-8 were more separated). The points from the two host species are shown in separate panels. The analysis was run with k=3 based on Bray-Curtis distances, and the stress value was 0.22

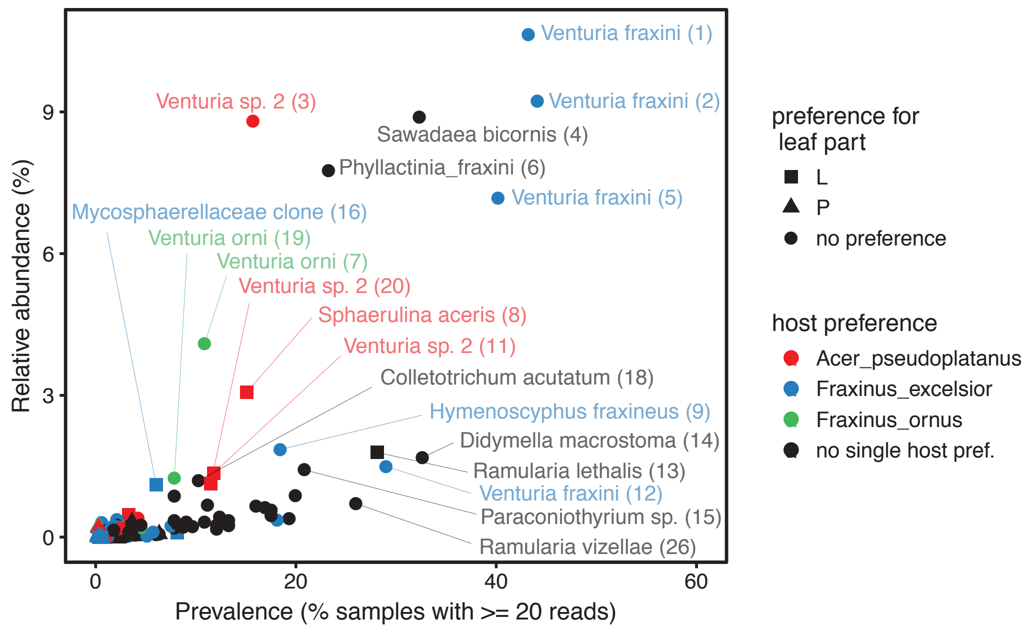

Figure S11: Prevalence versus relative abundance of fungal OTUs. The prevalence was calculated as the percentage of samples with at least 20 rarefied reads for a given OTU. OTUs were colored if 90% of all samples with >= 20 reads belonged to a specific host. Similarly, OTUs with a clear preference for a leaf part are represented as squares or triangles. The most abundant / prevalent OTUs were labeled with their species name and OTU numbers.

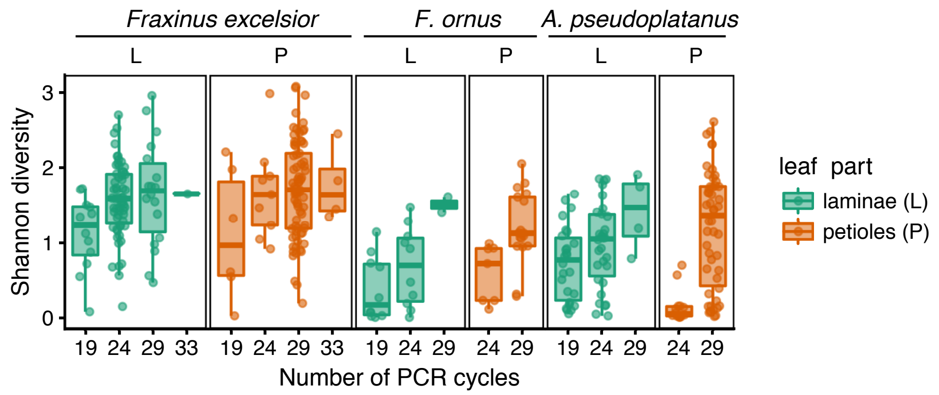

Figure S12: Shannon diversity index vs. the number of PCR cycles in the first amplification step. The index increases with increasing number of cycles regardless of the leaf part (L/P) or host species.

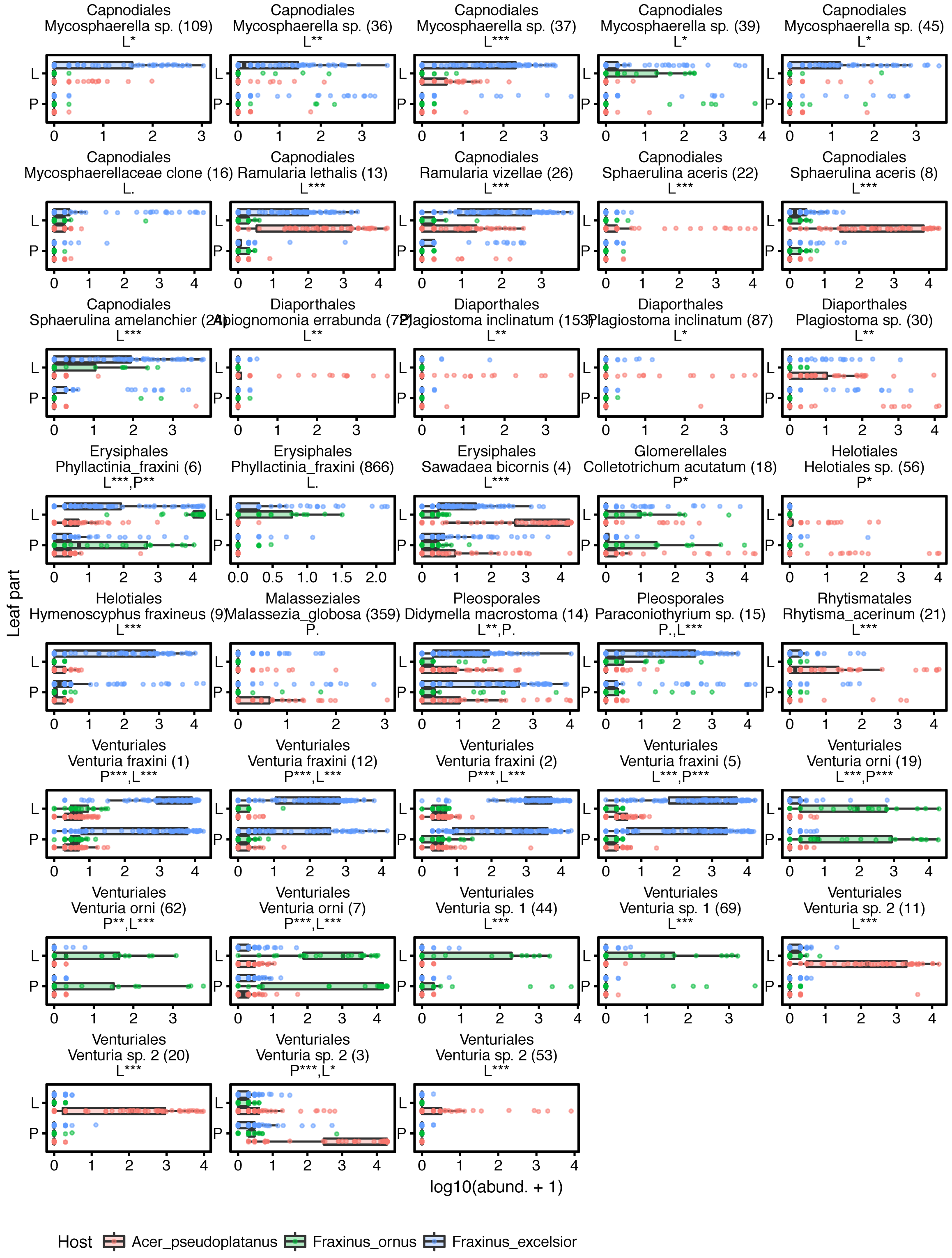

Figure S13: Abundance comparison for OTUs with a differential distribution between the three examined host species. Analyzes were done separately for each leaf part (see **Table S3**). Scaled and log transformed read numbers are shown as box plots with per-sample abundances overlaid as points. OTUs are sorted by parameter estimates, taxa specific for *F. excelsior* appear first, followed by *A. pseudoplatanus* and *F. ornus* specific taxa and/or host combinations. The taxonomic order and species are listed, followed by the OTU identifiers in parentheses. The last line shows for which leaf part (lamina=L and/or petiole=P) significant differences could be found, along with the significance level of the FDR adjusted p-value (* $p < 0.05$, ** $p < 0.01$, *** $p < 0.001$).

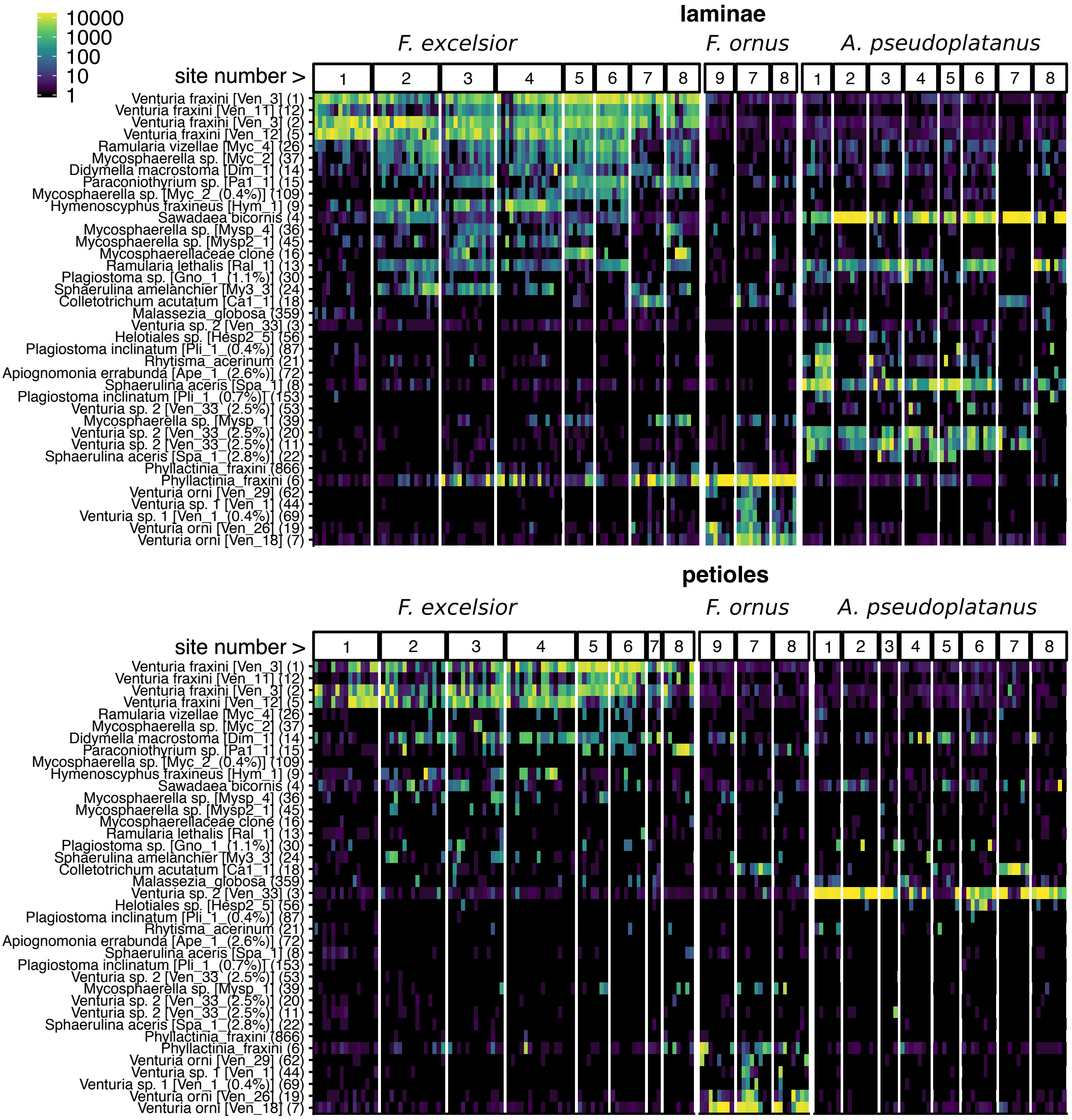

Figure S14: Heatmap of scaled read abundances for OTUs with a differential distribution between the examined host species (see also **Figure S13**). Separate heatmaps are shown for laminae (L) and petioles (P), including all OTUs even if differences were only significant for the other leaf part. The corresponding cultured isolate codes (if any) are written in square brackets along with the % deviation of the OTU sequence from the isolate sequence (if any). The OTU identifiers can be found in parentheses at the end.

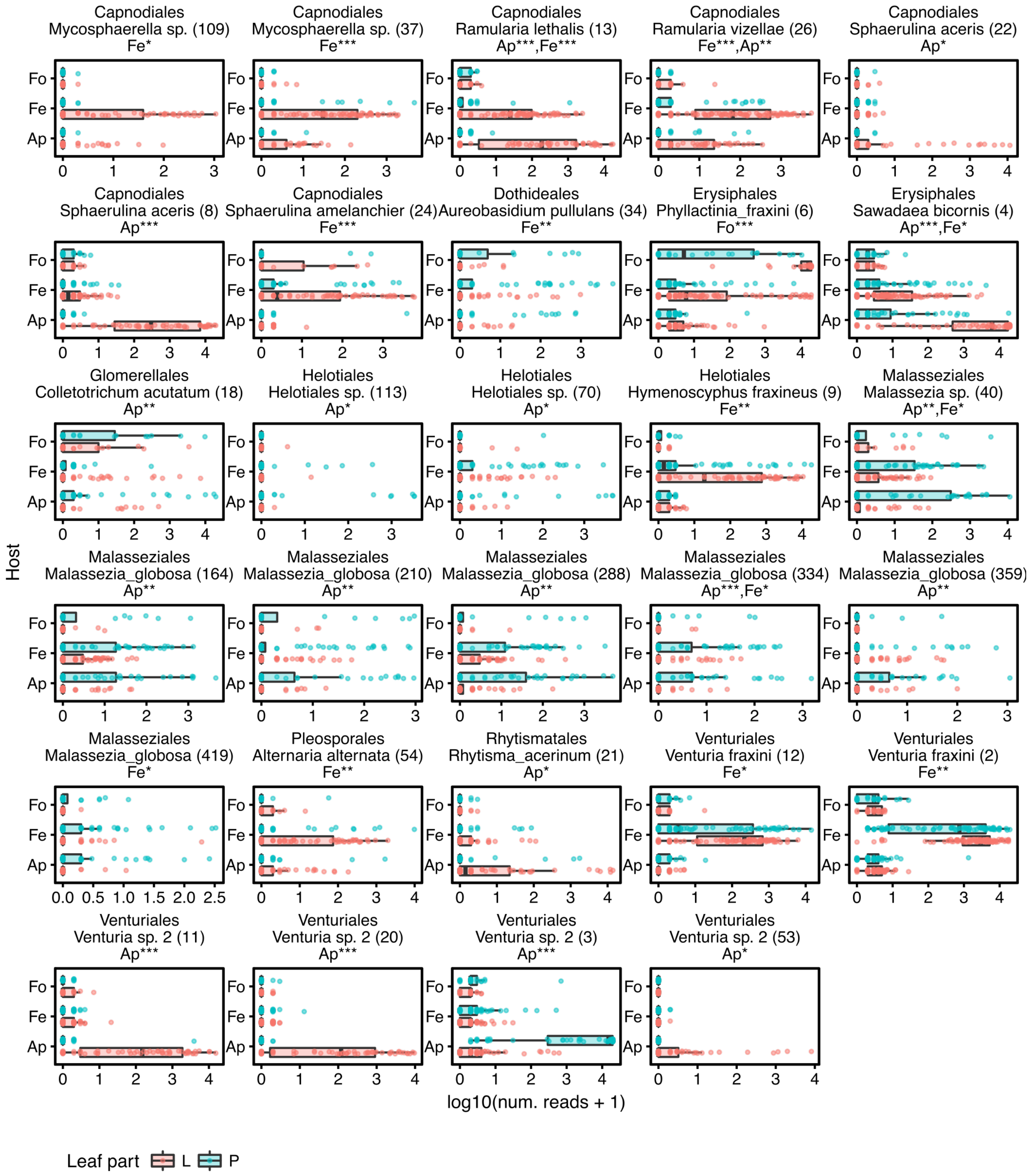

Figure S15: Abundance comparison for OTUs with a significant preference for laminae or petioles. Analyzes were done separately for each host species (see **Table S3**). For which host species the analysis was significant is indicated after the species name (Fe=*F. excelsior*, Fo= *F. ornus*, Ap=*A. pseudoplatanus*) with the significance level as described for **Figure S13**.

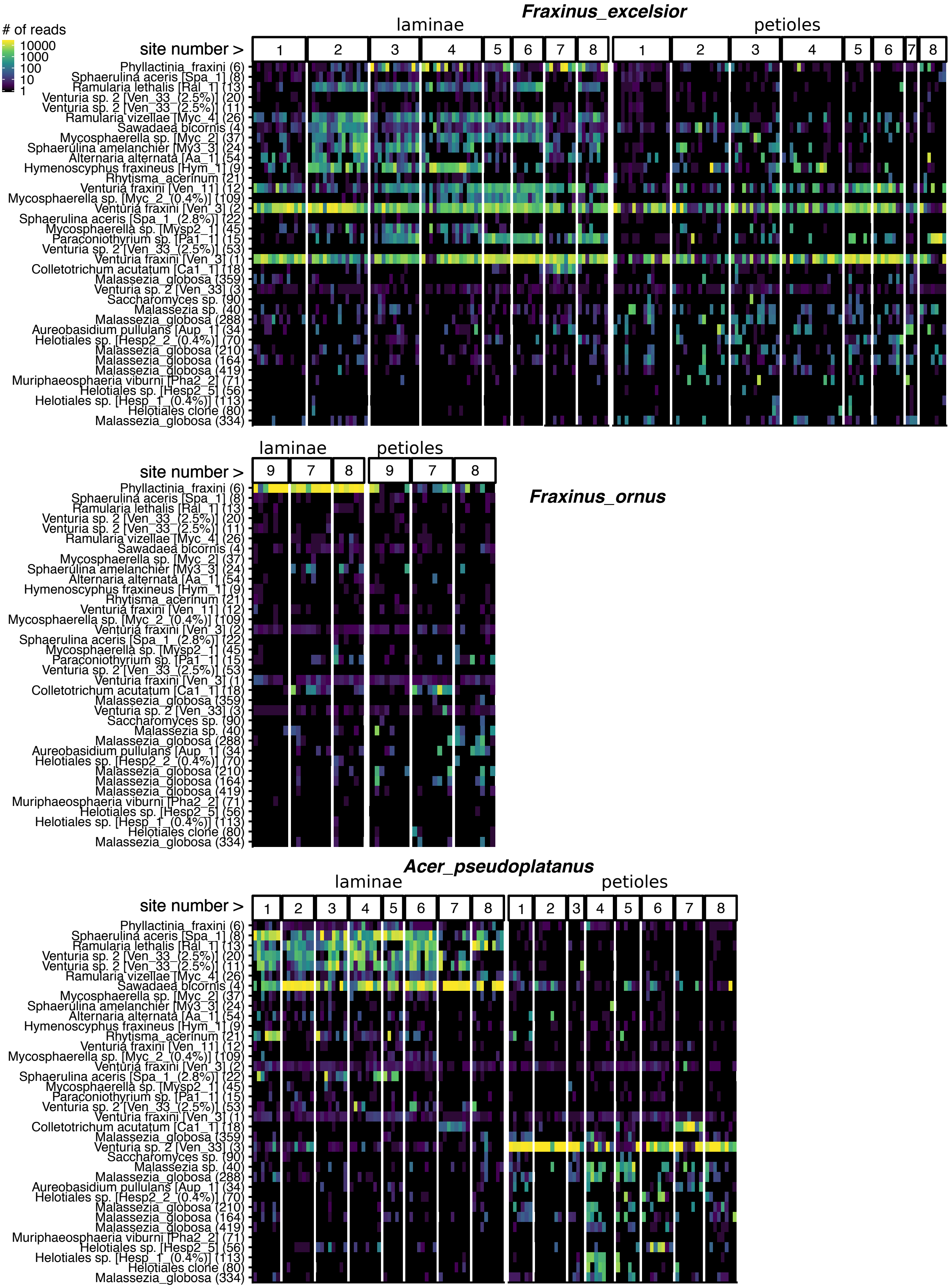

# of reads
10000
1000
100
10
1
Fraxinus_excelsior
laminae
petioles
site number >
1 2 3 4 5 6 7 8
1 2 3 4 5 6 7 8
Phyllactinia_fraxini (6)
Sphaerulina aceris [Spa_1] (8)
Ramularia lethalis [Ral_1] (13)
Venturia sp. 2 [Ven_33_(2.5%)] (20)
Venturia sp. 2 [Ven_33_(2.5%)] (11)
Ramularia vizellae [Myc_4] (26)
Sawadaea bicornis (4)
Mycosphaerella sp. [Myc_2] (37)
Sphaerulina amelanchier [My3_3] (24)
Alternaria alternata [Aa_1] (54)
Hymenoscyphus fraxineus [Hym_1] (9)
Rhytisma_acerinum (21)
Venturia fraxini [Ven_11] (12)
Mycosphaerella sp. [Myc_2_(0.4%)] (109)
Venturia fraxini [Ven_3] (2)
Sphaerulina aceris [Spa_1_(2.8%)] (22)
Mycosphaerella sp. [Mysp2_1] (45)
Paraconiothyrium sp. [Pa1_1] (15)
Venturia sp. 2 [Ven_33_(2.5%)] (53)
Venturia fraxini [Ven_3] (1)
Colletotrichum acutatum [Ca1_1] (18)
Malassezia_globosa (359)
Venturia sp. 2 [Ven_33] (3)
Saccharomyces sp. (90)
Malassezia sp. (40)
Malassezia_globosa (288)
Aureobasidium pullulans [Aup_1] (34)
Helotiales sp. [Hesp2_2_(0.4%)] (70)
Malassezia_globosa (210)
Malassezia_globosa (164)
Malassezia_globosa (419)
Muriphaeosphaeria viburni [Pha2_2] (71)
Helotiales sp. [Hesp2_5] (56)
Helotiales sp. [Hesp_1_(0.4%)] (113)
Helotiales clone (80)
Malassezia_globosa (334)
laminae
petioles
site number >
9 7 8
9 7 8
Fraxinus_ornus
Acer_pseudoplatanus
laminae
petioles
site number >
1 2 3 4 5 6 7 8
1 2 3 4 5 6 7 8

Figure S16 (previous page): Heatmap of scaled read abundances for OTUs with a significant preference for laminae or petioles (see also **Figure S15**). Separate heatmaps are shown for each host species, including all OTUs even if differences were only significant for one host. The corresponding cultured isolate codes (if any) are written in square brackets along with the % deviation of the OTU sequence from the isolate sequence (if any). The OTU identifiers can be found in parentheses at the end.

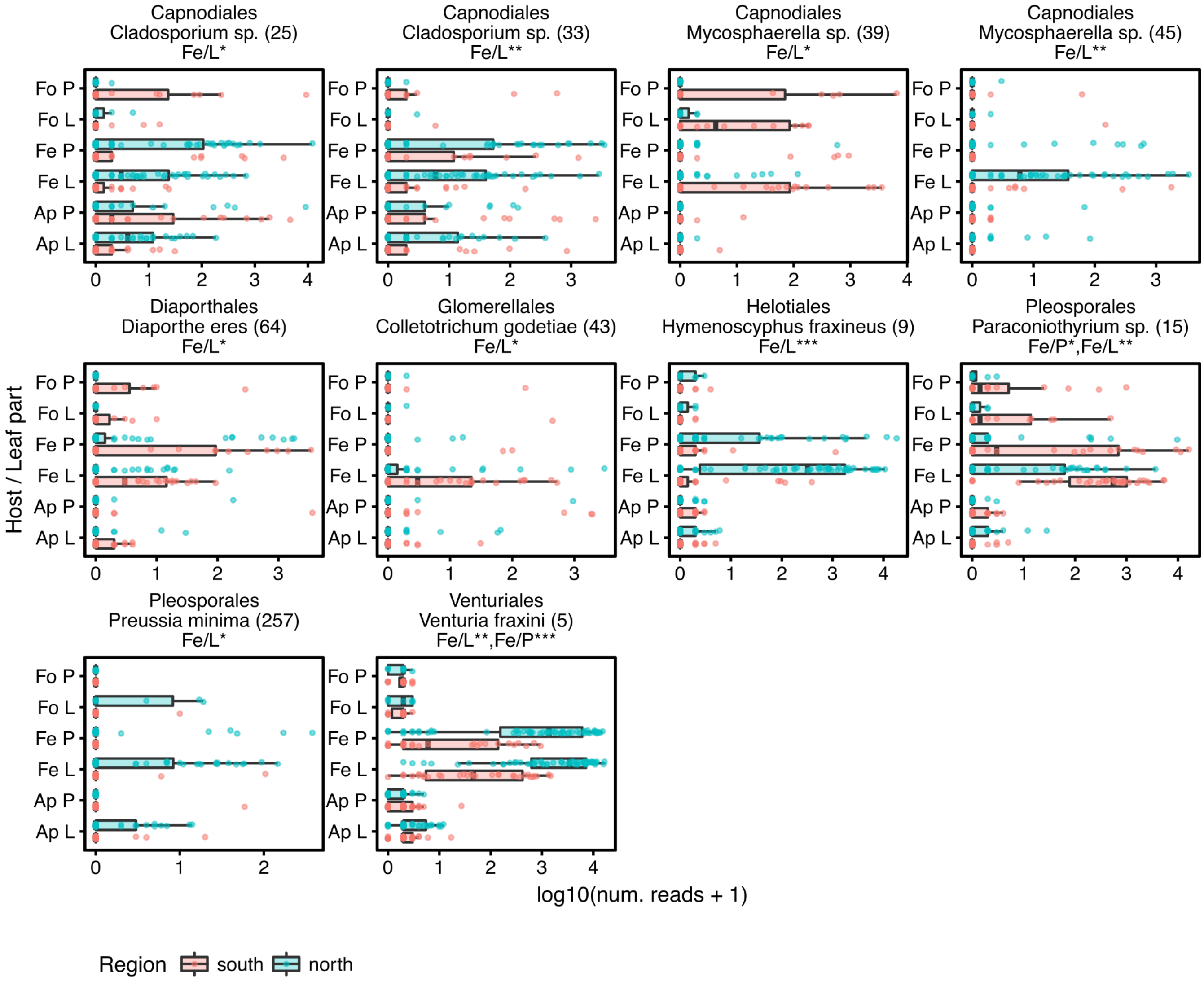

Figure S17: Abundance comparison for OTUs with a significant* preference for the north or south side of the Alps. Boxplots are shown for each host / leaf part combination (Fe=*F. excelsior*, Fo= *F. ornus*, Ap=*A. pseudoplatanus*). For which combination the analysis was significant is indicated after the species name with the significance level as described for **Figure S13**.
* Note that the p-values were not adjusted for multiple testing as described in the paper.

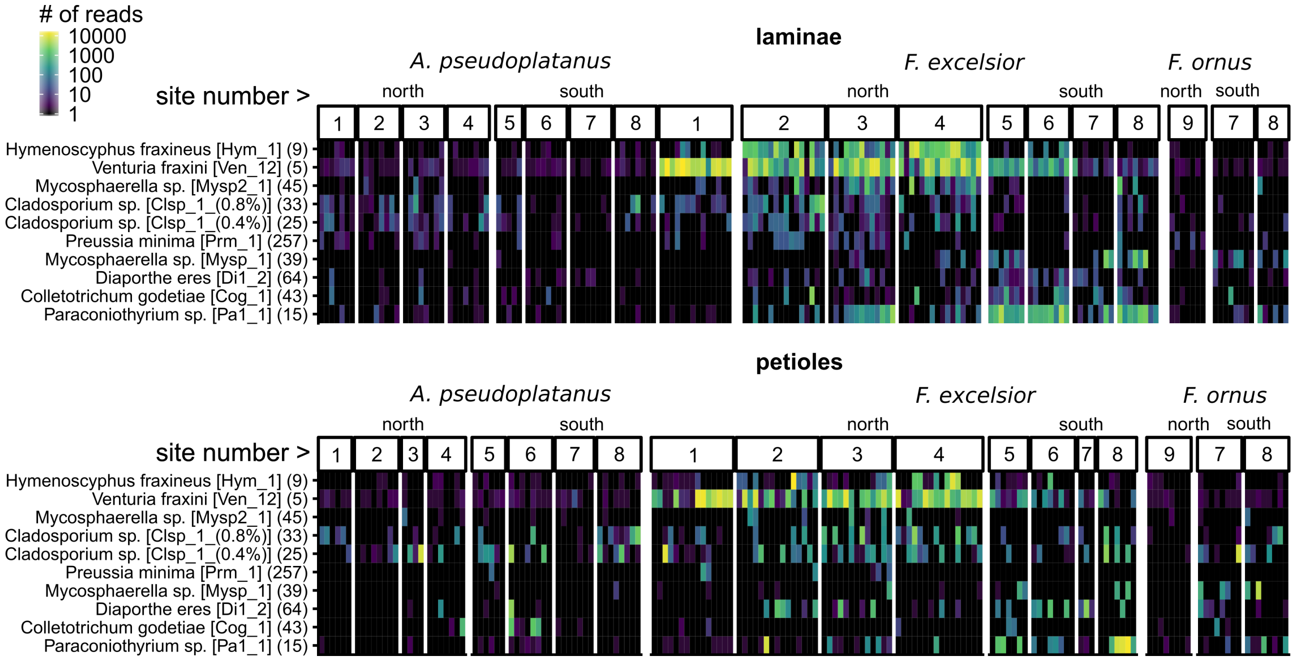

Figure S18: Heatmap of scaled read abundances for OTUs with a significant* preference for north or south side of the Alps (see also **Figure S16**). Separate heatmaps are shown for each leaf part, including all OTUs even if differences were only significant for one leaf part. The corresponding cultured isolate codes (if any) are written in square brackets along with the % deviation of the OTU sequence from the isolate sequence (if any). The OTU identifiers can be found in parentheses at the end. *Note that the p-values were not adjusted for multiple testing as described in the paper.

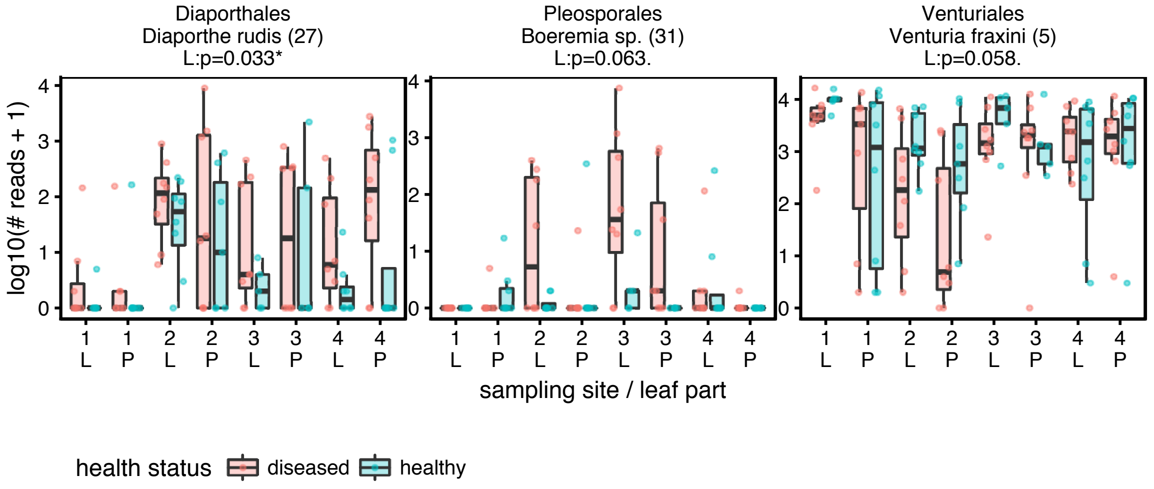

Figure S19: Abundance distribution for the three OTUs with the strongest preference for healthy or diseased *F. excelsior* trees (all found in leaflet communities). Boxplots of scaled and log-transformed read numbers are shown for each sampling site (1-4) north of the Alps and leaf part (L=laminae, P=petioles), overlaid with points representing the counts for each tree. The p-values were not adjusted for multiple testing.

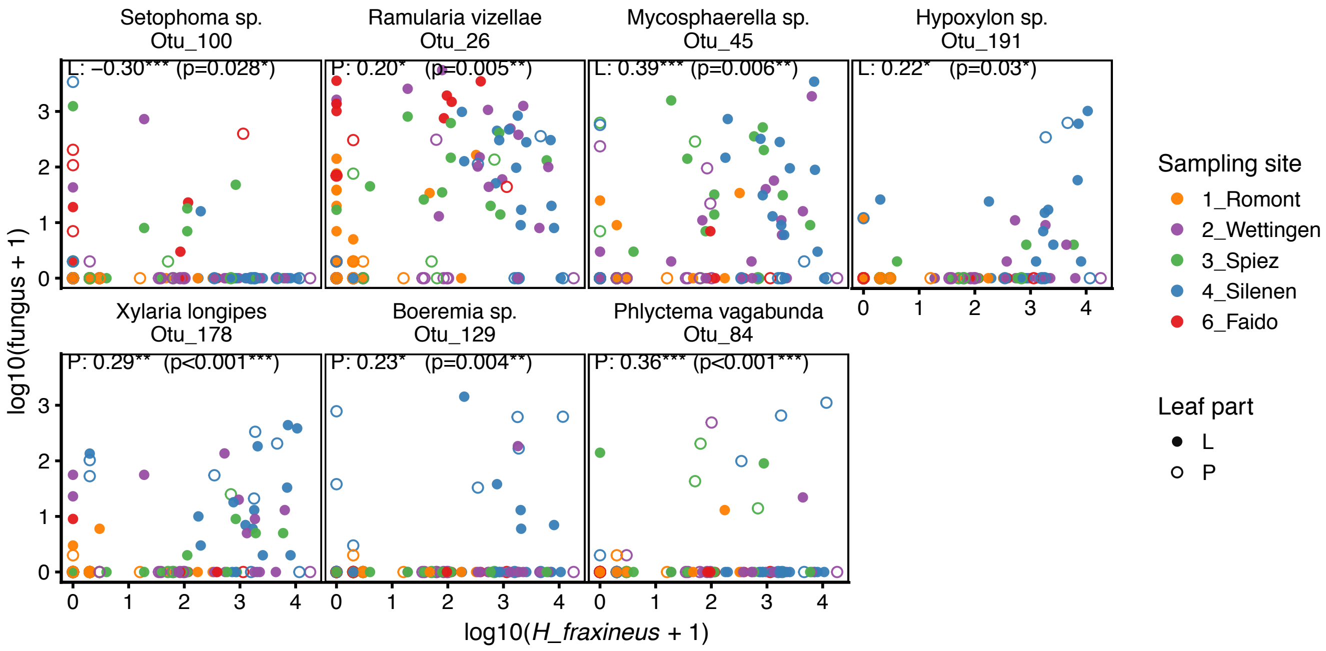

Figure S20: Comparison of OTU frequencies of leaf fungi with negative or positive correlations to frequency of the pathogen *H. fraxineus*. The scaled and log-transformed read frequencies are compared for laminae (L, filled circles) and petioles (P, empty circles). The correlations were determined separately for laminae and petioles and additionally validated using a linear mixed model. OTUs are shown if the association was significant in both analyzes for at least one leaf part. The correlation coefficients for significant associations can be found on top of the panels along with the significance level of the SPARCC pseudo p-value (* p < 0.05, ** p < 0.01, *** p < 0.001), and the p-value of the linear mixed model in parentheses. The OTUs are sorted by the mean of the correlation coefficients.

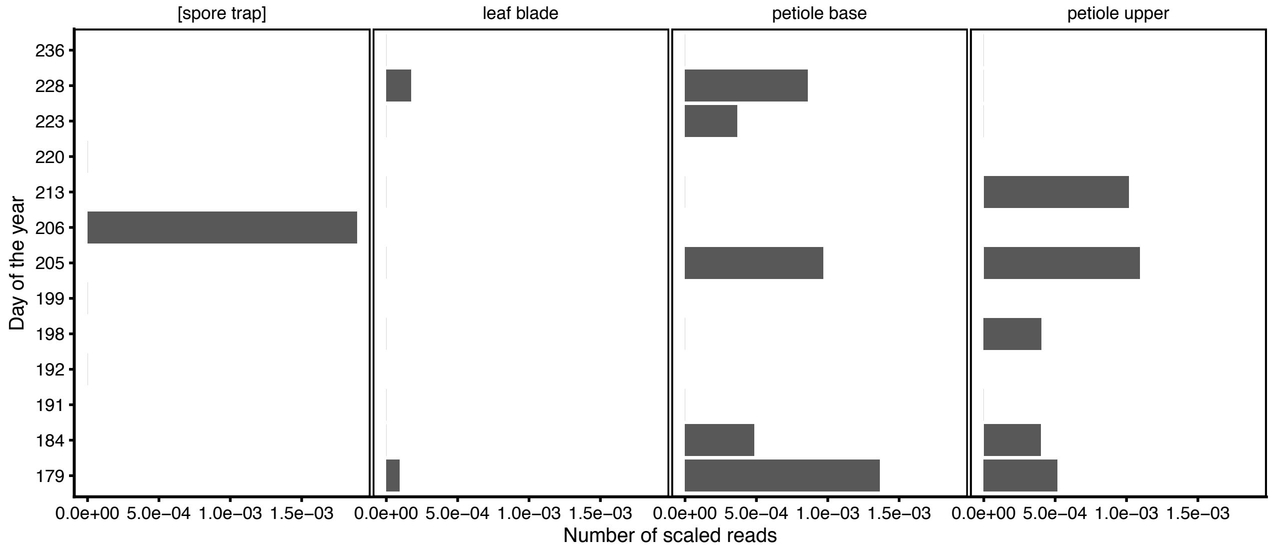

Figure S21: Occurrence of *Malassezia* spp. in the dataset from Cross et al. (2017) (See **Supplementary Methods 1.10**). The relative frequencies are shown dependent on the day of the year. The maximum number of *Malassezia* reads in one sample was 26. Almost all of the sequences belonged to *M. restricta*.

5 Supplementary Tables

Table S1: Detailed overview of sampling sites. The values for the climatic variables (temperature, precipitation) were inferred from data from nearby measuring stations. For Switzerland, the data were obtained from MeteoSwiss. The mean values for the years 1981-2013 (or shorter) were inferred from measurements of nearby stations by linear interpolation based on their elevations. For the Premia site (5), mean values for the years 2002-2010 were calculated from data by the Regional Agency for the Protection of the Environment of Piedmont (https://www.arpa.piemonte.gov.it/rischinaturali/accesso-ai-dati/annali_meteoidrologici/annali-meteo-idro/banca-dati-meteorologica.html). For the site "Lago di Ledro" (8), annual means from a nearby weather station were obtained from Di Piazza and Eccel (2012) for a nearby station (1981-2010), and monthly means were interpolated from measurements of the Rovereto and Ronzo stations (https://climatlas.fbk.eu/view/diagrammi, 1981-2010).

Forest site	Romont	Wettingen	Spiez	Silenen	Premia	Faido	Monte Caslano	Lago di Ledro
Site number	1	2	3	4	5	6	7	8
Country	CH	CH	CH	CH	IT	CH	CH	IT
Coordinates	47.1800 N 7.3076 E	47.4734 N 8.3370 E	46.6859 N 7.6530 E	46.7947 N 8.6642 E	46.2767 N 8.3509 E	46.4719 N 8.8075 E	45.9613 N 8.8815 E	45.8781 N 10.7633 E, 45.8602 N 10.7494 E
Elevation (m)	954	535	609	497	734	680	441 - 463	681 / 713
Collection date	28 Aug	28 Aug	31 Aug	26 Aug	31 Aug	26 Aug	05 Sep	05 Sep
Isolation date	29/30 Aug	29/30 Aug	01/02 Sep	27/28 Aug	01/02 Sep	27/28 Aug	06/07 Sep	04 Sep
Number of healthy *F. excelsior* trees	16	16	12[1]	16	16	16	16	16
Number of diseased *F. excelsior* trees	16	16	16	16				
Number of *F. ornus* trees [2]							16	16
Number of *A. pseudoplatanus* trees	16	16	16	16	16	16	16	16
Nearby weather stations[3]	BIL / CHA (t/p), MGL (p)	LAE / NABLAE (p/t) BAD / OTE (p)	WIS / HON (p) THU / INT / ABO (t)	ALT / GOS / ANT	Premia	COM / FAI / PIO	LUG / THIARO / GEN	Bezzecca (t/p), Rovereto / Ronzo (t)
Mean temperature January [°C]	-0.5	0.1	-0.4	0.9	0.7	1	2.8	1
July [°C]	16.7	18.9	18.1	18.2	19.3	18.8	21.1	20.4
Annual mean [°C]	7.7	9.4	8.7	9.2	9.7	9.8	11.6	10.5
Annual precipitation [mm]	1303	1011	1423	1221	1209	1467	1637	1253
Days with rain (> 1 mm)	135	127	139	132	97	106	99	102

[1] This place was heavily affected by ash dieback. Therefore, it was not possible to find enough healthy-looking trees.
[2] Ibrahim et al. (2017)
[3] Names/codes of the weather stations used for interpolation. t: temperature interpolated, p: precipitation interpolated

Table S2: Overview of the PERMANOVA analyzes

Factor	Subsets	Formula (right hand side)	Stratum
Host species and sampling site	Leaf part: lamina (L), petiole (P)	`~ (host + plot + cycles)^2`	
region (north / south side of the Alps)	Host species, leaf part: Fe/L, Fe/P, Fo/L, Fo/P, ...	`~ region * cycles`	site / health
Health status	Leaf part (*F. excelsior*): Fe/L, Fe/P	`~ health * cycles`	site

Table S3: Overview of the linear mixed models used for finding differentially distributed OTUs. The right hand side of each formula is shown, containing all factors.

Factor	Subsets	Formula (right hand side)
Host species	Leaf part: lamina (L), petiole (P)	`~ host + (1|plot) + (1|cycles)`
Leaf part	Host species: *F. excelsior* (Fe), *F. ornus* (Fo), *A. pseudoplata-nus* (Ap)	`~ leaf_part + (1|plot) + (1|cycles)`
region (north / south)	Host species, leaf part: Fe/L, Fe/P, Fo/L, Fo/P, ...	`~ north_south + (1|plot) + (1|cycles)`
Health status	Leaf part (F. excelsior): Fe/L, Fe/P	`~ health + (1|plot) + (1|cycles)`

Table S4: List of isolates used for the assembly of the mock communities. See also **Table File 4**.

#	phylum	class	order	family	genus	species	isolation source	days in liquid culture	expected frequency (qPCR)[2] mean	std. dev.	accession
1	Basidiomycota	Agaricomycetes	Agaricales	Cortinariaceae	Hebeloma	*Hebeloma crustiliniforme*	root from mixed *Picea mariana*, *P. glauca*, *Pinus contorta*, *Populus* woods (UAMH 5460)	21.0	1.7E-02	2.6E-03	MH931275
2	Basidiomycota	Agaricomycetes	Boletales	Coniophoraceae	Coniophora	*Coniophora puteana*	dead wood	13.0	4.5E-02	1.5E-03	MH931268
3	Basidiomycota	Agaricomycetes	Polyporales	Coriolaceae	Fomitopsis	*Fomitopsis pinicola*	dead wood	12.0	4.4E-02	2.1E-03	MH931272
4	Basidiomycota	Agaricomycetes	Russulales	Bondarzewiaceae	Heterobasidion	*Heterobasidion annosum*	fruiting body	11.0	5.7E-02	6.8E-03	MH931274
5	Basidiomycota	Cystobasidiomycetes	Erythrobasidiales	Erythrobasidiaceae	Erythrobasidium	*Erythrobasidium hasegawianum*	*epiphytic on Pinus sylvestris*	3.9	2.1E-02	1.5E-03	MH931270
6	Basidiomycota	Tremellomycetes	Tremellales	Rhynchogastremataceae	Papiliotrema	*Papiliotrema flavescens*	*F. excelsior*leaves (epiphytic)	3.9	4.3E-02	3.6E-03	MH931267
7	Ascomycota	Dothideomycetes	Capnodiales	Mycosphaerellaceae	Septoria	*Septoria sp.*	*F. excelsior leaves*	11.0	2.8E-02	2.3E-03	MH931283
8	Ascomycota	Dothideomycetes	Dothideales	Dothioraceae	Aureobasidium	*Aureobasidium pullulans*	*F. excelsior leaves*	3.9	4.4E-02	4.8E-03	MH931262
9	Ascomycota	Dothideomycetes	Pleosporales	Didymellaceae	Boeremia	*Boeremia sp. 1*	*F. excelsior leaves*	6.0	4.8E-02	1.0E-03	MH931264
10	Ascomycota	Dothideomycetes	Pleosporales	Didymellaceae	Boeremia	*Boeremia sp. 2*[1]	*F. excelsior leaves*	6.0	7.2E-02	1.7E-03	MH931265
11	Ascomycota	Dothideomycetes	Pleosporales	Didymellaceae	Epicoccum	*Epicoccum nigrum*	Epiphytic	3.0	5.8E-02	4.5E-03	MH931271
12	Ascomycota	Dothideomycetes	Venturiales	Venturiaceae	Venturia	*Venturia fraxini*	*F. excelsior leaves*	11.0	2.4E-02	1.2E-03	MH931284
13	Ascomycota	Eurotiomycetes	Eurotiales	Aspergillaceae	Penicillium	*Penicillium sp.*	*F. excelsior* leaves (epiphytic)	6.0	5.0E-02	4.4E-03	MH931281
14	Ascomycota	Leotiomycetes	Helotiales		Phialocephala	*Phialocephala fortinii*	*Picea abies* fine root (UAMH 11012)	13.0	2.2E-02	1.1E-03	MH931279
15	Ascomycota	Leotiomycetes	Helotiales	Helotiaceae	Hymenoscyphus	*Hymenoscyphus fraxineus*	*F. excelsior* necrosis, Vienna	25.0	1.2E-02	2.7E-04	MH931276
16	Ascomycota	Saccharomycetes	Saccharomycetales	Saccharomycetaceae	Saccharomyces	*Saccharomyces cervisiae*	commercial baker's yeast	3.9	1.3E-01	5.6E-03	MH931282
17	Ascomycota	Pezizomycetes	Pezizales	Sarcoscyphaceae	Pithya	*Pithya vulgaris*	*Abies balsamea* needle (endophytic)	13.0	2.9E-02	1.9E-03	MH931280
18	Ascomycota	Sordariomycetes	Diaporthales	Diaporthaceae	Diaporthe	*Diaporthe eres*	*F. excelsior leaves*	6.0	1.9E-02	1.6E-03	MH931269
19	Ascomycota	Sordariomycetes	Glomerellales	Glomerellaceae	Colletotrichum	*Colletotrichum acutatum*	*F. excelsior leaves*	4.0	2.9E-02	3.7E-03	MH931266
20	Ascomycota	Sordariomycetes	Hypocreales	Nectriaceae	Fusarium	*Fusarium tricinctum*	*F. excelsior leaves*	4.0	3.2E-02	1.6E-03	MH931273
21	Ascomycota	Sordariomycetes	Ophiostomatales	Ophiostomataceae	Ophiostoma	*Ophiostoma minus*	*Pinus sylvestris* wood (stem)	6.0	6.7E-02	3.9E-03	MH931278
22	Ascomycota	Sordariomycetes	Xylariales	Xylariaceae	Nemania	*Nemania serpens*	*F. excelsior leaves*	13.0	7.6E-03	1.2E-04	MH931277
23	Ascomycota	Sordariomycetes	Xylariales	Xylariaceae		*Xylariaceae isolate*	*F. excelsior leaves*	13.0	4.3E-02	2.5E-03	MH931285
24	(Zygomycota)		Mucorales	Backusellaceae	Backusella	*Backusella tuberculispora*	forest soil	4.0	6.2E-02	2.6E-03	MH931263

[1] Similar to sp. 1, ITS2 sequence differs by 1 substitution and 2 InDels,

[2] Expected relative amount of each species in the even mock community as calculated from the qPCR results (N0)

Table S5: Overview of fungal groups with mismatches within the last four 3’ bases of ITS4f

		# Mismatches		
		ITS4f	ITS4f2	ITS4
ITS4 sequence (reverse complement)	<GCATATCAATAAGCGGAGGA			
ITS4f sequence (rev.)	<ACTTAAGCATATYAATAAGCG			
ITS4f2 sequence (rev.)	<AYTTAAGCATATYAATAAGCG			
ITS4-Fun (Taylor et al., 2016) (rev.)	<AYTTAAGCATATCAATAAGCGGAGG			
**Most fungi**	**ACTTAAGCATATCAATAAGCGGAGGA**			
Peltigeraceae	.T........................	1*	0	0
Kickxellomycota	.T..........T.............	1*	0	1
Entomophthorales	.T..........T.............	1*	0	1
some *Tulasnella* spp.	.T..........T.T.C.........	3*	2	3
Uncultured *Mortierella* sp.[1]	.T..........T.............	1*	0	1
Xylaria cubensis	.T.G......C.T.G.C....A....	6*	5	5
Microsporidia	.A..........G.G...AG......	5*	5*	4
GS19 sp. (Tedersoo et al., 2017)	.T........................	1*	0	
Candida sp.[2]	..AA...........W......G..G	4-5	4-5	3-4
Zoopagomycota / *Syncephalis sp*	.T....	1*?	0?	?
1.1.1.1.1 Unidentified Fungi sp. (SH216684.07FU)	.T....	1*?	0?	?
Some *Claroideoglomus claroideum/luteum*[3]	.-....AT...----.....-.......			
Some *Claroideoglomus etunicatum*[3]	.-----------------..-.......			

* One mismatch within last two 3’ bases

[1] All sequences from this groups belong to uncultured *Mortierella* spp., but from different studies (Hedh et al., 2008; Taylor et al., 2013; Timling et al., 2014) and forming a distinct related group (UNITE species hypotheses SH02366[4-7].07FU)

[2] Apparently marine yeasts classified as *Bandonia marina* (Genbank PopSet 663440448), but BLAST suggests *Candida* sp., the sequence of the *B. marina* type (NR_144778) is different.

[3] Sequences from VanKuren Nicholas W. et al. (2013), who described a long and a short ITS variant within the mentioned *Claroideoglomus* species, whereby the short variant has InDels at the end of ITS2 (and also several LSU regions). However, these variants co-occurred in the same genome as confirmed by FISH, therefore part of the LSU sequences should always be matched by the primers.

Table S6: Selective OTU amplification by the ITS4f/ITS4f2 primers in the pond sediment sample. The read counts (normalized) are shown for the different reverse primers, additionally categorized depending on the OTU sequences encountered at the site corresponding to the 3' end of ITS4f/ITS4f2 (see also **Figure S1**). The read numbers [n*] indicate the number of 3’ mismatches between the ITS4f/ITS4f2 primers and OTU sequences, which are shown on top. 'x reduction' indicates the factor by which read numbers were reduced in relation to the ITS4 amplicon.

	**GTTT** *plants*			**ATTT** *animals, protists, some plants, few fungi*			**ACTT** *fungi, some protists, animals & green algae*	
**Reverse primer**		# reads	x reduction		# reads	x reduction	# reads	x reduction
ITS4		8266			6136		103704	
ITS4f	[2*]	9	918 x	[1*]	24	256 x	104263	1.0 x
ITS4f2	[1*]	21	394 x		2883	2.1 x	104266	1.0 x

Table S7: Overview of the concentrations (pg μl^{-1}) of the uneven (#1) mock communities used to match the approximate fungal gDNA concentration in samples amplified with the same number of PCR cycles. They were estimated based on qPCR quantifications. In addition, the expected number of ITS template molecules per 4.5 µl of mock gDNA (amount used for amplification in total) is shown for a hypothetical species with a frequency (m/m) of 0.1% in the mix. For the exact calculation see **Table File 4** (workbook 'ITS_content_calc'). The final concentrations of the indexed and purified PCR products of the mock community (last two columns) were comparable with the leaf sample concentrations, indicating that the dilutions were appropriate.

# of cycles	gDNA conc. [pg μl^{-1}]	# of ITS molecules at 0.1% freq.	conc. after indexing [ng μl^{-1}]	
			even	uneven
19	34.40	286.9	12.0	10.7
24	3.29	27.4	32.2	21.8
29	0.31	2.6	34.9	53.0

Table S8: Statistical comparison of Shannon alpha diversity between fungal leaf communities north and south of the Alps. The p-values for the linear mixed models were determined using a chi-squared test implemented in the Anova function of the *car* package. The p-values were additionally adjusted for multiple testing using the Benjamini-Hochberg method. The sampling site and the number of PCR cycles were included as random effects.

Host	Leaf part	Chisq	Df	Pr	q
F. excelsior	L	0.072	1	0.788	0.938
	P	0.006	1	0.938	0.938
F. ornus	L	4.236	1	0.040*	0.193
	P	0.018	1	0.893	0.938
A. pseudoplatanus	L	0.861	1	0.353	0.578
	P	0.044	1	0.834	0.938

Table S9: PERMANOVA results for testing differences in community structure between host species and sampling sites. The analysis was done separately for laminae (L) and petioles (P). The number of PCR cycles (C) was included as variable, and all two-fold interactions are shown below. P-values (Pr) were adjusted for multiple testing.

	Host species (H)			Sampling site (S)			PCR cycles (C)		
	R^2	F	Pr	R^2	F	Pr	R^2	F	Pr
L	0.22	29.9	< 0.001 ***	0.14	5.01	< 0.001 ***	0.02	2.31	< 0.001 ***
P	0.09	9.84	< 0.001 ***	0.09	2.26	< 0.001 ***	0.02	1.73	< 0.001 ***

Interactions and residual R-squared

	H:S			H:C			S:C			Resid.
	R^2	F	Pr	R^2	F	Pr	R^2	F	Pr	R^2
L	0.07	2.27	< 0.001 ***	0.02	1.11	0.188	0.05	1.17	0.054 .	0.48
P	0.06	1.53	< 0.001 ***	0.01	1.1	0.217	0.04	0.69	1.000	0.69

Table S10: PERMANOVA results for testing differences in community structure between the north and south side of the Alps. The analysis was done separately for each host species and leaf part. The number of PCR cycles was included as variable. P-values (Pr) were adjusted for multiple testing. The sampling sites were included as strata.

		North / south			Cycles			North / south : cycles			Residual
		R^2	F	Pr	R^2	F	Pr	R^2	F	Pr	R^2
F. excelsior	L	0.07	6.34	0.004 **	0.05	1.62	< 0.001 ***	0.01	0.6	0.951	0.88
	P	0.03	2.68	0.007 **	0.05	1.57	0.002 **	0.01	0.87	0.813	0.91
F. ornus	L	0.12	2.74	0.399	0.11	1.32	0.399	0.03	0.76	0.657	0.73
	P	0.06	1.37	0.066 .	0.07	1.65	0.018 *	0.03	0.82	0.678	0.84
A. pseudoplatanus	L	0.04	2.37	0.102	0.06	1.91	0.102	0.06	1.96	0.102	0.84
	P	0.02	1.31	0.696	0.02	1.58	0.21	0.01	0.75	0.946	0.94

Table S11: PERMANOVA results for testing differences in community structure between leaves of asymptomatic ("healthy") and symptomatic *F. excelsior* north of the Alps. The analysis was done separately for each leaf part. Apart from health status, the number of PCR cycles and an interaction term were included in the model. P-values (Pr) were adjusted for multiple testing. The sampling sites were included as strata.

F. excelsior	Health			Cycles			Health : cycles			Residual
	R^2	F	Pr	R^2	F	Pr	R^2	F	Pr	R^2
Laminae	0.02	1.12	0.084 .	0.08	1.59	< 0.001 ***	0.03	0.98	0.397	0.87
Petioles	0.02	0.92	0.850	0.07	1.42	0.023 *	0.03	0.65	0.984	0.88

6 Supplementary References

Amend, A. (2014). From Dandruff to Deep-Sea Vents: *Malassezia*-like Fungi Are Ecologically Hyper-diverse. *PLOS Pathogens* 10, e1004277. doi:10.1371/journal.ppat.1004277.

Bakys, R., Vasaitis, R., Barklund, P., Ihrmark, K., and Stenlid, J. (2009a). Investigations concerning the role of *Chalara fraxinea* in declining *Fraxinus excelsior*. *Plant Pathology* 58, 284–292. doi:10.1111/j.1365-3059.2008.01977.x.

Bakys, R., Vasaitis, R., Barklund, P., Thomsen, I. M., and Stenlid, J. (2009b). Occurrence and pathogenicity of fungi in necrotic and non-symptomatic shoots of declining common ash (*Fraxinus excelsior*) in Sweden. *Eur J Forest Res* 128, 51–60. doi:10.1007/s10342-008-0238-2.

Bengtsson-Palme, J., Ryberg, M., Hartmann, M., Branco, S., Wang, Z., Godhe, A., et al. (2013). Improved software detection and extraction of ITS1 and ITS2 from ribosomal ITS sequences of fungi and other eukaryotes for analysis of environmental sequencing data. *Methods in Ecology and Evolution* 4, 914–919. doi:10.1111/2041-210X.12073.

Bolaños, J., León, L. F. D., Ochoa, E., Darias, J., Raja, H. A., Shearer, C. A., et al. (2015). Phylogenetic Diversity of Sponge-Associated Fungi from the Caribbean and the Pacific of Panama and Their In Vitro Effect on Angiotensin and Endothelin Receptors. *Mar Biotechnol* 17, 533–564. doi:10.1007/s10126-015-9634-z.

Brooks, W. C., Paguigan, N. D., Raja, H. A., Moy, F. J., Cech, N. B., Pearce, C. J., et al. (2017). qNMR for profiling the production of fungal secondary metabolites. *Magn Reson Chem* 55, 670–676. doi:10.1002/mrc.4571.

Cleary, M., Nguyen, D., Marčiulynienė, D., Berlin, A., Vasaitis, R., and Stenlid, J. (2016). Friend or foe? Biological and ecological traits of the European ash dieback pathogen *Hymenoscyphus fraxineus* in its native environment. *Scientific Reports* 6, 21895. doi:10.1038/srep21895.

Cross, H., Sønstebø, J. H., Nagy, N. E., Timmermann, V., Solheim, H., Børja, I., et al. (2017). Fungal diversity and seasonal succession in ash leaves infected by the invasive ascomycete *Hymenoscyphus fraxineus*. *New Phytol* 213, 1405–1417. doi:10.1111/nph.14204.

Crous, P. W., Schubert, K., Braun, U., de Hoog, G. S., Hocking, A. D., Shin, H.-D., et al. (2007). Opportunistic, human-pathogenic species in the Herpotrichiellaceae are phenotypically similar to saprobic or phytopathogenic species in the Venturiaceae. *Studies in Mycology* 58, 185–217. doi:10.3114/sim.2007.58.07.

Davydenko, K., Vasaitis, R., Stenlid, J., and Menkis, A. (2013). Fungi in foliage and shoots of *Fraxinus excelsior* in eastern Ukraine: a first report on *Hymenoscyphus pseudoalbidus*. *For. Path.* 43, 462–467. doi:10.1111/efp.12055.

De Cáceres, M., Legendre, P., and Moretti, M. (2010). Improving indicator species analysis by combining groups of sites. *Oikos* 119, 1674–1684. doi:10.1111/j.1600-0706.2010.18334.x.

Edgar, R. (2016a). UCHIME2: improved chimera prediction for amplicon sequencing. *bioRxiv*, 074252. doi:10.1101/074252.

Edgar, R. C. (2016b). UNCROSS: Filtering of high-frequency cross-talk in 16S amplicon reads. *bioRxiv*, 088666. doi:10.1101/088666.

Gohl, D. M., Vangay, P., Garbe, J., MacLean, A., Hauge, A., Becker, A., et al. (2016). Systematic improvement of amplicon marker gene methods for increased accuracy in microbiome studies. *Nature Biotechnology* 34, 942. doi:10.1038/nbt.3601.

Hedh, J., Wallander, H., and Erland, S. (2008). Ectomycorrhizal mycelial species composition in apatite amended and non-amended mesh bags buried in a phosphorus-poor spruce forest. *Mycological Research* 112, 681–688. doi:10.1016/j.mycres.2007.11.008.

Herrera, P., Suárez, J. P., and Kottke, I. (2010). Orchids keep the ascomycetes outside: a highly diverse group of ascomycetes colonizing the velamen of epiphytic orchids from a tropical mountain rainforest in Southern Ecuador. *Mycology* 1, 262–268. doi:10.1080/21501203.2010.526645.

Higginbotham, S. J., Arnold, A. E., Ibañez, A., Spadafora, C., Coley, P. D., and Kursar, T. A. (2013). Bioactivity of Fungal Endophytes as a Function of Endophyte Taxonomy and the Taxonomy and Distribution of Their Host Plants. *PLOS ONE* 8, e73192. doi:10.1371/journal.pone.0073192.

Higgins, K. L., Coley, P. D., Kursar, T. A., and Arnold, A. E. (2011). Culturing and direct PCR suggest prevalent host generalism among diverse fungal endophytes of tropical forest grasses. *Mycologia* 103, 247–260. doi:10.3852/09-158.

Huang, M.-M., Arnheim, N., and Goodman, M. F. (1992). Extension of base mispairs by Taq DNA polymerase: implications for single nucleotide discrimination in PCR. *Nucleic acids research* 20, 4567–4573.

Ibrahim, M., Schlegel, M., and Sieber, T. N. (2016). *Venturia orni* sp. nov., a species distinct from *Venturia fraxini*, living in the leaves of Fraxinus ornus. *Mycol Progress* 15, 1–12. doi:10.1007/s11557-016-1172-1.

Ihrmark, K., Bödeker, I. T. M., Cruz-Martinez, K., Friberg, H., Kubartova, A., Schenck, J., et al. (2012). New primers to amplify the fungal ITS2 region - evaluation by 454-sequencing of artificial and natural communities. *FEMS Microbiology Ecology* 82, 666–677. doi:10.1111/j.1574-6941.2012.01437.x.

James, T. Y., Kauff, F., Schoch, C. L., Matheny, P. B., Hofstetter, V., Cox, C. J., et al. (2006). Reconstructing the early evolution of Fungi using a six-gene phylogeny. *Nature* 443, 818–822. doi:10.1038/nature05110.

Jiang, W., Yang, G., Zhang, C., and Fu, C. (2011). Species composition and molecular analysis of symbiotic fungi in roots of Changnienia amoena (Orchidaceae). *African Journal of Microbiology Research* 5, 222–228.

Katoh, K., and Standley, D. M. (2013). MAFFT Multiple Sequence Alignment Software Version 7: Improvements in Performance and Usability. *Molecular Biology and Evolution* 30, 772–780. doi:10.1093/molbev/mst010.

Kõljalg, U., Nilsson, R. H., Abarenkov, K., Tedersoo, L., Taylor, A. F. S., Bahram, M., et al. (2013). Towards a unified paradigm for sequence-based identification of fungi. *Mol Ecol* 22, 5271–5277. doi:10.1111/mec.12481.

Kosawang, C., Amby, D. B., Bussaban, B., McKinney, L. V., Xu, J., Kjær, E. D., et al. (2017). Fungal communities associated with species of Fraxinus tolerant to ash dieback, and their potential for biological control. *Fungal Biology*. doi:10.1016/j.funbio.2017.11.002.

Kowalski, T., Kraj, W., and Bednarz, B. (2016). Fungi on stems and twigs in initial and advanced stages of dieback of European ash (*Fraxinus excelsior*) in Poland. *Eur J Forest Res* 135, 565–579. doi:10.1007/s10342-016-0955-x.

Kwok, S., Kellogg, D. E., McKinney, N., Spasic, D., Goda, L., Levenson, C., et al. (1990). Effects of primer-template mismatches on the polymerase chain reaction: Human immunodeficiency virus type 1 model studies. *Nucl. Acids Res.* 18, 999–1005. doi:10.1093/nar/18.4.999.

Oksanen, J., Blanchet, F. G., Friendly, M., Kindt, R., Legendre, P., McGlinn, D., et al. (2017). *vegan:*

Community Ecology Package. Available at: https://CRAN.R-project.org/package=vegan.

Olmo-Ruiz, M. D., and Arnold, A. E. (2014). Interannual variation and host affiliations of endophytic fungi associated with ferns at La Selva, Costa Rica. *Mycologia* 106, 8–21. doi:10.3852/13-098.

Olmo-Ruiz, M. D., and Arnold, A. E. (2017). Community structure of fern-affiliated endophytes in three neotropical forests. *Journal of Tropical Ecology* 33, 60–73. doi:10.1017/S0266467416000535.

Osamu Tateno, Hirose, D., Osono, T., and Takeda, H. (2015). Beech cupules share endophytic fungi with leaves and twigs. *Mycoscience* 56, 252–256. doi:10.1016/j.myc.2014.07.005.

Power, M. W., Hopkins, A. J., Chen, J., Bengtsson, S. B., Vasaitis, R., and Cleary, M. R. (2017). European Fraxinus species Introduced into New Zealand Retain Many of their Native Endophytic Fungi. *Baltic Forestry* 23, 74–81.

Rogers, J. D. (1984). Xylaria cubensis and Its Anamorph Xylocoremium flabelliforme, Xylaria allantoidea, and Xylaria poitei in Continental United States. *Mycologia* 76, 912–923. doi:10.2307/3793147.

Rognes, T., Flouri, T., Nichols, B., Quince, C., and Mahé, F. (2016). VSEARCH: a versatile open source tool for metagenomics. *PeerJ* 4, e2584. doi:10.7717/peerj.2584.

Ruijter, J. M., Ramakers, C., Hoogaars, W. M. H., Karlen, Y., Bakker, O., Hoff, V. D., et al. (2009). Amplification efficiency: linking baseline and bias in the analysis of quantitative PCR data. *Nucleic Acids Res* 37, e45–e45. doi:10.1093/nar/gkp045.

Rukachaisirikul, V., Buadam, S., Sukpondma, Y., Phongpaichit, S., Sakayaroj, J., and Hutadilok-Towatana, N. (2013). Indanone and mellein derivatives from the Garcinia-derived fungus Xylaria sp. PSU-G12. *Phytochemistry Letters* 6, 135–138. doi:10.1016/j.phytol.2012.11.007.

Scholtysik, A., Unterseher, M., Otto, P., and Wirth, C. (2012). Spatio-temporal dynamics of endophyte diversity in the canopy of European ash (*Fraxinus excelsior*). *Mycological Progress*, 1–14.

Soca-Chafre, G., Rivera-Orduña, F. N., Hidalgo-Lara, M. E., Hernandez-Rodriguez, C., Marsch, R., and Flores-Cotera, L. B. (2011). Molecular phylogeny and paclitaxel screening of fungal endophytes from Taxus globosa. *Fungal Biology* 115, 143–156. doi:10.1016/j.funbio.2010.11.004.

Stamatakis, A., Hoover, P., Rougemont, J., and Renner, S. (2008). A Rapid Bootstrap Algorithm for the RAxML Web Servers. *Syst Biol* 57, 758–771. doi:10.1080/10635150802429642.

Taylor, D. L., Hollingsworth, T. N., McFarland, J. W., Lennon, N. J., Nusbaum, C., and Ruess, R. W. (2013). A first comprehensive census of fungi in soil reveals both hyperdiversity and fine-scale niche partitioning. *Ecological Monographs* 84, 3–20. doi:10.1890/12-1693.1.

Taylor, D. L., Walters, W. A., Lennon, N. J., Bochicchio, J., Krohn, A., Caporaso, J. G., et al. (2016). Accurate Estimation of Fungal Diversity and Abundance through Improved Lineage-Specific Primers Optimized for Illumina Amplicon Sequencing. *Appl. Environ. Microbiol.* 82, 7217–7226. doi:10.1128/AEM.02576-16.

Tedersoo, L., Bahram, M., Puusepp, R., Nilsson, R. H., and James, T. Y. (2017). Novel soil-inhabiting clades fill gaps in the fungal tree of life. *Microbiome* 5, 42. doi:10.1186/s40168-017-0259-5.

Tedersoo, L., and Lindahl, B. (2016). Fungal identification biases in microbiome projects. *Environmental Microbiology Reports* 8, 774–779. doi:10.1111/1758-2229.12438.

Timling, I., Walker, D. A., Nusbaum, C., Lennon, N. J., and Taylor, D. L. (2014). Rich and cold: diversity, distribution and drivers of fungal communities in patterned-ground ecosystems of the North

American Arctic. *Mol. Ecol.* 23, 3258–3272. doi:10.1111/mec.12743.

Toju, H., Tanabe, A. S., Yamamoto, S., and Sato, H. (2012). High-Coverage ITS Primers for the DNA-Based Identification of Ascomycetes and Basidiomycetes in Environmental Samples. *PLoS ONE* 7, e40863. doi:10.1371/journal.pone.0040863.

Tondello, A., Vendramin, E., Villani, M., Baldan, B., and Squartini, A. (2012). Fungi associated with the southern Eurasian orchid Spiranthes spiralis (L.) Chevall. *Fungal Biology* 116, 543–549. doi:10.1016/j.funbio.2012.02.004.

U'Ren, J. M., Lutzoni, F., Miadlikowska, J., Laetsch, A. D., and Arnold, A. E. (2012). Host and geographic structure of endophytic and endolichenic fungi at a continental scale. *Am. J. Bot.* 99, 898–914. doi:10.3732/ajb.1100459.

U'Ren, J. M., Miadlikowska, J., Zimmerman, N. B., Lutzoni, F., Stajich, J. E., and Arnold, A. E. (2016). Contributions of North American endophytes to the phylogeny, ecology, and taxonomy of Xylariaceae (Sordariomycetes, Ascomycota). *Molecular Phylogenetics and Evolution* 98, 210–232. doi:10.1016/j.ympev.2016.02.010.

VanKuren Nicholas W., den Bakker Henk C., Morton Joseph B., and Pawlowska Teresa E. (2013). Ribosomal rna gene diversity, effective population size, and evolutionary longevity in asexual glomeromycota. *Evolution* 67, 207–224. doi:10.1111/j.1558-5646.2012.01747.x.

Wu, J.-H., Hong, P.-Y., and Liu, W.-T. (2009). Quantitative effects of position and type of single mismatch on single base primer extension. *Journal of Microbiological Methods* 77, 267–275. doi:10.1016/j.mimet.2009.03.001.

Yu, G., Smith, D., Zhu, H., Guan, Y., and Lam, T. T.-Y. (2017). ggtree: an R package for visualization and annotation of phylogenetic trees with their covariates and other associated data. *Methods in Ecology and Evolution* 8, 28–36. doi:10.1111/2041-210X.12628.

Zhang, J., and Li, K. (2003). Single-base discrimination mediated by proofreading 3′ phosphorothioate-modified primers. *Molecular biotechnology* 25, 223–227.
