# Supplementary material for: The Endophytic Mycobiome of European Ash and Sycamore Maple Leaves – Geographic Patterns, Host Specificity and Influence of Ash Dieback
Source: Front Microbiol. 2018 Oct 24;9:2345. doi: 10.3389/fmicb.2018.02345 (PMC6207852; doi:10.3389/fmicb.2018.02345)
Supplement: Supplementary file 7 [file Data_Sheet_2.pdf]

Supplementary Data Sheet 2: List of cultured isolates

This following pages contain a list of isolate clones (isolates with unique ITS sequences) and their taxonomic assignment. Some isolate clones were already published by Ibrahim et al. (2017), but were again included here because most were also isolated from *F. excelsior* and/or *A. pseudoplatanus*. Their taxonomic assignments were partly different, but GenBank records have been changed to be in line with this list. *Venturia* spp. sequences were all published by Ibrahim et al. (2016) and are therefore not included here.

Explanatation of table columns:

Isolate code: Morphotype code followed by an underscore and the clone (isolate with unique sequence) number. These codes are used in the publication to identify isolate clones. Morphotypes

Host species: Shows, how many of the sequenced isolates originated from which host species. Fe = *F. excelsior*, Fo = *F. ornus*, Ap = *A. pseudoplatanus*.

Genbank accession(s): GenBank accessions for sequences uploaded for this study or from Ibrahim et al., (2017), consisting of the whole ITS region.

BLAST: reference seq. (best hit): The accession of a sequence that was used as reference for identification. The sequence appeared in a phylogenetic publication (see **reference** column). Acessions written in brackets denote the top hit from the BLAST search instead.

Code (classification type):

T	Type: sequence from type record of species (NCBI RefSeq, NR_xxxxxx numbers)
G	Good: reliable hit (near 100%) found (published in a taxonomic publication)
B	Bad resolution: multiple reliable hits at high identities or identical, therefore not clear which species, ITS not enough to distinguish
M	Many hits from the same species above 97%, but none from reliable source found. Still regarded as safe to assign this species.
S	Many different species above 97% identities, no reliable source found. Therefore, only assigning the genus.
N	No clear hit: possibly unreliable species assignments, genus name or higher rank used.
Nr	No good reference: unclear how hits were identified.
A	Sequence has ambiguities due to poor quality, identification and identities not certain

***Hf* germ. rate:** Mean germination rate of *H. fraxineus* ascospores on agar plucks containing exudates from one or more cultures of the given clone, as published by Schlegel et al. (2016). Low percentages indicate that the metabolites produced by this endophyte inhibited spore germination of the pathogen. Additionally, the isolate names used in the mentioned publication are given in brackets because they differ from the codes used here. They can be found in the supplementary file '*Table S1 Isolates.xlsx'* of Schlegel et al. (2016). Also note, that some taxonomic assignments differ because the taxonomy was re-evaluated for this publication.

Isolate code	class	order	family	species	Host species: Fe	Host species: Fo	Host species: Ap	Genbank accession(s)	BLAST: reference seq. (best hit): accession	BLAST: reference seq. (best hit): ident.	code	*Hf* germ. rate	reference
Gug_1	Dothideomycetes	Botryosphaeriales	Phyllostictaceae	Guignardia gaultheriae	0	1	0	MH935065	KF766169 (NR_144916)	100.0%	T		(Slippers et al., 2013; Su and Cai, 2012)
Bd_1	Dothideomycetes	Botryosphaeriales	Botryosphaeriaceae	Botryosphaeria dothidea	2	4	1	KY367495 MH935081	(KY367495)	100.0%	M	84.6% (Bd_1/2)	
Bd_2	Dothideomycetes	Botryosphaeriales	Botryosphaeriaceae	Botryosphaeria dothidea	0	1	0	MH935068	(KY367495)	100.0%	M		
Jasp_1	Sordariomycetes	Calosphaeriales	Calosphaeriaceae	Jattaea sp.	0	2	0	MH934980	(KT823783)	100.0%	S	71.7% (Pc_1)	
Clsp_1	Dothideomycetes	Capnodiales	Cladosporiaceae	Cladosporium sp.	0	1	0	MH935067	(KY646223)	99.1%	?		
Myc_1	Dothideomycetes	Capnodiales	Mycosphaerellaceae	Mycosphaerella sp.	0	1	0	KY367534	KP894230	99.3%	S		(Videira et al., 2015)
Myc_2	Dothideomycetes	Capnodiales	Mycosphaerellaceae	Mycosphaerella sp.	0	1	0	MH935001	KP894321	100.0%	S		(Videira et al., 2015)
Myc_3	Dothideomycetes	Capnodiales	Mycosphaerellaceae	Ramularia unterseheri	0	2	0	MH935002	KP894274	100.0%	G		(Videira et al., 2015)
Myc_4	Dothideomycetes	Capnodiales	Mycosphaerellaceae	Ramularia vizellae	0	1	0	KY367521	KP894316	100.0%	G	98.1% (My1_1)	(Videira et al., 2015)
Mysp_1	Dothideomycetes	Capnodiales	Mycosphaerellaceae	Mycosphaerella sp.	1	5	0	KY367493	KF901516	100.0%	G?	95.7% (My2_1)	(Quaedvlieg et al., 2014)
Mysp_2	Dothideomycetes	Capnodiales	Mycosphaerellaceae	Mycosphaerella sp.	0	1	0	KY367492	(KY367492)	100.0%	N		(Quaedvlieg et al., 2014)
Mysp_3	Dothideomycetes	Capnodiales	Mycosphaerellaceae	Mycosphaerella sp.	1	0	0	MH935032	(KX058163)	98.9%	N	95.7% (My2_2)	
UnP_1	Dothideomycetes	Pleosporales		Unidentified Pleosporales UnP_1	1	0	0	MH935046	(KX096684)	99.6%	N		
Mysp2_1	Dothideomycetes	Capnodiales	Mycosphaerellaceae	Mycosphaerella sp.	0	5	0	KY367511 MH935078	(KY367511)	100.0%	N	72.7% (Ra_1/2)	
Mysp_4	Dothideomycetes	Capnodiales	Mycosphaerellaceae	Mycosphaerella sp.	0	4	0	KY367494 MH935080	(KY367494)	100.0%	B	92.2% (My3_1/2)	(Quaedvlieg et al., 2013)
Ral_1	Dothideomycetes	Capnodiales	Mycosphaerellaceae	Ramularia lethalis	0	0	1	MH935049	KX287472	100.0%	G		(Videira et al., 2016)
Ral_2	Dothideomycetes	Capnodiales	Mycosphaerellaceae	Ramularia lethalis	0	0	1	MH935047	KX287472	99.8%	G		(Videira et al., 2016)
My3_1	Dothideomycetes	Capnodiales	Mycosphaerellaceae	Sphaerulina amelanchier	0	1	0	MH934981	KF251597	100.0%	G		(Quaedvlieg et al., 2013; Verkley et al., 2013)
My3_3	Dothideomycetes	Capnodiales	Mycosphaerellaceae	Sphaerulina amelanchier	2	2	0	MH934982	KF251596	100.0%	G		(Quaedvlieg et al., 2013; Verkley et al., 2013)
My3_5	Dothideomycetes	Capnodiales	Mycosphaerellaceae	Sphaerulina amelanchier	0	1	0	KY367503	KF251596	100.0%	G		(Quaedvlieg et al., 2013; Verkley et al., 2013)
My3_6	Dothideomycetes	Capnodiales	Mycosphaerellaceae	Sphaerulina sp.	0	1	0	KY367522	(KM407488)	99.6%	S		
Spa_1	Dothideomycetes	Capnodiales	Mycosphaerellaceae	Sphaerulina aceris	0	0	2	MH935024	KF251593	100.0%	G		(Verkley et al., 2013)
Di1_1	Sordariomycetes	Diaporthales	Diaporthaceae	Diaporthe eres	1	1	2	KY367489	NR_144923	100.0%	G	93.3% (Df_1)	(Udayanga et al., 2014)
Disp_1	Sordariomycetes	Diaporthales	Diaporthaceae	Diaporthe sp.	0	1	0	KY367510	KC343205	99.4%	G		(Gomes et al., 2013)

Di2_1	Sordariomycetes	Diaporthales	Diaporthaceae	Diaporthe oncostoma	0	2	0	KY367500	KC343160	100.0%	G	85.2% (Di1_1)	(Gomes et al., 2013)
Di1_2	Sordariomycetes	Diaporthales	Diaporthaceae	Diaporthe eres	0	1	0	MH935025	KJ210518	100.0%	G		(Udayanga et al., 2014)
Di1_3	Sordariomycetes	Diaporthales	Diaporthaceae	Diaporthe eres	1	1	0	MH934983	KJ210516	100.0%	G	93.6% (Di2_1/2)	(Udayanga et al., 2014)
Di1_6	Sordariomycetes	Diaporthales	Diaporthaceae	Diaporthe eres	0	1	0	MH934984	KJ210530 / KC343150	100.0%	G	89.4% (Di3_1)	(Udayanga et al., 2014)
Di1_7	Sordariomycetes	Diaporthales	Diaporthaceae	Diaporthe rudis	1	3	0	KY367490	KC343058	100.0%	G		(Gomes et al., 2013)
Di1_8	Sordariomycetes	Diaporthales	Diaporthaceae	Diaporthe rudis	0	1	0	MH934985	KC343230	100.0%	G		(Gomes et al., 2013)
Ape_1	Sordariomycetes	Diaporthales	Gnomoniaceae	Apiognomonia errabunda	0	3	1	KY367512	DQ313524	100.0%	G		(Sogonov et al., 2007)
Ape_2	Sordariomycetes	Diaporthales	Gnomoniaceae	Apiognomonia errabunda	1	1	0	MH934986	DQ313526	100.0%	G		(Sogonov et al., 2007)
Dq_1	Sordariomycetes	Diaporthales	Gnomoniaceae	Discula sp.	0	3	0	KY367498	NR_137003	99.8%	G	88.0% (Dq_1)	(Sogonov et al., 2008)
Ac_1	Sordariomycetes	Diaporthales	Gnomoniaceae	Amphiporthe castanea	0	4	0	KY367502	KM082989 / KM082990	100.0%	G		(T. N. Sieber, unpublished)
Ops_1	Sordariomycetes	Diaporthales	Gnomoniaceae	Ophiognomonia setacea	0	3	0	MH934987	AY818953	100.0%	G		(Sogonov et al., 2005)
Opl_1	Sordariomycetes	Diaporthales	Gnomoniaceae	Ophioceras leptosporum	0	0	1	MH935035	NR_111768	99.8%	T		(Luo and Zhang, 2013)
Pli_1	Sordariomycetes	Diaporthales	Gnomoniaceae	Plagiostoma inclinatum	0	0	1	MH935041	EU255034	99.8%	G		(Sogonov et al., 2008)
Gno_1	Sordariomycetes	Diaporthales	Gnomoniaceae	Plagiostoma sp.	1	0	0	MH935055	(GU367037)	99.4%	S		(Mejía et al., 2011)
Gno_2	Sordariomycetes	Diaporthales	Gnomoniaceae	Apiognomonia hystrix	1	0	0	MH935045	EU255030	100.0%	G		(Mejía et al., 2011; Sogo-nov et al., 2008)
Aup_1	Dothideomycetes	Dothideales	Dothioraceae	Aureobasidium pullulans	0	1	1	KY367526	(MF055667)	100.0%	M	95.3% (Ap_1)	
Aus_1	Dothideomycetes	Dothideales	Dothioraceae	Aureobasidium subglaciale	1	0	0	MH935037	FJ150896	100.0%	G	95.0% (Ap2_1)	(Zalar et al., 2008)
Ca1_1	Sordariomycetes	Glomerellales	Glomerellaceae	Colletotrichum acutatum	0	1	2	MH934988	(KX529647)	100.0%	?		
Ca1_2	Sordariomycetes	Glomerellales	Glomerellaceae	Colletotrichum acutatum	2	3	1	KY367488 MH935072	(KY367488)	100.0%	?	89.0% (Ca1_1/2)	
Cog_1	Sordariomycetes	Glomerellales	Glomerellaceae	Colletotrichum godetiae	0	3	0	MH934989	NR_103691	100.0%	G		(Damm et al., 2012)
Cog_2	Sordariomycetes	Glomerellales	Glomerellaceae	Colletotrichum godetiae	1	0	0	MH934990	(KY171892)	99.8%	G		
Cosp2_1	Sordariomycetes	Glomerellales	Glomerellaceae	Colletotrichum sp.	1	1	0	MH934991	NR_144797	100.0%	S		(Doyle et al., 2013)
Plc_1	Sordariomycetes	Glomerellales	Plectosphaerellaceae	Plectosphaerella cucumerina	0	0	1	MH935031	KY399816	100.0%	G		(Su et al., 2017)
Na_1	Leotiomycetes	Helotiales	Dermateaceae	Phlyctema vagabunda	0	2	0	KY367506	KY173432	100.0%	N		(Crous et al., 2016)
Na_2	Leotiomycetes	Helotiales	Dermateaceae	Phlyctema vagabunda	1	5	0	KY367507 MH935076	(KY367507)	100.0%	M	47.8% (Na_1/2)	
Hesp2_1	Leotiomycetes	Helotiales		Helotiales sp.	0	0	1	MH935052	(FJ820724)	98.4%	N		

Hesp2_2	Leotiomycetes	Helotiales		Helotiales sp.	1	0	0	MH934992	(KT699138)	99.8%	N		
Hesp2_3	Leotiomycetes	Helotiales		Helotiales sp.	0	0	1	MH934993	(KT699138)	97.3%	N		
Hesp2_4	Leotiomycetes	Helotiales		Helotiales sp.	0	0	1	MH934994	(FJ820724)	99.4%	N		
Hesp2_5	Leotiomycetes	Helotiales		Helotiales sp.	0	0	1	MH934995	(FJ820724)	100.0%	N		
Hesp2_6	Leotiomycetes	Helotiales		Helotiales sp.	0	0	1	MH934996	(FJ820724)	99.8%	N		
Hesp2_7	Leotiomycetes	Helotiales		Helotiales sp.	0	0	2	MH935026	(KT699138)	96.7%	N		
Hym_1	Leotiomycetes	Helotiales	Helotiaceae	Hymenoscyphus fraxineus	2	0	0	MH934997	GU586904	100.0%	G		
Daf_1	Leotiomycetes	Helotiales	Hyaloscyphaceae	Dasyscyphus fuscescens	0	2	0	MH934998	(LT604877)	99.5%	N		
Clr_1	Sordariomycetes	Hypocreales	Bionectriaceae	Clonostachys rosea	0	0	1	MH935056	(KJ207401)	98.1%	A		
Coa_1	Sordariomycetes	Hypocreales	Nectriaceae	Cosmospora arxii	1	0	0	MH935039	(NR_145062)	100.0%	T	95.4% (Co_1)	(Lombard et al., 2015)
Fut_1	Sordariomycetes	Hypocreales	Nectriaceae	Fusarium tricinctum	0	0	1	MH935030	(KX058065)	100.0%	Nr		
Fut_2	Sordariomycetes	Hypocreales	Nectriaceae	Fusarium tricinctum	0	1	0	MH935071	(MF509746)	100.0%	Nr		
Fut_3	Sordariomycetes	Hypocreales	Nectriaceae	Fusarium tricinctum	0	0	1	MH934999	(MF509747)	100.0%	Nr		
Fusp_1	Sordariomycetes	Hypocreales	Nectriaceae	Fusarium sp.	1	3	0	KY367523	(LT746246)	100.0%	Nr	95.0% (Fu1_1)	
Fusp_2	Sordariomycetes	Hypocreales	Nectriaceae	Fusarium sp.	1	0	1	MH935000	(KT948363)	100.0%	Nr	0.0% (Fu2_1)	
Ned_1	Sordariomycetes	Hypocreales	Nectriaceae	Nectria dematiosa	0	0	1	MH935044	HM484557	100.0%	G		(Gräfenhan et al., 2011)
Stsp_1	Sordariomycetes	Hypocreales	Nectriaceae	Stylonectria sp.	0	0	1	MH935043	(JN053146)	99.0%	N		(Fan et al., 2017)
Elsp_1	Dothideomycetes	Myriangiales	Elsinoaceae	Elsinoe sp.	0	2	0	KY367513	NR_148168	98.7%	N	0.0% (El_1/2)	(Fan et al., 2017)
Elsp_2	Dothideomycetes	Myriangiales	Elsinoaceae	Elsinoe sp.	0	1	0	MH935058	NR_148168	98.7%	N		
Pesp_1	Pezizomycetes			Pezizomycetes sp.	0	0	1	MH935053	(KJ754181)	98.8%	N		
Pem_1	Dothideomycetes	Pleosporales	Periconiaceae	Periconia macrospinosa	0	2	0	KY367532	(KY367532)	99.8%	Nr		(Aveskamp et al., 2010)
Be_1	Dothideomycetes	Pleosporales	Didymellaceae	Boeremia sp.	1	3	0	KY367496 MH935075	NR_135985	100.0%	B	0.0% (Be_1)	(Aveskamp et al., 2010)
Be_3	Dothideomycetes	Pleosporales	Didymellaceae	Boeremia sp.	0	1	0	MH935070	NR_135985	100.0%	B		(Woudenberg et al., 2009)
Div_1	Dothideomycetes	Pleosporales	Didymellaceae	Didymella vitalbina	1	1	0	KY367524	FJ515592	100.0%	G		(Chen et al., 2017)
Epsp_1	Dothideomycetes	Pleosporales	Didymellaceae	Epicoccum sp.	0	0	1	MH935048	KY742114 / KY742112 / KY742108	100.0%	B		(Aveskamp et al., 2010)
Disp2_1	Dothideomycetes	Pleosporales	Didymellaceae	Didymella sp.	0	0	1	MH935029	(FJ228170)	99.1%	N		(Aveskamp et al., 2010)
Div2_1	Dothideomycetes	Pleosporales	Didymellaceae	Didymella viburnicola	0	1	0	MH935004	GU237872	100.0%	G		(Aveskamp et al., 2010)
Phsp4_1	Dothideomycetes	Pleosporales	Didymellaceae	Phoma sp.	0	1	0	MH935059	(KT898791)	100.0%	Nr		(Aveskamp et al., 2010)
Phsp4_2	Dothideomycetes	Pleosporales	Didymellaceae	Phoma sp.	0	1	0	MH935066	(KU366282)	100.0%	Nr		(Aveskamp et al., 2010)
Asp_1	Dothideomycetes	Pleosporales	Didymellaceae	Ascochyta pisi	0	2	0	KY367519	NR_135981	100.0%	T		(Aveskamp et al., 2010)
Mio_1	Dothideomycetes	Pleosporales	Didymellaceae	Microsphaeropsis olivacea	0	1	0	MH935003	GU237803	100.0%	G		(Chen et al., 2017)

Dim_1	Dothideomycetes	Pleosporales	Didymellaceae	Didymella macrostoma	2	4	0	KY367515 MH935083	KY742072 / GU237740	100.0%	G	93.2% (Ph3_1/2)	
UnL_1	Dothideomycetes	Pleosporales	Leptosphaeriaceae	Unidentified Leptosphaeriaceae UnL_1	0	1	0	MH935063	(FR773224)	99.1%	N		(Verkley et al., 2014)
Aq_1	Dothideomycetes	Pleosporales	Leptosphaeriaceae	Ampelomyces quisqualis	0	3	0	KY367508	JX681067	100.0%	G	34.7% (Aq_1/2)	
Ple_1	Dothideomycetes	Pleosporales	Pleosporaceae	Pleospora sp.	0	2	0	KY367514	(KY367514)	100.0%	Nr		(Moral et al., 2016)
Ple_2	Dothideomycetes	Pleosporales	Didymellaceae	Phoma sp.	0	2	0	MH935005	KU973715	100.0%	?		(Crous and Groenewald, 2017)
Phsp2_1	Dothideomycetes	Pleosporales	Didymellaceae	Phoma sp.	0	1	0	KY367527	KY929148	99.8%	G?		(Crous and Groenewald, 2017)
Casp_1	Dothideomycetes	Pleosporales	Coniothyriaceae	Camarosporium sp.	1	0	0	MH935050	KY929138	99.8%	?		(Aveskamp et al., 2009; Quaedvlieg et al., 2013)
Phsp_1	Dothideomycetes	Pleosporales	Leptosphaeriaceae	Phaeosphaeria sp.	0	0	1	MH935057	KF251194	98.8%	N		(de Gruyter et al., 2013)
Pyr_1	Dothideomycetes	Pleosporales	Cucurbitariaceae	Pyrenochaeta cava	1	3	0	KY367517	JF740260	99.8%	G	2.0% (Pc_2)	
Pyr_2	Dothideomycetes	Pleosporales	Cucurbitariaceae	Pyrenochaeta sp.	0	1	0	MH935061	(FJ379833)	100.0%	?		(Verkley et al., 2014)
Ple2_1	Dothideomycetes	Pleosporales	Montagnulaceae	Paraconiothyrium brasiliense	0	0	2	MH935006	JX496032	99.8%	G		(Verkley et al., 2014)
Ple2_2	Dothideomycetes	Pleosporales		Unidentified Pleosporales Ple2_2	0	0	1	MH935033	(AB472197)	92.2%	N		(Verkley et al., 2014)
Ple2_3	Dothideomycetes	Pleosporales		Unidentified Pleosporales Ple2_3	0	0	1	MH935034	(AB472197)	92.2%	N		(Verkley et al., 2014)
Pa1_1	Dothideomycetes	Pleosporales	Montagnulaceae	Paraconiothyrium sp.	6	5	2	KY367491 MH935077 MH935074	(KY367491)	100.0%	N	0.5% (Pa1_1/2)	(Verkley et al., 2014)
Ple2_5	Dothideomycetes	Pleosporales	Montagnulaceae	Paraphaeosphaeria neglecta	0	1	0	MH935062	JX496107	100.0%	G	88.7% (Pa4_2)	(Verkley et al., 2014)
Nesp_1	Dothideomycetes	Pleosporales	Phaeosphaeriaceae	Neosetophoma sp.	1	0	0	MH935007	KF251162	98.4%	G?	96.4% (Ne1_1)	(Quaedvlieg et al., 2013)
Phn_1	Dothideomycetes	Pleosporales	Phaeosphaeriaceae	Phaeosphaeria nigrans	0	0	2	MH935008	KF251184 / AF439492	100.0%	G		(Quaedvlieg et al., 2013)
Pha2_1	Dothideomycetes	Pleosporales	Phaeosphaeriaceae	Muriphaeosphaeria viburni	1	0	0	MH935051	KX306761	100.0%	G?		(Hernandez-Restrepo et al., 2016)
Pha2_2	Dothideomycetes	Pleosporales	Phaeosphaeriaceae	Muriphaeosphaeria viburni	1	0	1	MH935027	KX306761	99.8%	G?		(Hernandez-Restrepo et al., 2016)
Pha2_3	Dothideomycetes	Pleosporales	Phaeosphaeriaceae	Unidentified Phaeosphaeriaceae Pha2_3	0	1	0	MH935064	(KC884303)	97.4%	N		
Ple2_6	Dothideomycetes	Pleosporales	Phaeosphaeriaceae	Phaeosphaeria sp.	1	0	0	MH935028	KF251182	93.1%	N		(Quaedvlieg et al., 2013)

Aa_1	Dothideomycetes	Pleosporales	Pleosporaceae	Alternaria alternata	1	4	1	KY367499 MH935073	(MF055661)	100.0%	M	75.5% (Aa_1)	
Aa_2	Dothideomycetes	Pleosporales	Pleosporaceae	Alternaria infectoria	0	2	0	KY367525	(KY496638)	100.0%	M		
Stv_1	Dothideomycetes	Pleosporales	Pleosporaceae	Stemphylium vesicarium	0	0	1	MH935054	KY883976	100.0%	G		(Woudenberg et al., 2017)
Prm_1	Dothideomycetes	Pleosporales	Sporormiaceae	Preussia minima	0	1	0	MH935010	GQ203786	100.0%	G		(Kruys and Wedin, 2009)
Nod_1	Dothideomycetes	Pleosporales	Phaeosphaeriaceae	Nodulosphaeria digitalis	2	0	0	MH935009	KU058710	100%? (am-bigs)	G		(Li et al., 2015)
UnA_1				Unidentified Ascomycota UnA_1	0	0	1	MH935042	(JQ070509)	99.3%	N		
Ann_1	Sordariomycetes	Xylariales	Xylariaceae	Annulohypoxylon cohaerens	0	1	0	MH935011	EF026140	100.0%	G?		(Hsieh et al., 2010)
Ann_2	Sordariomycetes	Xylariales	Xylariaceae	Annulohypoxylon michelianum	0	1	0	MH935069	KX376320	100.0%	G		(Kuhnert et al., 2017)
UnX_1	Sordariomycetes	Xylariales	Xylariaceae	Unidentified Xylariaceae UnX_1	0	2	0	MH935012	(GQ153206)	100.0%	N		
Bn_1	Sordariomycetes	Xylariales	Xylariaceae	Biscogniauxia nummularia	0	2	1	KY367501	AJ390415	100.0%	G		(Sánchez-Ballesteros et al., 2000)
Bn_3	Sordariomycetes	Xylariales	Xylariaceae	Biscogniauxia nummularia	0	2	1	MH935013	KY610382	100.0%	G	94.2% (Bn_1)	(Wendt et al., 2017)
Bn_4	Sordariomycetes	Xylariales	Xylariaceae	Biscogniauxia mediterranea	0	1	1	KY367529	KM216787	100.0%	G		(Henriques et al., 2016)
Crs2_1	Sordariomycetes	Xylariales	Xylariaceae	Creosphaeria sassafras	0	1	0	MH935060	KU683754	(92.2%)	A		(U'Ren et al., 2016)
Dac_1	Sordariomycetes	Xylariales	Xylariaceae	Daldinia childiae	1	2	0	KY367520	KY610413	100.0%	G?	93.0% (Da_1/2)	(Wendt et al., 2017)
Hyf2_1	Sordariomycetes	Xylariales	Xylariaceae	Hypoxylon fragiforme	1	0	0	MH935038	KU684022 / KU683752	100.0%	G		(U'Ren et al., 2016)
Hyr_1	Sordariomycetes	Xylariales	Xylariaceae	Hypoxylon rubiginosum	0	1	0	KY367528	KC968929	99.4%	G		(Kuhnert et al., 2014)
Hyr_3	Sordariomycetes	Xylariales	Xylariaceae	Hypoxylon rubiginosum	0	0	1	MH935014	KC968929	100.0%	G		(Kuhnert et al., 2014)
Kd_1	Sordariomycetes	Xylariales	Xylariaceae	Kretzschmaria deusta	0	2	1	KY367504	KU683767	100.0%	G	0.0% (Kd_1)	(U'Ren et al., 2016)
Xyl_1	Sordariomycetes	Xylariales	Xylariaceae	Nemania diffusa	1	5	3	MH935016 MH935082	KU683999	100.0%	G	14.1% (Xh_1/2)	(U'Ren et al., 2016)
Ns_1	Sordariomycetes	Xylariales	Xylariaceae	Nemania serpens	0	1	0	MH935017	KU683765	100.0%	G		(U'Ren et al., 2016)
Ns_2	Sordariomycetes	Xylariales	Xylariaceae	Nemania serpens	0	2	1	KY367497	(KU141386)	100.0%	M		(U'Ren et al., 2016)
Ns_3	Sordariomycetes	Xylariales	Xylariaceae	Nemania serpens	1	0	0	MH935018	(KU141386)	100.0%	M		(U'Ren et al., 2016)
Ns_5	Sordariomycetes	Xylariales	Xylariaceae	Unidentified Xylariaceae Ns_5	0	0	1	MH935015	KU684009	99.4%	N		(U'Ren et al., 2016)
Rc_1	Sordariomycetes	Xylariales	Xylariaceae	Rosellinia corticium	0	2	0	KY367509	KY610393	100.0%	G	94.9% (Rc_1)	(Wendt et al., 2017)

Rc_2	Sordariomycetes	Xylariales	Xylariaceae	Rosellinia corticium	1	0	0	MH935019	KY610393	100.0%	G	94.7% (Rc_2)	(Wendt et al., 2017)
Rc_3	Sordariomycetes	Xylariales	Xylariaceae	Rosellinia sp.	1	0	0	MH935020	(HQ823749)	100.0%	Nr	94.9% (Rd_1)	
Xyl_3	Sordariomycetes	Xylariales	Xylariaceae	Xylaria longipes	0	0	2	MH935023	KU683768	100.0%	G		(U'Ren et al., 2016)
Xyl_5	Sordariomycetes	Xylariales	Xylariaceae	Xylaria sp.	0	1	0	KY367535	(JQ761703)	100.0%	N		(U'Ren et al., 2016)
Rosp_1	Sordariomycetes	Xylariales	Xylariaceae	Rosellinia sp.	0	1	0	MH935021	(FJ820826)	100.0%	N		
Rosp_2	Sordariomycetes	Xylariales	Xylariaceae	Rosellinia sp.	1	0	0	MH935022	(FJ820826)	99.8%	N	94.6% (Xy1_1)	
Rosp_4	Sordariomycetes	Xylariales	Xylariaceae	Rosellinia sp.	0	2	0	KY367516 MH935079	(KY367516)	100.0%	N	77.4% (Xy1_2)	
Scc_1	Agaricomycetes	Agaricales	Schizophyllaceae	Schizophyllum commune	1	0	0	MH935036	(MF423716)	100.0%	M		
Mij_1	Exobasidiomy-cetes	Microstromatales	Microstromataceae	Pseudomicrostroma juglandis	0	2	0	KY367530	DQ317634 / DQ789988	100.0%	G		(de Beer et al., 2006)
Trh_1	Agaricomycetes	Polyporales	Coriolaceae	Trametes hirsuta	1	0	0	MH935040	HQ435859	99.8%	G		(Malysheva and Zmitrovich, 2011)

References

Aveskamp, M. M., de Gruyter, J., Woudenberg, J. H. C., Verkley, G. J. M., and Crous, P. W. (2010). Highlights of the Didymellaceae: A polyphasic approach to characterise *Phoma* and related pleosporalean genera. *Stud. Mycol.* 65, 1–60. doi:10.3114/sim.2010.65.01.

Aveskamp, M. M., Verkley, G. J. M., Gruyter, J. de, Murace, M. A., Perelló, A., Woudenberg, J. H. C., et al. (2009). DNA phylogeny reveals polyphyly of *Phoma* section *Peyronellaea* and multiple taxonomic novelties. *Mycologia* 101, 363–382. doi:10.3852/08-199.

Chen, Q., Hou, L. W., Duan, W. J., Crous, P. W., and Cai, L. (2017). Didymellaceae revisited. *Stud. Mycol.* 87, 105–159. doi:10.1016/j.simyco.2017.06.002.

Crous, P. W., and Groenewald, J. Z. (2017). The Genera of Fungi – G 4: *Camarosporium* and *Dothiora*. *IMA Fungus* 8, 131–152. doi:10.5598/imafungus.2017.08.01.10.

Crous, P. W., Wingfield, M. J., Burgess, T. I., Hardy, G. E. S. J., Crane, C., Barrett, S., et al. (2016). Fungal Planet description sheets: 469–557. *Persoonia Mol. Phylogeny Evol. Fungi* 37, 218–403. doi:10.3767/003158516X694499.

Damm, U., Cannon, P. F., Woudenberg, J. H. C., and Crous, P. W. (2012). The *Colletotrichum acutatum* species complex. *Stud. Mycol.* 73, 37–113. doi:10.3114/sim0010.

de Beer, Z. W., Begerow, D., Bauer, R., Pegg, G. S., Crous, P. W., and Wingfield, M. J. (2006). Phylogeny of the Quambalariaceae fam. nov., including important *Eucalyptus* pathogens in South Africa and Australia. *Stud. Mycol.* 55, 289–298. doi:10.3114/sim.55.1.289.

de Gruyter, J., Woudenberg, J. H. C., Aveskamp, M. M., Verkley, G. J. M., Groenewald, J. Z., and Crous, P. W. (2013). Redisposition of *Phoma*-like anamorphs in Pleosporales. *Stud. Mycol.* 75, 1–36. doi:10.3114/sim0004.

Doyle, V. P., Oudemans, P. V., Rehner, S. A., and Litt, A. (2013). Habitat and Host Indicate Lineage Identity in *Colletotrichum gloeosporioides* s.l. from Wild and Agricultural Landscapes in North America. *PLOS ONE* 8, e62394. doi:10.1371/journal.pone.0062394.

Fan, X. L., Barreto, R. W., Groenewald, J. Z., Bezerra, J. D. P., Pereira, O. L., Cheewangkoon, R., et al. (2017). Phylogeny and taxonomy of the scab and spot anthracnose fungus *Elsinoë* (Myriangiales, Dothideomycetes). *Stud. Mycol.* 87, 1–41. doi:10.1016/j.simyco.2017.02.001.

Gomes, R. R., Glienke, C., Videira, S. I. R., Lombard, L., Groenewald, J. Z., and Crous, P. W. (2013). *Diaporthe*: a genus of endophytic, saprobic and plant pathogenic fungi. *Persoonia - Mol. Phylogeny Evol. Fungi* 31, 1–41. doi:10.3767/003158513X666844.

Gräfenhan, T., Schroers, H.-J., Nirenberg, H. I., and Seifert, K. A. (2011). An overview of the taxonomy, phylogeny, and typification of nectriaceous fungi in *Cosmospora, Acremonium, Fusarium, Stilbella*, and *Volutella*. *Stud. Mycol.* 68, 79–113. doi:10.3114/sim.2011.68.04.

Henriques, J., Nóbrega, F., Sousa, E., and Lima, A. (2016). Analysis of the genetic diversity and phylogenetic relationships of *Biscogniauxia mediterranea* isolates associated with cork oak. *Phytoparasitica* 44, 19–34. doi:10.1007/s12600-015-0503-0.

Hernandez-Restrepo, M., Schumacher, R. K., Wingfield, M. J., Ahmad, I., Cai, L., Duong, T. A., et al. (2016). Fungal systematics and evolution : FUSE 2. Available at: http://repository.up.ac.za/handle/2263/60641.

Hsieh, H.-M., Lin, C.-R., Fang, M.-J., Rogers, J. D., Fournier, J., Lechat, C., et al. (2010). Phylogenetic status of *Xylaria* subgenus *Pseudoxylaria* among taxa of the subfamily Xylarioideae (Xylariaceae) and phylogeny of the taxa involved in the subfamily. *Mol. Phylogenet. Evol.* 54, 957–969. doi:10.1016/j.ympev.2009.12.015.

Ibrahim, M., Schlegel, M., and Sieber, T. N. (2016). *Venturia orni* sp. nov., a species distinct from *Venturia fraxini* living in the leaves of *Fraxinus ornus*. *Mycol Progress in press*.

Ibrahim, M., Sieber, T. N., and Schlegel, M. (2017). Communities of fungal endophytes in leaves of *Fraxinus ornus* are highly diverse. *Fungal Ecol.* 29, 10–19. doi:10.1016/j.funeco.2017.05.001.

Kruys, Å., and Wedin, M. (2009). Phylogenetic relationships and an assessment of traditionally used taxonomic characters in the Sporormiaceae (Pleosporales, Dothideomycetes, Ascomycota), utilising multi-gene phylogenies. *Syst. Biodivers.* 7, 465–478.

Kuhnert, E., Fournier, J., Peršoh, D., Luangsa-ard, J. J. D., and Stadler, M. (2014). New *Hypoxylon* species from Martinique and new evidence on the molecular phylogeny of *Hypoxylon* based on ITS rDNA and β-tubulin data. *Fungal Divers.* 64, 181–203. doi:10.1007/s13225-013-0264-3.

Kuhnert, E., Sir, E. B., Lambert, C., Hyde, K. D., Hladki, A. I., Romero, A. I., et al. (2017). Phylogenetic and chemotaxonomic resolution of the genus *Annulohypoxylon* (Xylariaceae) including four new species. *Fungal Divers.* 85, 1–43. doi:10.1007/s13225-016-0377-6.

Li, W. J., Bhat, D. J., Camporesi, E., Tian, Q., Wijayawardene, N. N., Dai, D. Q., et al. (2015). New asexual morph taxa in Phaeosphaeriaceae. *Mycosphere* 6, 681–708.

Lombard, L., van der Merwe, N. A., Groenewald, J. Z., and Crous, P. W. (2015). Generic concepts in Nectriaceae. *Stud. Mycol.* 80, 189–245. doi:10.1016/j.simyco.2014.12.002.

Luo, J., and Zhang, N. (2013). Magnaporthiopsis, a new genus in Magnaporthaceae (Ascomycota). *Mycologia* 105, 1019–1029. doi:10.3852/12-359.

Malysheva, V. F., and Zmitrovich, I. V. (2011). Testing the *Trametes hirsuta* complex. *Nova Hedwig.* 93, 57–71. doi:10.1127/0029-5035/2011/0093-0057.

Mejía, L. C., Castlebury, L. A., Rossman, A. Y., Sogonov, M. V., and White, J. F. (2011). A systematic account of the genus Plagiostoma (Gnomoniaceae, Diaporthales) based on morphology, host-associations, and a four-gene phylogeny. *Stud. Mycol.* 68, 211–235. doi:10.3114/sim.2011.68.10.

Moral, J., Agustí-Brisach, C., Pérez-Rodríguez, M., Xaviér, C., Raya, M. C., Rhouma, A., et al. (2016). Identification of Fungal Species Associated with Branch Dieback of Olive and Resistance of Table Cultivars to Neofusicoccum mediterraneum and Botryosphaeria dothidea. *Plant Dis.* 101, 306–316. doi:10.1094/PDIS-06-16-0806-RE.

Quaedvlieg, W., Binder, M., Groenewald, J. Z., Summerell, B. A., Carnegie, A. J., Burgess, T. I., et al. (2014). Introducing the Consolidated Species Concept to resolve species in the Teratosphaeriaceae. *Persoonia Mol. Phylogeny Evol. Fungi* 33, 1–40. doi:10.3767/003158514X681981.

Quaedvlieg, W., Verkley, G. J. M., Shin, H.-D., Barreto, R. W., Alfenas, A. C., Swart, W. J., et al. (2013). Sizing up *Septoria*. *Stud. Mycol.* 75, 307–390. doi:10.3114/sim0017.

Sánchez-Ballesteros, J., González, V., Salazar, O., Acero, J., Portal, M. A., Julián, M., et al. (2000). Phylogenetic study of *Hypoxylon* and related genera based on ribosomal ITS sequences. *Mycologia* 92, 964–977. doi:10.2307/3761591.

Schlegel, M., Dubach, V., Buol, L. von, and Sieber, T. N. (2016). Effects of endophytic fungi on the ash dieback pathogen. *FEMS Microbiol. Ecol.* 92, fiw142. doi:10.1093/femsec/fiw142.

Slippers, B., Boissin, E., Phillips, A. J. L., Groenewald, J. Z., Lombard, L., Wingfield, M. J., et al. (2013). Phylogenetic lineages in the Botryosphaeriales: a systematic and evolutionary framework. *Stud. Mycol.* 76, 31–49. doi:10.3114/sim0020.

Sogonov, M. V., Castlebury, L. A., Rossman, A. Y., Farr, D. F., and White, J. F. (2005). The type species of the genus *Gnomonia, G. gnomon*, and the closely related *G. setacea*. *SYDOWIA-HORN-* 57, 102.

Sogonov, M. V., Castlebury, L. A., Rossman, A. Y., Mejía, L. C., and White, J. F. (2008). Leaf-inhabiting genera of the Gnomoniaceae, Diaporthales. *Stud. Mycol.* 62, 1–77. doi:10.3114/sim.2008.62.01.

Sogonov, M. V., Castlebury, L. A., Rossman, A. Y., and White, J. F. (2007). The type species of *Apiognomonia, A. veneta* with its *Discula* anamorph is distinct from *A. errabunda*. *Mycol. Res.* 111, 693–709. doi:10.1016/j.mycres.2007.03.013.

Su, L., Deng, H., and Niu, Y.-C. (2017). Phylogenetic analysis of *Plectosphaerella* species based on multi-locus DNA sequences and description of *P. sinensis* sp. nov. *Mycol. Prog.* 16, 823–829. doi:10.1007/s11557-017-1319-8.

Su, Y. Y., and Cai, L. (2012). Polyphasic characterisation of three new *Phyllosticta* spp. *Persoonia Mol. Phylogeny Evol. Fungi* 28, 76–84. doi:10.3767/003158512X645334.

Udayanga, D., Castlebury, L. A., Rossman, A. Y., Chukeatirote, E., and Hyde, K. D. (2014). Insights into the genus *Diaporthe*: phylogenetic species delimitation in the *D. eres* species complex. *Fungal Divers.* 67, 203–229. doi:10.1007/s13225-014-0297-2.

U'Ren, J. M., Miadlikowska, J., Zimmerman, N. B., Lutzoni, F., Stajich, J. E., and Arnold, A. E. (2016). Contributions of North American endophytes to the phylogeny, ecology, and taxonomy of Xylariaceae (Sordariomycetes, Ascomycota). *Mol. Phylogenet. Evol.* 98, 210–232. doi:10.1016/j.ympev.2016.02.010.

Verkley, G. J. M., Dukik, K., Renfurm, R., Göker, M., and Stielow, J. B. (2014). Novel genera and species of coniothyrium-like fungi in Montagnulaceae (Ascomycota). *Persoonia Mol. Phylogeny Evol. Fungi* 32, 25–51. doi:10.3767/003158514X679191.

Verkley, G. J. M., Quaedvlieg, W., Shin, H.-D., and Crous, P. W. (2013). A new approach to species delimitation in *Septoria*. *Stud. Mycol.* 75, 213–305. doi:10.3114/sim0018.

Videira, S. I. R., Groenewald, J. Z., Braun, U., Shin, H. D., and Crous, P. W. (2016). All that glitters is not *Ramularia*. *Stud. Mycol.* 83, 49–163. doi:10.1016/j.simyco.2016.06.001.

Videira, S. I. R., Groenewald, J. Z., Verkley, G. J. M., Braun, U., and Crous, P. W. (2015). The rise of Ramularia from the Mycosphaerella labyrinth. *Fungal Biol.* 119, 823–843. doi:10.1016/j.funbio.2015.06.003.

Wendt, L., Sir, E. B., Kuhnert, E., Heitkämper, S., Lambert, C., Hladki, A. I., et al. (2017). Resurrection and emendation of the Hypoxylaceae, recognised from a multigene phylogeny of the Xylariales. *Mycol. Prog.*, 1–40. doi:10.1007/s11557-017-1311-3.

Woudenberg, J. H. C., Aveskamp, M. M., de Gruyter, J., Spiers, A. G., and Crous, P. W. (2009). Multiple *Didymella* teleomorphs are linked to the *Phoma clematidina* morphotype. *Persoonia* 22, 56–62. doi:10.3767/003158509X427808.

Woudenberg, J. H. C., Hanse, B., van Leeuwen, G. C. M., Groenewald, J. Z., and Crous, P. W. (2017). *Stemphylium* revisited. *Stud. Mycol.* 87, 77–103. doi:10.1016/j.simyco.2017.06.001.

Zalar, P., Gostinčar, C., de Hoog, G. S., Uršič, V., Sudhadham, M., and Gunde-Cimerman, N. (2008). Redefinition of *Aureobasidium pullulans* and its varieties. *Stud. Mycol.* 61, 21–38. doi:10.3114/sim.2008.61.02.
