# Supplementary material for: The Endophytic Mycobiome of European Ash and Sycamore Maple Leaves – Geographic Patterns, Host Specificity and Influence of Ash Dieback
Source: Front Microbiol. 2018 Oct 24;9:2345. doi: 10.3389/fmicb.2018.02345 (PMC6207852; doi:10.3389/fmicb.2018.02345)
Supplement: Supplementary file 8 [file Data_Sheet_3.PDF]

Supplementary Data Sheet 3: Comparison of identified cultures and NGS read abundances

The summary is done for morphotypes, and in addition, all OTUs with > 20,000 reads in total were included (Otu…).
Combinations with zero cultures / reads are shown as blank cells to improve readability.

Explanation of column labels

**P**	Fungal phylum (A=Ascomycota, B=Basidiomycota)
**cultured**	Mean number of cultures expected in 32 leaf discs/petiole pieces, corresponding to the total number of fungi found on 16 trees.
**NGS**	Mean number of NGS reads per sample (scaled to a sample total of 25,000)
**L/P**	leaf part: laminae / petioles
**OTUs**	OTU numbers matching the ITS sequence of the given morphotype, sorted by decreasing abundance in the NGS dataset (with % deviation from morphotype ITS sequence, if not 100% identical).

Colour codes

red	This morphotype was very rare in the NGS dataset (< 5 reads), but isolated with a mean frequency of > 0.1 per 16 trees.
purple	Found by culture in other *Fraxinus* samplings (not shown), but not this study
blue	OTU only found by NGS sequencing not by culture

				Fraxinus excelsior				*Fraxinus ornus*				*Acer pseudoplatanus*				* data from Ibrahim et al. (2016)
				cultured		NGS		cultured*		NGS		cultured		NGS		
P	Order	Species	morpho-type	L	P	L	P	L	P	L	P	L	P	L	P	OTUs sorted by abundance (with % deviation from isolate ITS)
		(no ITS sequence)	(?)	2.74	2.56	0.0	0.0	1.31	0.78	0.0	0.0	4.77	3.74	0.0	0.0	
A	Venturiales	Venturia spp.	Ven	7.43	6.19	12204	9334	8.88	13.74	5086	11873	0.90	0.77	2926	10842	3, 19, 7, 2, 1, 5, 11 (2.5%), ...
A	Erysiphales	Phyllactinia_fraxini	Otu6			1971	108			14198	1090			23	7	6
A	Erysiphales	Sawadaea bicornis	Otu4			112	101			1	2			9105	425	4
A	Capnodiales	Cladosporium sp.	Clsp	0.62	0.71	102	450	0.08	0.03	2	435	0.26	0.65	38	436	25 (0.4%), 33 (0.8%)
A	Capnodiales	Mycosphaerella spp.	Mysp	1.94	1.06	241	212	3.94	1.62	43	349	2.32		4	2	39, 36, 99, 171 (0.4%), ...
A	Capnodiales	Ramularia lethalis	Ral			160	14	0.14	0.02	0.5	0.4	0.13		1728	0.3	13
A	Capnodiales	Sphaerulina aceris	Spa			2	1			0.8	0.8	0.13	0.39	3901	0.2	8, 22 (2.8%)
A	Capnodiales	Sphaerulina spp.	My3	0.44		352	79	0.18	0.05	39	28	0.77		0.5	72	24, 5065 (2.4%), 4693 (2.8%), ...
A	Capnodiales	Mycosphaerellaceae clone	Otu16			814	0.5			20	0.0			0.5	0.1	16
A	Capnodiales	Ramularia spp.	Myc			741	95	0.08	0.03	2	0.4			33	5	26, 37, 109 (0.4%), 763 (2.4%), ...
A	Capnodiales	Ramularia sp.	Mysp2			136	23	0.15		7	20			2	1	45, 283 (2%), 1594 (2%), ...
A	Diaporthales	Diaporthe spp.	Di1	6.28	10.52	125	866	1.28	1.17	17	255	1.94	3.87	15	188	27, 75, 64, 38, 225 (0.4%), ...
A	Diaporthales	Diaporthe oncostoma	Di2			1	0.1	0.14	0.13	6	20			0.0	0.0	378, 3191 (3%)
A	Diaporthales	Diaporthe sp.	Disp			3	66	0.08	0.08	0.0	0.0			0.8	0.0	138
A	Diaporthales	Diaporthe pustulata	Dip			0.0	3			0.0	0.0			0.3	0.0	687
A	Diaporthales	Apiognomonia errabunda	Ape	0.44	0.88	20	27	0.07	0.12	0.0	114	0.77	0.77	192	0.0	72 (2.6%), 95
A	Diaporthales	Plagiostoma sp. / Apiognomonia hystrix	Gno	0.18		32	182			0.3	10			188	546	30 (1.1%), 104, 199 (0.4%), ...
A	Diaporthales	Plagiostoma inclinatum	Pli			0.8	0.1			0.0	0.0	0.65		330	5	87 (0.4%), 153 (0.7%), ...
A	Diaporthales	Discula quercina	Dq			0.0	0.0	0.28	0.09	0.3	10			0.0	0.0	848, 1956 (3%)
A	Diaporthales	Amphiporthe castanea	Ac			1	1	0.19	0.07	6	8			0.2	0.0	437
A	Diaporthales	Ophiognomonia setacea	Ops			0.4	3	0.08	0.02	0.7	31			2	0.0	457, 510 (0.7%), 1403 (1.1%)
A	Diaporthales	Ophioceras leptosporum	Opl			0.0	12			0.0	0.0		0.13	0.0	0.0	386
A	Glomerellales	Colletotrichum acutatum	Ca1	0.44	0.88	119	4	1.51	0.73	182	525	2.84	3.48	16	977	18
A	Glomerellales	Colletotrichum godetiae	Cog		0.09	64	2	0.07		21	7			3	101	43
A	Glomerellales	Colletotrichum sp.	Cosp2	0.09		0.6	0.0	0.02		0.0	0.0			0.5	0.0	1598
A	Glomerellales	Colletotrichum sp.	Cosp			1	0.0			1	0.0			0.1	0.0	1908 (0.8%), 2077 (0.8%)
A	Glomerellales	Plectosphaerella cucumerina	Plc			0.1	2			0.0	0.0	0.13		0.0	0.0	564 (2.9%), 4299, 2220 (2.9%)
A	Botryosphaeriales	Botryosphaeria	Bd	0.80	0.71	19	31	0.73	0.26	1	0.1	0.39	1.68	0.0	0.0	110

		dothidea														
A	Botryosphaeriales	Guignardia gaultheriae	Gug			0.0	0.0		0.09	0.0	0.0			0.1	0.0	6591 (1.5%)
A	Helotiales	Helotiales sp.	Hesp2	0.18	0.27	8	97			2	4	2.32	3.74	10	854	56, 70 (0.4%), 140 (1.2%), ...
A	Helotiales	Helotiales sp.	Hesp			0.1	6			0.1	0.0			0.0	222	113 (0.4%)
A	Helotiales	Hymenoscyphus fraxineus / albidus	Hym	0.97	0.53	888	506			0.2	0.4			0.5	0.4	9, H_albidus
A	Helotiales	Phlyctema vagabunda	Na	0.35	0.27	3	33	0.27	0.20	0.0	433			0.2	59	84
A	Helotiales	Helotiales clone	Otu80			1	19			0.0	9			0.3	273	80
A	Helotiales	Helotiales clone	Otu48			9	169			17	0.0			2	0.0	48
A	Helotiales	Dasyscyphus fuscescens	Daf			0.4	0.0	0.02	0.02	0.0	0.0			0.0	0.0	2681 (1.2%), 4269 (1.6%), ...
B	Microstromatales	Microstroma juglandis	Mij	0.09	0.88	0.9	5		0.02	0.0	2	0.13	0.65	0.0	0.0	517
A	Rhytismatales	Rhytisma_acerinum	Otu21			3	2			0.2	0.2			890	59	21
A	Pleosporales	Paraconiothyrium sp.	Pa1	2.92	2.21	418	645	1.10	0.44	28	58	0.39	0.65	1.0	0.3	15
A	Pleosporales	Boeremia sp.	Be	1.59	0.80	216	115	2.11	0.19	13	430	0.13		24	260	94 (2.3%), 55, 31, 129 (0.8%), ...
A	Pleosporales	Didymella macrostoma	Dim	1.41	1.86	201	610	0.27	0.02	4	196	0.26	0.77	30	682	14, 233 (1.1%), 772 (2.7%)
A	Pleosporales	Didymella vitalbina	Div	0.44	0.09	6	162		0.02	16	3	0.13		0.1	18	46, 1156 (2.7%), 132 (2.6%), ...
A	Pleosporales	Didymella sp.	Disp2			0.0	0.0			0.0	0.0		0.13	0.0	0.0	
A	Pleosporales	Didymella viburnicola	Div2			5	1	0.04		0.0	6			0.5	0.0	355
A	Pleosporales	Ascochyta pisi	Asp			6	78	0.33		0.7	81			9	243	57 (1.1%), 293 (0.8%), ...
A	Pleosporales	Epicoccum sp.	Epsp	0.35	0.09	0.0	0.0			0.0	0.0	0.52		0.0	0.0	
A	Pleosporales	Microsphaeropsis olivacea	Mio			7	43	0.03		34	37			2	133	106 (0.8%), 116, 3359 (1.9%)
A	Pleosporales	Phoma sp.	Phsp2			0.0	0.0	0.08		0.0	29			0.0	0.0	420 (0.8%)
A	Pleosporales	Phoma sp.	Phsp4			0.0	0.0	0.08		0.0	0.0			0.0	0.0	
A	Pleosporales	Pyrenochaeta spp.	Pyr		0.09	24	17	0.17	0.04	15	751			15	9	59 (0.8%), 118, 232 (2.3%)
A	Pleosporales	Alternaria spp.	Aa	1.06	0.71	91	151	0.50	0.05	1	2	1.42	0.39	8	36	54
A	Pleosporales	Ulocladium alternariae?	Ula	0.18		0.0	0.0			0.0	0.0			0.0	0.0	
A	Pleosporales	Pleospora spp.	Ple			0.2	0.0	0.04	0.13	0.0	0.0			0.0	0.0	2816
A	Pleosporales	Stemphylium vesicarium	Stv			5	0.8			0.0	0.0	0.13		0.1	0.0	474, 2165 (0.7%)
A	Pleosporales	Phaeosphaeria sp.	Phsp			0.6	0.1			0.0	604	0.13		0.2	0.1	47 (1.6%)
A	Pleosporales	Ampelomyces quisqualis	Aq			0.2	0.0	0.69		0.2	0.0			10	12	279, 6648 (1.9%), 6650 (2.3%)
A	Pleosporales	Coniothyrium sp.	Cosp5	0.09	0.18	0.0	0.0		0.03	0.0	0.0		0.39	0.0	0.0	
A	Pleosporales	Leptosphaeriaceae isolate	UnL			29	258		0.02	0.0	0.0			0.1	0.1	29 (0.5%)
A	Pleosporales	Pleosporales isolate	Ple2	0.35	0.09	4	37		0.04	5	204	0.26	0.26	8	53	130 (0.4%), 292 (0.8%), ...
A	Pleosporales	Pleosporales isolate	Pli2			20	61			0.0	0.0			1	0.0	125 (0.4%), 1298 (1.3%), ...
A	Pleosporales	Pleosporales isolate	UnP		0.09	0.0	0.0			0.0	0.0			0.0	0.0	
A	Pleosporales	Preussia minima	Prm			20	8		0.08	3	46			4	78	186 (2.4%), 343 (2.8%), ...
A	Pleosporales	Muriphaeosphaeria	Pha2		0.18	0.8	178	0.02		0.1	0.0		0.13	0.5	0.1	71, 398, 568 (2.3%), ...

		viburni / Phaeosphaeriaceae isolate														
A	Pleosporales	Phaeosphaeria nigrans	Phn		0.09	3	0.0			0.1	0.0	0.26	0.13	0.4	0.0	662, 3964 (1.5%)
A	Pleosporales	Phaeosphaeria sp.	Phsp3			2	76			0.0	19			0.4	1	98 (0.8%)
A	Pleosporales	Neosetophoma sp.	Nesp		0.18	19	13			11	0.0			4	35	150, 447 (0.8%), 417 (0.4%)
A	Pleosporales	Phaeosphaeriaceae clone	Otu82			0.1	0.0			0.0	0.0			1	159	82
A	Pleosporales	Setophoma sp., Phaeosphaeria lunariae	Pha			26	58			0.0	0.0			1	1	100, 396, 496 (0.4%), ...
A	Pleosporales	Periconia sp.	Otu52			0.1	154			0.0	0.1			0.0	0.0	52
A	Pleosporales	Periconia macrospinosa	Pem			0.0	0.0	0.02		0.0	0.0			0.0	0.0	
A	Pleosporales	Camarosporium sp.	Casp		0.09	0.7	3			0.0	0.0			0.0	0.0	603
A	Xylariales	Nemania diffusa / Xylaria spp.	Xyl	2.39	0.53	18	17	0.70	0.32	0.0	0.0	2.71	1.29	3	0.0	178, 139
A	Xylariales	Biscogniauxia spp.	Bn	0.44	0.09	3	2	0.72	0.29	0.0	0.0	0.77	0.39	0.4	0.0	533, 865, 5816
A	Xylariales	Kretzschmaria deusta	Kd	0.53	0.09	9	2	0.41	0.26	0.5	0.0	0.65	0.13	0.2	0.0	155
A	Xylariales	Annulohypoxylon spp.	Ann	0.09		0.0	0.0	0.19	0.02	0.0	0.0	0.13	0.52	0.0	0.0	
A	Xylariales	Rosellinia spp.	Rc	0.53	0.18	2	0.0	0.17	0.02	0.4	0.0			0.0	0.0	1424 (0.4%), 2860, 4048 (1.4%), ...
A	Xylariales	Xylariaceae isolate / Nemania serpens	Ns	0.18	0.18	2	0.0	0.13		0.0	0.0	0.26	0.13	0.1	0.0	189, 272, 6620, 389 (0.4%), ...
A	Xylariales	Daldinia childiae	Dac	0.44		21	5	0.26	0.10	0.1	0.0			2	12	188
A	Xylariales	Hypoxylon fragiforme	Hyf2	0.18	0.18	3	4			0.0	0.0	0.26		0.5	0.0	387
A	Xylariales	Hypoxylon rubiginosum	Hyr			1	4	0.08	0.08	0.0	0.0	0.13		1.0	6	193, 546
A	Xylariales	Xylariaceae isolate	Rosp	0.18		0.1	0.0	0.20	0.17	0.1	0.0	0.26		0.0	3	149
A	Xylariales	Xylariaceae isolate	UnX			1	17	0.02		1	43			1	68	170
A	Xylariales	Xylariaceae isolate	Xyi			0.0	0.0			0.0	0.0			0.0	0.0	6227 (2.6%)
A	Xylariales	Creosphaeria sassafras	Crs			0.0	0.0	0.02	0.02	0.0	0.0			0.0	0.0	
A	Xylariales	Creosphaeria sassafras	Crs2			0.0	0.0		0.02	0.0	0.0			0.0	0.0	
A	Xylariales	Lopadostoma turgidum	Lot			1	3			0.0	2			0.1	0.1	464, 2989 (1.8%)
A	Xylariales	Xylaria polymorpha	Xyp			0.0	0.0			0.0	0.0			0.0	0.0	611
A	Dothideales	Aureobasidium pullulans	Aup		0.80	19	179	0.13	0.09	1	89	0.26	0.65	2	47	34, 764 (2.3%), 2536 (1.9%)
A	Dothideales	Aureobasidium subglaciale	Aus		0.09	0.0	0.0			0.0	0.0			0.0	0.0	
A	Dothideales	Dothideaceae clone	Otu85			0.2	105			0.0	0.0			0.0	0.0	85
B	Malasseziales	Malassezia sp.	Otu40			5	84			4	170			4	479	40
B	Malasseziales	Malassezia_globosa	Otu288			7	68			0.8	86			4	215	288
B	Malasseziales	Malassezia_globosa	Otu164			3	53			0.8	63			2	187	164
B	Malasseziales	Malassezia_globosa	Otu210			2	33			1	105			0.7	67	210

A	Sordariales	Neurospora sp.	Otu51			5	86			2	25			9	162	51
A	Pleosporales	Nodulosphaeria digitalis	Nod			3	6			0.5	0.0			0.0	0.0	401 (0.8%), 676
A		Ascomycete isolate	UnA			3	5			0.0	0.0	0.26	0.13	0.0	5	327 (0.8%)
A	Dothideomycetes_ ord_Incertae_sedis	Leptospora_rubella	Otu58			1	237			0.2	0.0			0.0	0.0	58
A	Myriangiales	Sphaceloma sp.	Otu161			15	109			0.2	105			7	62	161
A	Myriangiales	Elsinoe sp.	Elsp			13	0.0	0.09	0.02	2	46			1.0	0.0	237
B	Tremellales	Cryptococcus_frias	Otu67			0.4	183			0.0	34			0.0	0.0	67
B	Tremellales	Vishniacozyma_heimae yensis	Otu73			3	158			0.0	0.1			0.0	0.0	73
A	Hypocreales	Fusarium sp.	Fusp	0.09	0.09	1	112	0.02	0.11	0.1	0.0		0.39	0.4	0.2	97
A	Hypocreales	Fusarium tritinctum	Fut	0.18		0.2	8			0.0	0.1	0.13	0.39	1.0	5	77
A	Hypocreales	Fusarium sp. xy	Fusp2	0.09	0.09	0.0	0.0			0.0	0.0			0.0	0.0	
A	Hypocreales	Cosmospora arxii	Coa		0.18	0.0	0.0			0.0	0.0			0.0	5	774 (3%), 3495 (0.4%)
A	Hypocreales	Nectria dematiosa	Ned			0.0	0.0			0.0	0.0		0.13	0.0	0.7	1846
A	Hypocreales	Stylonectria sp.	Stsp			0.0	0.0			0.0	0.0	0.13		0.0	0.0	
A	Hypocreales	Clonostachys rosea	Clr		0.09	0.0	0.0			0.0	0.0	0.13		0.0	0.0	1332 (1.8%)
A	Hypocreales	Sarocladium spp.	Sar			1.0	22			0.0	9			0.0	30	216, 451, 5109 (1.4%)
A	Hypocreales	Simplicillium lamellicola	Sil			0.0	0.0			0.2	0.0			0.0	0.0	5328
B	Polyporales	Polyporales clone	Otu68			4	176			0.0	0.1			0.0	0.1	68
B	Polyporales	Trametes hirsuta	Trh		0.27	0.0	4			0.0	0.0			0.0	2	548 (0.7%)
A		Pezizomycetes sp.	Pesp		0.09	0.0	0.0			0.0	0.0	0.13		0.0	0.0	
A	Saccharomycetales	Saccharomycetales clone	Otu83			0.0	120			0.0	0.0			0.4	0.1	83
B	Agaricales	Clitocybe_nebularis	Otu101			0.0	185			0.0	0.0			0.0	0.0	101
B	Agaricales	Schizophyllum commune	Scc		0.09	0.0	15			0.0	4			0.0	0.0	493 (0.3%), 818, 1605 (0.3%), ...
B	Agaricales	Hypholoma fasciculare	Hyf			0.5	0.0			0.0	0.0			0.0	0.0	373
A	Calosphaeriales	Jattaea sp.	Jasp			0.0	0.0		0.02	0.0	0.0			0.0	0.0	
A		Sordariomycetes isolate	Soi			0.0	0.0			0.0	0.0			0.2	0.0	2079
